# Retinoic acid signaling maintains epithelial and mesenchymal progenitors in the developing mouse ureter

**DOI:** 10.1038/s41598-017-14790-2

**Published:** 2017-11-01

**Authors:** Tobias Bohnenpoll, Anna-Carina Weiss, Maurice Labuhn, Timo H. Lüdtke, M.-O. Trowe, Andreas Kispert

**Affiliations:** Institut für Molekularbiologie, Medizinische Hochschule Hannover, 30625 Hannover, Germany

## Abstract

The differentiated cell types of the mature ureter arise from the distal ureteric bud epithelium and its surrounding mesenchyme. Uncommitted epithelial cells first become intermediate cells from which both basal and superficial cells develop. Mesenchymal progenitors give rise to separated layers of adventitial fibrocytes, smooth muscle cells and lamina propria fibrocytes. How progenitor expansion and differentiation are balanced is poorly understood. Here, we addressed the role of retinoic acid (RA) signaling in these programs. Using expression analysis of components and target genes, we show that pathway activity is restricted to the mesenchymal and epithelial progenitor pools. Inhibition of RA signaling in ureter explant cultures resulted in tissue hypoplasia with a relative expansion of smooth muscle cells at the expense of lamina propria fibroblasts in the mesenchyme, and of superficial cells at the expense of intermediate cells in the ureteric epithelium. Administration of RA led to a slight reduction of smooth muscle cells, and almost completely prevented differentiation of intermediate cells into basal and superficial cells. We identified cellular programs and transcriptional targets of RA signaling that may account for this activity. We conclude that RA signaling is required and sufficient to maintain mesenchymal and epithelial progenitors in early ureter development.

## Introduction

The mammalian ureter is a tubular organ dedicated to the efficient removal of the urine from the renal pelvis to the bladder. Its outer mesenchymal coat is highly flexible and peristaltically active due to a three-layered organization of an outer fibroblastic tunica adventitia, a medial accumulation of smooth muscle cells (SMCs) and an inner lamina propria with fibroblasts. The inner epithelial compartment, the urothelium, is a three-tiered dilatable yet sealing tissue with a single layer of cuboidal basal cells (B-cells), one or two layers of similarly shaped intermediate cells (I-cells), and a luminal layer of large squamous superficial cells (S-cells)^1^.

The ordered array of differentiated cell types of the mature ureter arises by a complex program of patterning, proliferation and differentiation from epithelial and mesenchymal precursor cells in early embryogenesis. In the mouse, a diverticulum of the nephric duct emerges around E10.5 at the level of the future hindlimbs and invades the adjacent mesenchymal tissue. While the proximal aspect of the bud and the surrounding mesenchyme will form the different tissues of the kidney, the distal cell populations are specified towards a ureteric fate. From E12.5 to E14.5, the ureteric mesenchyme is subdivided into an inner and outer region that will differentiate from E15.5 onwards in a proximal to distal wave into SMCs and adventitial fibroblasts, respectively. The lamina propria can be recognized from E16.5 onwards as a cell-sparse layer in between the ureteric epithelium and the SMCs. The ureteric epithelium remains single-layered and undifferentiated until E14.5. Around this stage, I-cell markers are switched on and stratification occurs. At E16.5, the luminal layer of epithelial cells starts S-cell differentiation while individual cells of the basal layer initiate B-cell differentiation. Around birth, three distinct layers with differentiated B-, I- and S-cells can be distinguished^2^.

While the signals and factors that maintain the precursor character of the ureteric tissues have remained enigmatic, embryological and genetic experiments have shown that mesenchymal and epithelial differentiation is coupled by trans-acting signaling activities. Epithelial WNT signals are required for SMC differentiation while epithelial SHH induces a mesenchymal FOXF1-BMP4 module to direct SMC and urothelial differentiation^3–6^.

Retinoic acid (RA) signaling is a conserved paracrine pathway with diverse functions in cellular proliferation and differentiation programs both in embryogenesis and in adult tissue homeostasis^7,8^. RA is locally produced from dietary retinol (vitamin A) by two consecutive oxidation steps catalyzed by retinol and retinaldehyde dehydrogenases^8^. RA binds to nuclear RA receptors (RARs) that form heterodimers with retinoid X receptors (RXRs) which triggers the exchange of co-repressor against coactivator complexes to activate the expression of target genes^9,10^. Previous studies indicated diverse roles of RA signaling in the excretory system. RA from the pericloacal mesenchyme regulates ureter-bladder connectivity by inducing apoptosis of the most distal aspect of the nephric duct^11^. RA signaling is active in the renal stroma to regulate branching morphogenesis^12,13^. Studies in the bladder have recently uncovered a role for the signal in urothelial specification^14^.

Here, we analyze the functional involvement of RA signaling in the development of the murine ureter. We uncover a critical role for this pathway in the maintenance of progenitor cells in the early ureter and identify RA-responsive genes that may account for this function.

## Results

### RA signaling is active in early ureter development

**Figure 1:**
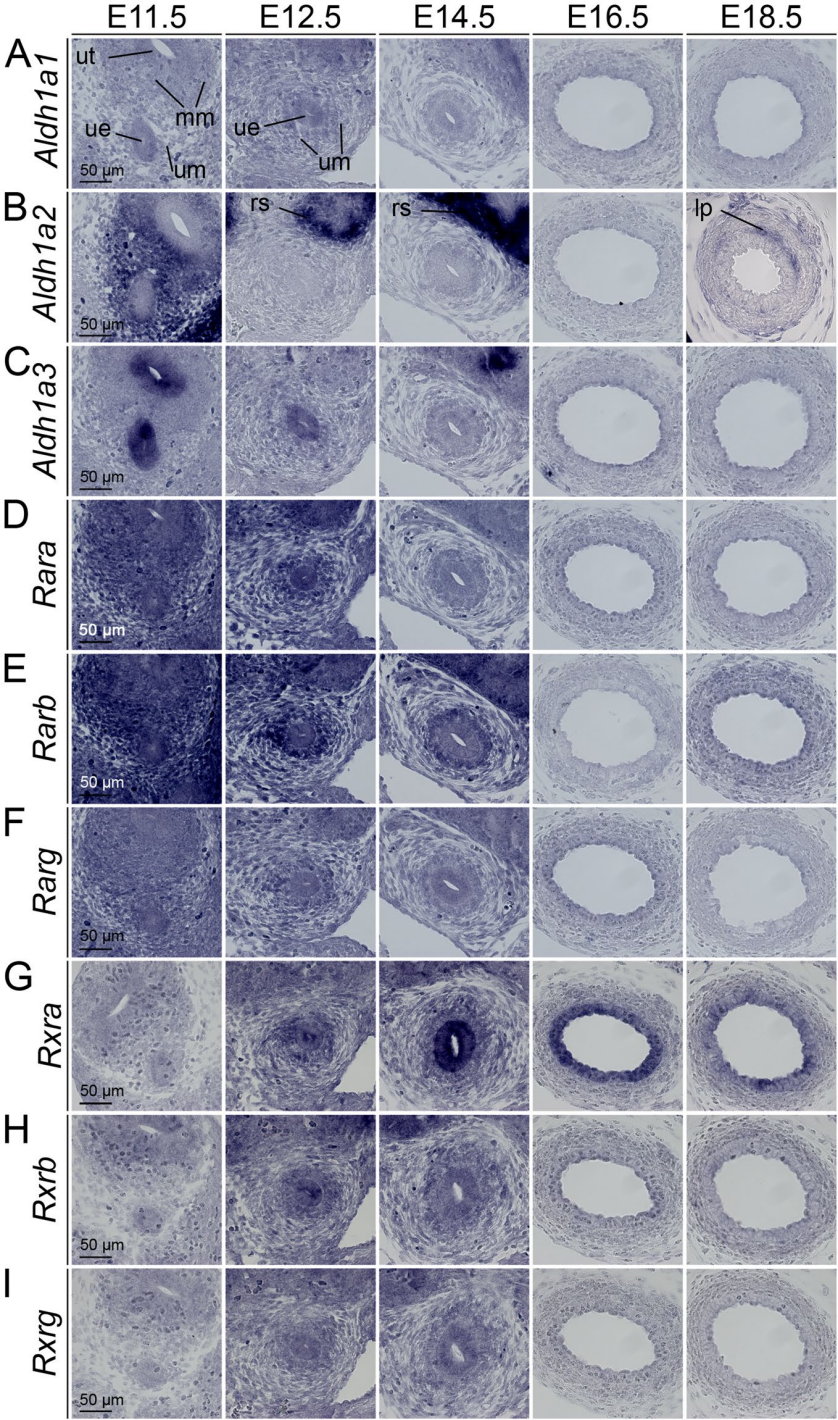
RA signaling is active in early ureter development. *In situ* hybridization analysis on transverse sections of the proximal ureter of wild-type embryos for expression of genes encoding RA synthesizing genes (**A**–**C**), RA receptors (**D**–**F**) and retinoid X receptors (**G**–**I**). Stages and probes are as indicated. For explanations see main text. lp, lamina propria; mm, metanephrogenic mesenchyme; rs, renal stroma; ue, ureteric epithelium; um, ureteric mesenchyme; ut, ureter tip.

To determine the spatiotemporal profile of RA signaling in ureter development, we first analyzed by *in situ* hybridization on proximal ureter sections the expression of genes responsible for the local production of RA from retinaldehyde (*Aldh1a1*, *Aldh1a2*, *Aldh1a3*). *Aldh1a2* was found throughout the ureteric mesenchyme at E11.5. Expression was absent in the proximal ureter from E12.5 to E16.5 but was reactivated in the lamina propria at E18.5 (Fig. 1A). Analysis of whole urogenital systems showed that *Aldh1a2* expression persisted in the mesenchyme of the distal most aspect of the ureter close to the forming bladder (Supplementary Fig. S1). Additional expression of *Aldh1a2* was found in the renal stroma (Fig. 1A, Supplementary Fig. S1 and data not shown). *Aldh1a3* expression was restricted to the epithelium of the nephric duct and the ureteric bud at E11.5. Expression was maintained in the renal collecting duct system but was lost in the ureter except its distal most aspect at subsequent stages (Fig. 1A–C, Supplementary Fig. S1). We next screened for expression of genes encoding RARs (*Rara*, *Rarb*, *Rarg*) and RXRs (*Rxra*, *Rxrb*, *Rxrg*) that account for transcriptional activation of target genes upon RA binding. Expression of all three *Rar* genes was found in the ureteric epithelium and mesenchyme at E11.5 and E12.5. Expression of *Rarb*, a direct transcriptional target of this pathway^15^, was maintained at E14.5, particularly in the inner mesenchymal domain whereas expression of *Rara* and *Rarg* was down-regulated at this stage (Fig. 1D–F). Expression of all three *Rxr* genes was detected at E11.5 to E14.5 both in the ureteric epithelium and mesenchyme. *Rxra* was increased in the ureteric epithelium at E14.5 and was maintained in this domain until E18.5 (Fig. 1G–I). Together, this analysis shows that RA is synthesized in both tissue compartments of the ureter at E11.5, and that RA signaling occurs therein until E14.5, i.e. prior to the onset of ureteric cell differentiation.

### RA signaling controls differentiation of the ureteric mesenchyme

To interrogate the functional involvement of RA signaling in ureter development, we used pharmacological pathway inhibition and activation approaches in explant cultures of kidney and ureter rudiments. Such an approach does not only allow for strong reduction and activation of the pathway, it also provides the possibility to analyze the temporal window of pathway activity with greater ease and less animal consumption compared to a genetic approach that has to consider redundancy of *Rar* and *Rxr* gene function. For inhibition of RA signaling, we used the pan RAR antagonist BMS493^16^ in a concentration of 1 µM after showing that this concentration strongly reduced expression of the RA target gene *Rarb* in E11.5 explants after 18 h of treatment. (Over-)activation of the pathway was reached by 1 µM RA in the same interval (Supplementary Fig. S2). Notably, similar or identical concentrations of these compounds were previously successfully used in kidney explants and other organ contexts to manipulate RA signaling^13,16–18^.

We explanted ureter rudiments at E11.5, E12.5, E14.5, E16.5 and E18.5 and cultured them for 14, 12, 10, 8 and 6 days to obtain similar end-points for histological and molecular analyses. We first investigated the effect of RA manipulation on mesenchymal cell types. We have recently shown that the T-box transcription factor gene *Tbx18* is expressed in the undifferentiated ureteric mesenchyme, and that the descendants of this expression domain constitute the ureteric mesenchymal wall throughout development and in adulthood^19,20^. We therefore used mice double heterozygous for a *cre* knock-in the *Tbx18* locus and the *Rosa26* ^*mTmG*^ reporter line (*Tbx18* ^*cre/*+^; *Rosa26* ^*mTmG/*+^)^21,22^ for visualization of the ureteric mesenchyme by GFP expression.

Brightfield microscopical analysis throughout the culture period showed that the ureter elongated and became peristaltically active under all conditions. The layer of strong GFP epifluorescence surrounding the ureteric epithelium appeared decreased under BMS treatment and increased under RA treatment at the end of the culture period of ureter explants at E11.5, E12.5 and E14.5 while no changes were recognized for explants from E16.5 and E18.5 embryos (Supplementary Figs S3–7). Quantification of the thickness of the mesenchymal wall and of its cellularity confirmed a trend for opposing effects of BMS and RA treatment on these parameters which, however, only gained significance for both parameters in BMS treated explants of E12.5 embryos cultured for 12 days (Supplementary Figs S8,9).

**Figure 2:**
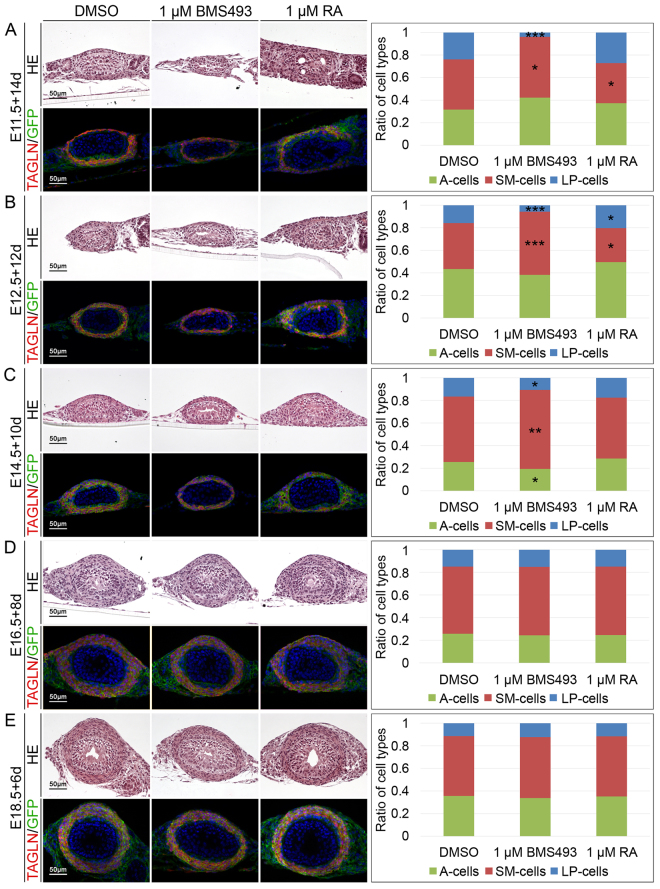
RA signaling controls ureteric mesenchymal differentiation. Hematoxylin and eosin (upper panel) and co-immunofluorescence analysis of GFP and TAGLN (lower panel) on transverse proximal sections of E11.5 + 14d (**A**), E12.5 + 12d (**B**), E14.5 + 10d (**C**), E16.5 + 8d (**D**) and E18.5 + 6d (**E**) *Tbx18* ^*cre/*+^; *R26* ^*mTmG/*+^ ureter explants that were treated with DMSO, 1 µM BMS493 or 1 µM RA. The bar graph displays ratios of differentiated cell types that were quantified based on the immunofluorescence analysis. RA signaling acts from E11.5 to E14.5 to maintain lamina propria cells at the expense of SMCs. For numbers and statistics see Supplementary Table S1. *p ≤ 0.01; **p ≤ 0.001; ***p ≤ 0.0001.

To detect and quantify cell differentiation in the ureteric mesenchyme, we analyzed co-expression of the SMC marker Transgelin (TAGLN) with the GFP reporter by immunofluorescence on sections of ureter explants of *Tbx18* ^*cre/*+^ *; Rosa26* ^*mTmG/*+^ mice. Adventitial fibroblasts were defined as GFP^+^TAGLN^−^ outer ring cells, SMCs as GFP^+^TAGLN^+^ intermediary cells and lamina propria cells as GFP^+^TAGLN^−^ inner ring cells of the mesenchymal wall. Inhibition of RA signaling in cultures of E11.5 to E14.5 ureter explants resulted in a relative increase of SMCs largely at the expense of lamina propria cells. At the same stages RA treatment led to a reduction of SMCs and increased lamina formation (most obviously at E12.5). In E16.5 and E18.5 explants, neither BMS nor RA treatment affected the composition of differentiated cell types in the mesenchymal wall of the ureter (Fig. 2, Supplementary Table S1). Hence, RA signaling is required from E11.5 to E14.5 for expansion of the ureteric mesenchyme, and for promotion of lamina fibrocytes at the expense of SMC fates.

### RA signaling controls urothelial differentiation

**Figure 3:**
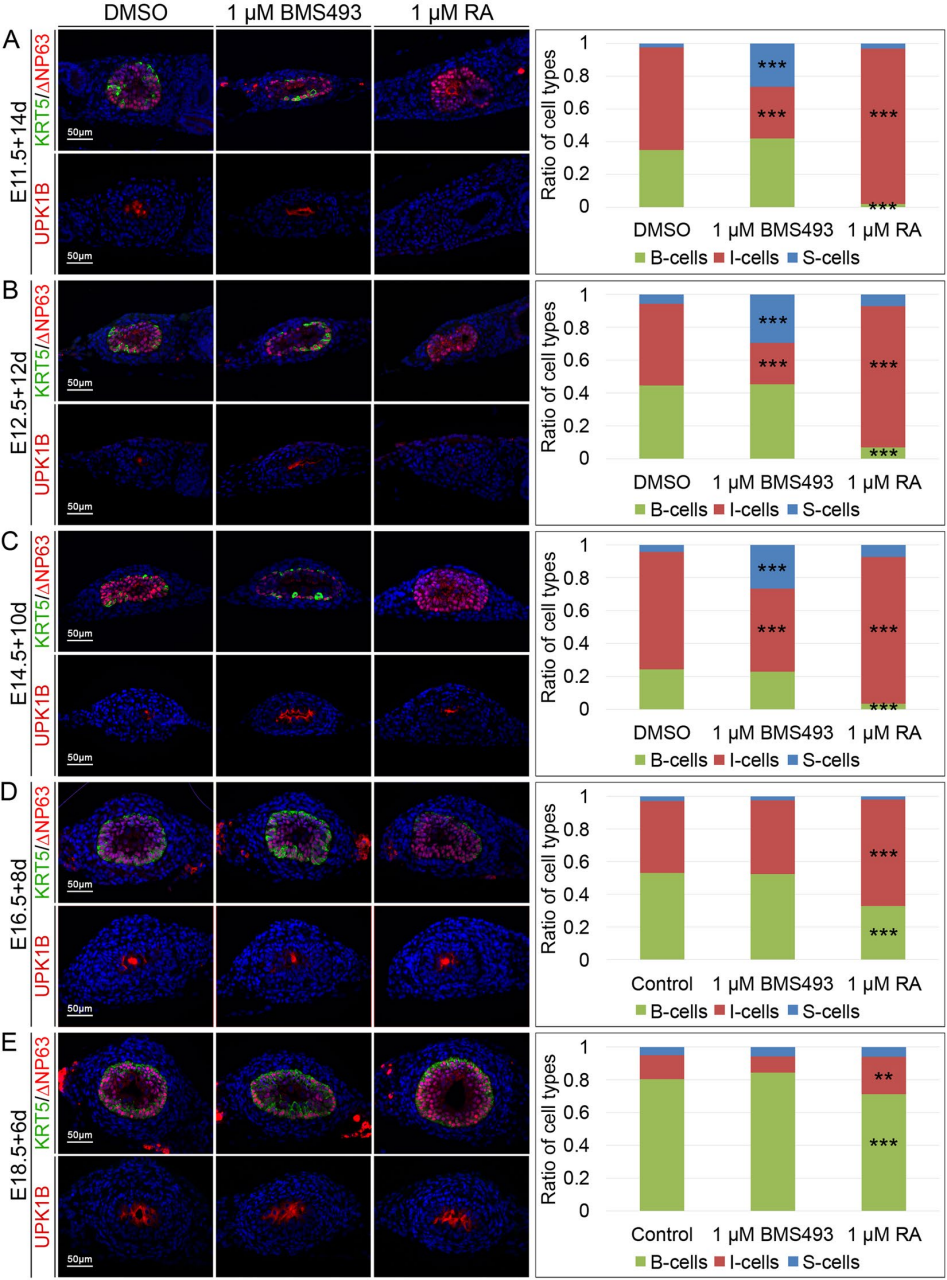
RA signaling controls urothelial differentiation. Co-immunofluorescence analysis of KRT5 and ∆NP63 (upper panel) and UPK1B (lower panel) on transverse proximal sections of E11.5 + 14d (**A**), E12.5 + 12d (**B**), E14.5 + 10d (**C**), E16.5 + 8d (**D**) and E18.5 + 6d (**E**) wildtype ureter explants that were treated with DMSO, 1 µM BMS493 or 1 µM RA. The bar diagram displays ratios of differentiated cell types that were quantified based on the immunofluorescence analysis. RA signaling acts from E11.5 to E14.5 to maintain I-cells at the expense of S-cells. Throughout all stages analyzed ectopic RA prevents the differentiation of B-cells. For numbers and statistics see Supplementary Table S2. **p ≤ 0.001; ***p ≤ 0.0001.

The three cell types of the mature ureteric epithelium can be distinguished by combinatorial expression of the intracytoplasmic protein KRT5, the nuclear protein ∆NP63 and the cell surface protein UPK1B. B-cells are KRT5^+^∆NP63^+^UPK1B^−^, I-cells are KRT5^−^∆NP63^+^UPK1B^+low^ and S-cells are KRT5^−^∆NP63^−^UPK1B^+high^ ^2^. Inhibition of RA signaling by BMS493 treatment resulted in E11.5, E12.5 and in E14.5 but not in E16.5 and E18.5 explants in a large expansion of S-cells at the expense of I-cells. Enhanced and prolonged RA signaling by RA administration prevented B-cell differentiation, reduced S-cell differentiation and expanded the pool of I-cells. In E16.5 and E18.5 explants, RA treatment expanded I-cells at the expense of B-cells (Fig. 3, Supplementary Table S2). BMS treatment did not affect tissue thickness and cellularity in any of these explants whereas RA treatment resulted in a small but significant increase in these parameters at E14.5 only (Supplementary Figs S8,9). Given our recent finding that in the ureter I-cells are precursors for both B- and S-cells^2^, this analysis suggests that RA signaling is dispensable for epithelial expansion but is required from E11.5 to at least E14.5 to prevent premature differentiation of I- into S- and B-cells.

### RA signaling has a minor function in proliferation control

**Figure 4:**
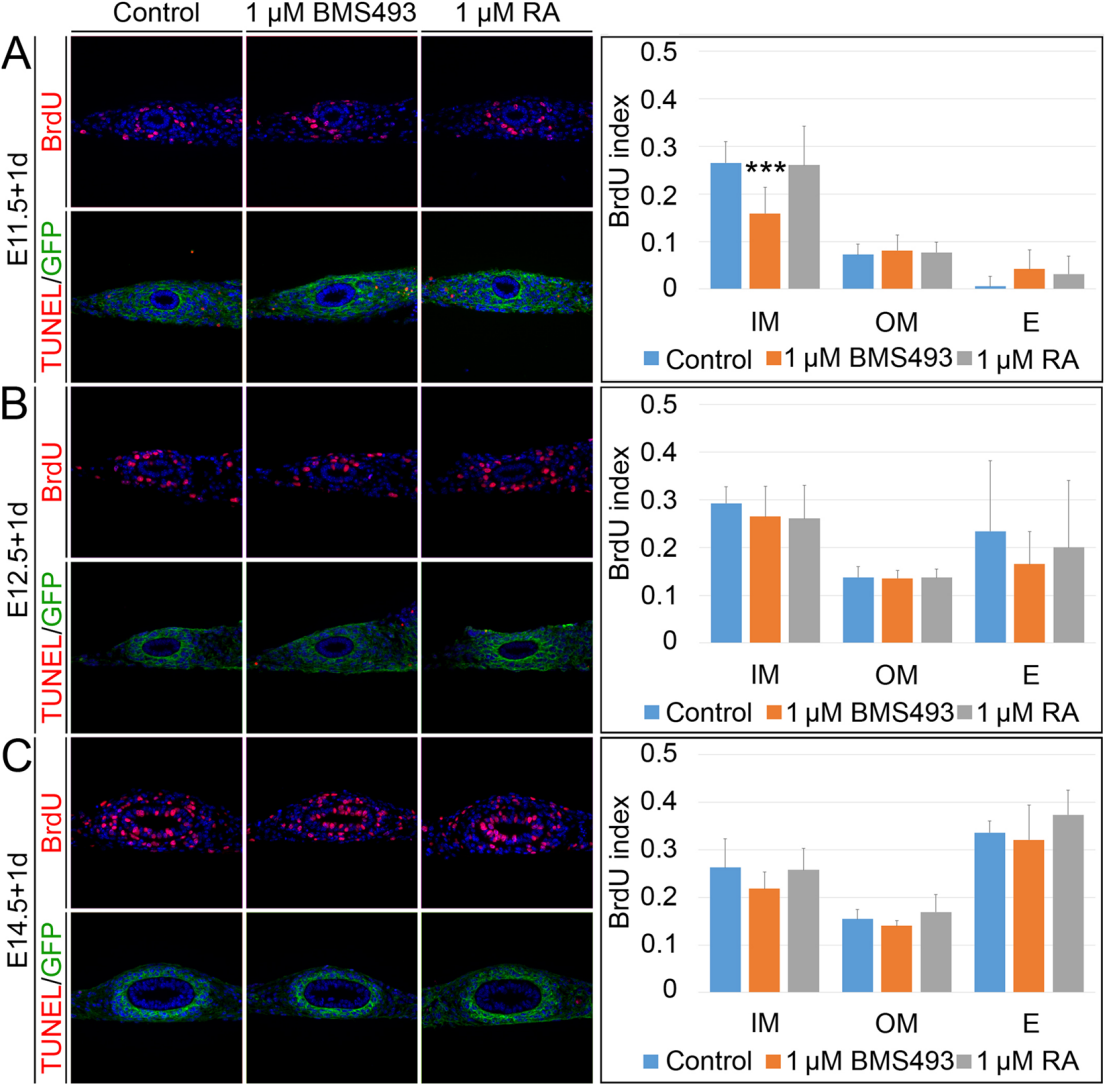
RA signaling affects proliferation in a minor way. Determination of cellular proliferation by BrdU incorporation assay (upper row) and apoptosis by TUNEL/GFP assay (lower row) on transverse sections of the proximal ureter of E11.5 (**A**), E12.5 (**B**) and E14.5 (**C**) *Tbx18* ^*cre/*+^ *; R26* ^*mTmG/*+^ explants that were cultured in the presence of DMSO, 1 µM BMS493 or 1 µM RA for 1 day. RA signaling is required to maintain proliferation of inner mesenchymal cells at E11.5. The bar diagrams display the BrdU incorporation indices in arbitrarily defined compartments of the ureter under given conditions. For numbers and statistics see Supplementary Table S3. ***p ≤ 0.0001. E, epithelium; IM, inner mesenchyme; OM, outer mesenchyme.

We next addressed a potential function of RA signaling in the proliferation and survival of the epithelial and mesenchymal tissue compartments of the ureter by performing bromodeoxyuridine (BrdU) incorporation assays (Fig. 4A–C, upper row, Supplementary Table S3) and terminal dUTP nick end-labeling (TUNEL) assays (Fig. 4A–C, lower row) on E11.5, E12.5 and E14.5 *Tbx18* ^*cre/*+^ *; R26* ^*mTmG/*+^ explants that were cultured in the presence of 1 µM BMS493 or 1 µM RA for 1 day. At E11.5, inhibition of RA signaling resulted in a significant decrease of proliferation of the inner mesenchymal compartment of the ureter but left the outer mesenchymal and epithelial cells unaltered. Activation of RA signaling showed no effect on proliferation rates, as did inhibition or activation experiments at E12.5 and E14.5. Moreover, apoptosis was not altered under RA loss- and gain-of-function conditions. We conclude that RA signaling has a minor function in proliferation control at E11.5.

### RA signaling prevents precocious differentiation of ureteric progenitors

**Figure 5:**
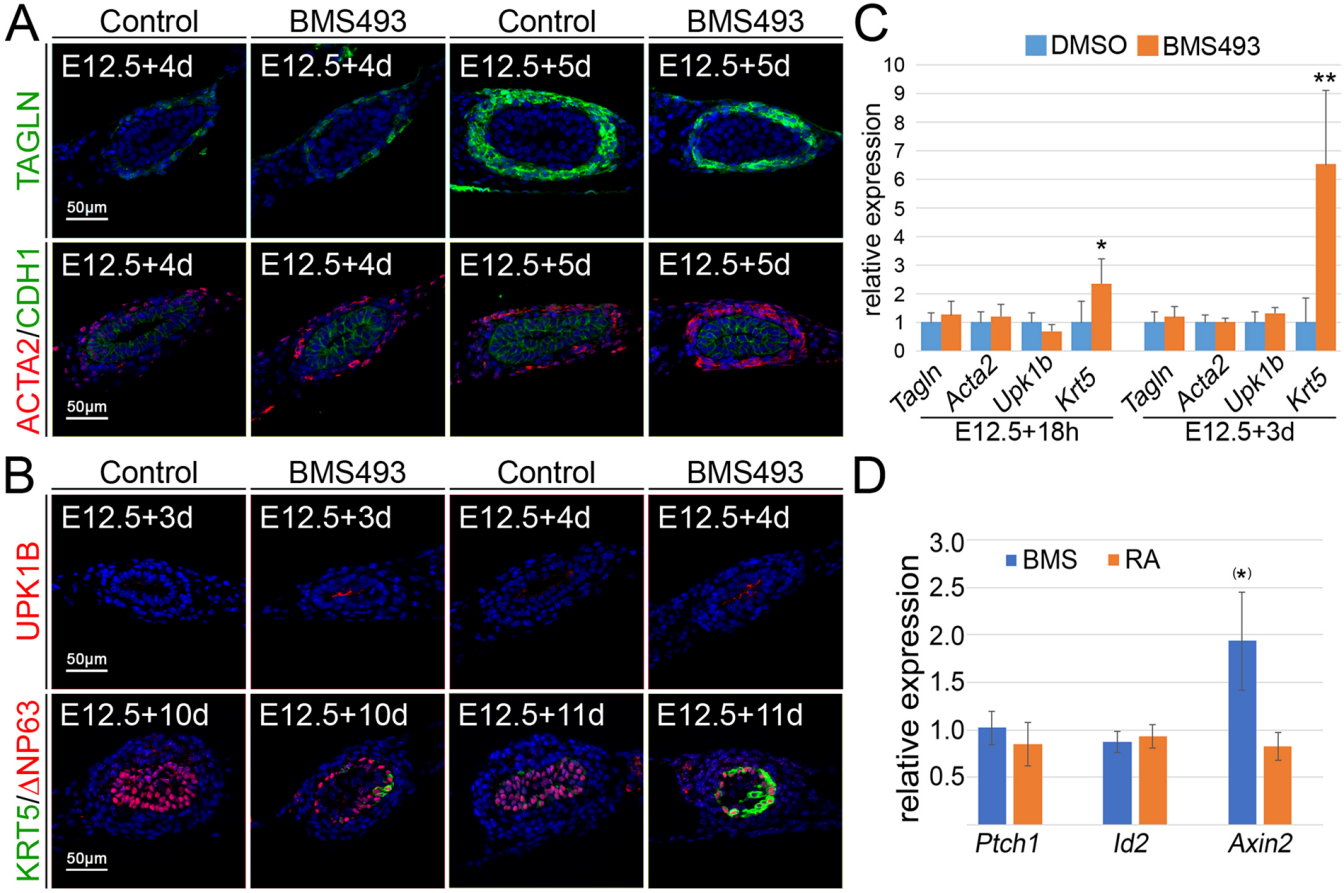
RA signaling prevents precocious differentiation in the ureter. (**A**) Immunofluorescence analysis for TAGLN (upper row) and co-immunofluorescence analysis of ACTA/CDH1 (lower row) on E12.5 ureter explants that were cultured for 3 and 4 days in presence of DMSO or 1 µM BMS493 shows that SMCs markers are slightly enhanced in their expression when RA signaling is abrogated. (**B**) Immunofluorescence analysis for UPK1B (upper row) and co-immunofluorescence analysis of KRT5/∆NP63 (lower row) on E12.5 ureter explants that were cultured for 3 and 4 days (UPK1B) or 10 and 11 days (KRT5/∆NP63) in the presence of DMSO or 1 µM BMS493. Markers for S-cells (UPK1B) and B-cells (KRT5) are prematurely expressed when RA signaling is inhibited. (**C**) RT-PCR analysis of expression of the SMC marker *Tagln and Acta2*, the S-cell marker *Upk1b* and the B-cell marker *Krt5* in ureters treated for 18 h and 3 days with DMSO or the RA signaling inhibitor BMS493. Note the strong increase of expression of *Krt5* at both stages. (**D**) RT-PCR analysis of expression of the SHH target gene *Ptch1*, of the WNT target gene *Axin2* and the BMP target gene *Id2* in ureters treated for 18 h with DMSO, 1 µM BMS493 or 1 µM RA. Note increase of expression of *Axin2* almost reaching significance as indicated by (*). For numbers and statistics (for **C** and **D**) see Supplementary Table S4 and 5. *p ≤ 0.01; **p ≤ 0.001.

To further test whether RA signaling contributes to the maintenance of precursor cells, we explanted E12.5 ureters and cultured them for different intervals to score the onset of cell differentiation in the mesenchymal and epithelial compartments. SMC differentiation, as analyzed by TAGLN (Fig. 5A, upper row) and ACTA2 (Fig. 5A, lower row) immunofluorescence, was initiated after 4 days in control cultures. TAGLN showed a comparable expression in BMS493 treated cultures after 4 and 5 days, but ACTA2 expression was considerably stronger under these conditions at both time-points. Expression of UPK1B and KRT5 indicated onset of S-cell and B-cell differentiation after 4 and 11 days, respectively, in control cultures. Both markers were expressed 1 day earlier in BMS493 treated cultures (Fig. 5B, upper and lower row). To confirm these findings in an independent assay, we performed RT-PCR analysis on RNA isolated from E12.5 ureters treated for 18 h and 3 days, respectively, with 1 µM BMS. After 18 h, *Upk1b* was slightly decreased, expression of *Tagln* and *Acta2* was weakly but not significantly increased, levels of *Krt5* mRNA were strongly and significantly increased. After three days, there was a weak trend for increased *Upk1b*, *Tagln* and *Acta2* expression. *Krt5* expression was even more enhanced under BMS treatment (Fig. 5C, Supplementary Table S4).

Previous work implicated SHH and BMP4 in both epithelial and mesenchymal differentiation, and WNT signaling in mesenchymal cell differentiation in ureter development^5,6,23^. To analyze whether dysregulation of any of these pathways underlies the observed premature cytodifferentiation when RA signaling is inhibited, we determined expression of the *bona fide* target genes of these pathways, *Ptch1, Axin2* and *Id2* ^24–26^, in E12.5 ureters cultured for 18 h with BMS493. While *Ptch1* and *Id2* expression was unchanged, *Axin2 e*xpression was two-fold upregulated. Addition of 1 µM RA led to a slight but not significant reduction of expression of all of these genes (Fig. 5D, Supplementary Table S5). Together, these findings suggest a role for RA signaling in suppressing differentiation of epithelial progenitors into S- and most strongly into B-cells, and to attenuate SMC differentiation of mesenchymal progenitors. Since canonical WNT signaling acts in the mesenchyme to induce SMCs from precursors^23^, its increased activity (as indicated by increased *Axin2* expression) may at least partly contribute to premature SMC differentiation when RA signaling is inhibited.

### Characterization of the RA-responsive transcriptome in early ureter development

To get more insights into the phenotypic changes and the underlying molecular programs that are controlled by RA signaling in the early ureter, we treated E12.5 ureters with 1 µM BMS493 or 1 µM RA for 18 h and performed microarray analysis of global differential gene expression compared to untreated controls.

We first addressed our hypothesis that RA signaling maintains the progenitor status in the mesenchymal and epithelial compartments of the ureter by examining transcripts that were negatively regulated by RA signaling. Using an intensity threshold of 100, we identified in three independent pools of treated and untreated ureters a set of 554 genes that were consistently more than 1.2 fold increased in their expression upon BMS treatment and 612 genes that were consistently more than 1.2 fold decreased in RA treated ureters yielding a set of 261 genes that were negatively regulated by RA signaling (Supplementary Fig. S10A, Supplementary Tables S6-8). GO enrichment analysis did not provide conclusive insight into causative changes of cellular or molecular pathways (Supplementary Table S9). Among the most affected transcripts were two cytokeratins which are known to be expressed in urothelial B-cells, namely *Krt6a* (4.2x) and *Krt5* (2.1x)^2,27^ as well as transcripts encoding for structural components of SMCs, including *Actg2* (1.6x), *Acta1* (1.5x), *Actc1* (1.4x) and *Tagln* (1.3x) (Supplementary Fig. S10B), providing further evidence for a functional requirement of RA signaling in preventing precocious cell differentiation in the ureter. To validate the RA-responsive expression of top-deregulated genes and to characterize their spatial confinement we performed RNA *in situ* hybridization analysis on explants of E12.5 kidney/ureter rudiments cultured for 1 day in the presence of 1 µM BMS493. We found weak upregulation of *Rgcc*, *Serpine1* and *Dkk1* in the ureteric epithelium (Supplementary Fig. S10C).

**Figure 6:**
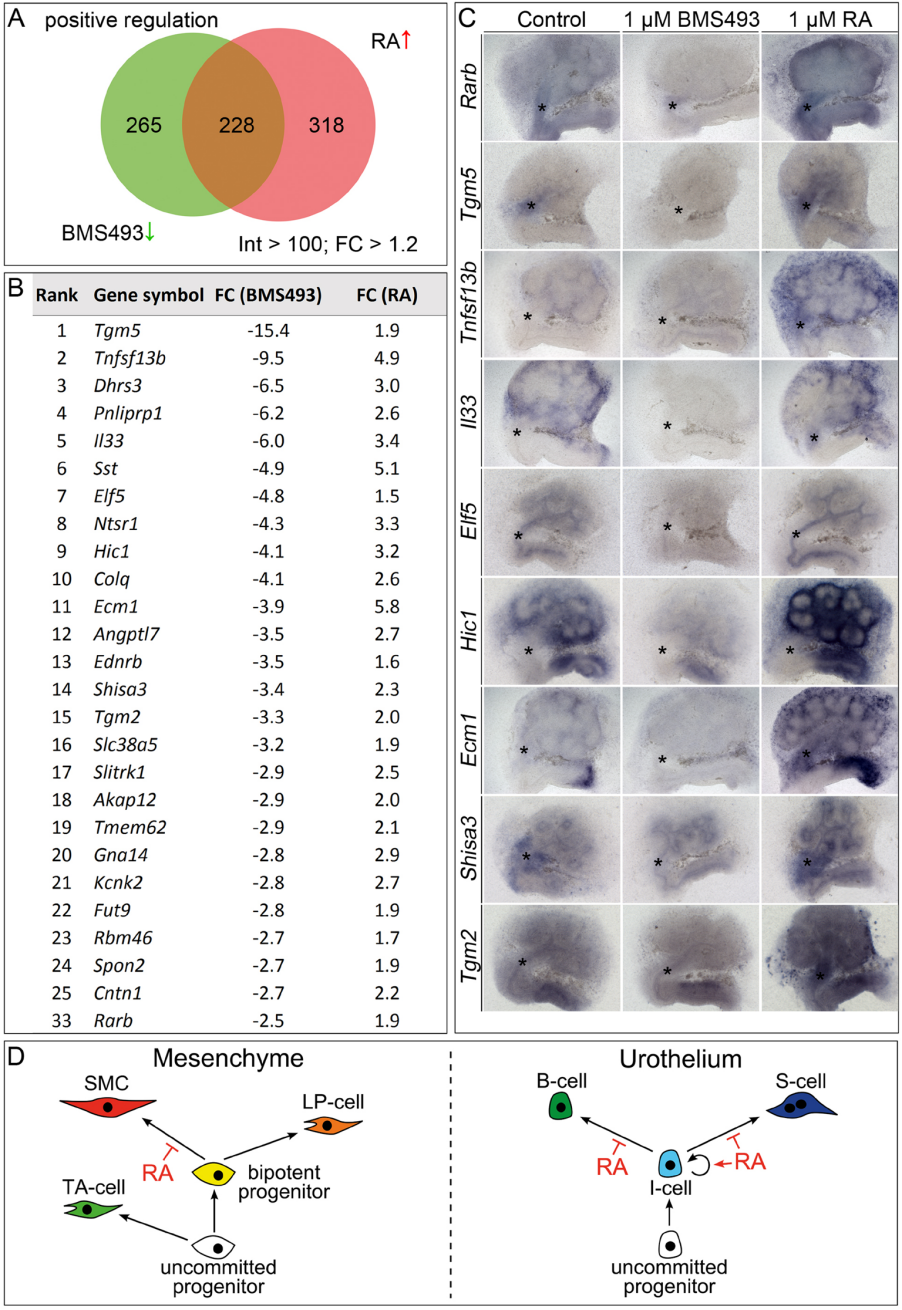
Identification of genes positively regulated by RA signaling in microarray experiments. Summary of the results from the microarray analysis of E12.5 ureters explanted and treated with DMSO or 1 µM BMS493 or 1 µM RA for 18 h filtered with an intensity (Int) threshold of 100 and a fold change (FC) cut-off of 1.2. (**A**) The Venn diagram displays transcripts which were positively regulated by RA signaling, i.e. down-regulated upon BMS493 treatment (493, green) or up-regulated upon RA treatment (546, red). The intersection shows the common group of positively regulated transcripts (228). (**B**) Top 25 of the common positively regulated genes were ranked according to their FC down-regulation upon BMS493 treatment. (**C**) *In situ* hybridization analysis of expression of selected genes which were positively regulated by RA signaling on E11.5 kidney explant cultures, treated with 1 µM BMS493 or 1 µM RA for 1 day. (**D**) Schematic illustration of RA signaling function in the maintenance of ureteric progenitors. In the ureteric mesenchyme (left) uncommitted progenitors diversify into adventitial fibrocytes (AF-cells) and bipotent progenitors of SMCs and lamina propria fibrocytes (LP-cells). RA specifically acts in the bipotent progenitors to prevent precocious differentiation towards the SMC lineage. In the ureteric epithelium (right) uncommitted progenitors give rise to I-cells which are maintained by RA signaling by preventing precocious differentiation towards the B- and S-cell lineage.

To identify potential target genes of the RAR/RXR transcriptional activator that may account for this function, we next focused on positively regulated transcripts. Using the same parameters as above (intensity > 100, fold change > 1.2 in each experiment), we identified 493 transcripts that decreased upon BMS treatment and 546 genes that were increased in their expression in RA treated ureters yielding a set of 228 genes that are positively regulated by RA signaling (Fig. 6A, Supplementary Tables S10–12). GO enrichment analysis reflected the diversity of roles in which RA signaling plays a role in the organisms but did not provide conclusive evidence about major or causative changes in pathways or factors (Supplementary Table S13).

Among the most deregulated transcripts were the well-established RA target genes *Dhrs3* and *Rarb* (Fig. 6B)^15,28^. To validate the RA-responsive expression of candidate genes and to characterize their spatial confinement we performed RNA *in situ* hybridization analysis on explants of E11.5 kidney and ureter rudiments cultured for 1 day in the presence of 1 µM BMS493 or 1 µM RA (Fig. 6C). As previously shown, *Rarb* expression in the ureteric mesenchyme and renal stroma was strongly RA dependent. To our surprise, some genes including *Tnfsf13b*, *Il33*, *Ecm1* and *Hic1* were expressed in the renal stroma but not in the ureter of control explants. BMS493 treatment abolished expression of these genes, while RA treatment enhanced stromal expression and induced expression in the ureteric mesenchyme. Expression of *Tgm5* and *Shisa3* was strong in the ureteric mesenchyme of control explants. BMS493 abolished, RA treatment slightly enhanced this expression. The weak expression of *Shisa3* in the ureteric epithelium and renal collecting ducts was not RA-responsive. *Tgm2* showed a weak and ubiquitous expression in control explants which was slightly reduced upon BMS493 treatment. Administration of RA augmented the overall expression level of *Tgm2* and induced a strong expression in the ureteric mesenchyme. Expression of *Elf5* was restricted to the epithelium of the nephric duct, the ureter and the collecting ducts and was strongly reduced and enhanced upon BMS493 and RA treatment, respectively.

*Tbx18*, *Tshz3*, *Six1* and *Sox9* that were previously characterized as transcriptional regulators of SMC differentiation^19,29–31^ were not represented in the set of genes positively or negatively regulated by RA signaling. *Bmp4*, the transcriptional target of its signaling activity *Id2*, *Shh* and its transcriptional target *Ptch1* were also unchanged arguing against the fact that RA signaling function is mediated by altered activity of these factors or pathways.

## Discussion

Ureteric SMCs arise in a temporally and spatially tightly controlled manner from the adluminal mesenchymal cell layer. Differentiation starts around E15.5 in the proximal region of the ureter and progresses distally within the following day^2^. The program depends on epithelial signals, SHH and members of WNT family, that activate autocrine BMP4 signaling in the mesenchymal compartment^3,5,6,23,32^. Intriguingly all of these signaling pathways are active from E11.5 onwards but culminate at E14.5 only in transcriptional activation of *Myocd*, which encodes the master regulator of the SMC differentiation program^33^. While it is conceivable that the delayed expression of *Myocd* results from a gradual build-up of positive signaling inputs, an alternative scenario features the presence of inhibitory activities in the undifferentiated ureteric mesenchyme.

Our findings suggest that RA constitutes such an inhibitory signal that acts in the undifferentiated ureteric mesenchyme to temporally and spatially restrict differentiation, possibly through modulation of the WNT signaling pathway. First, a comprehensive expression analysis of components and target genes of the pathway revealed that RA signaling is active in the ureteric mesenchyme from E11.5 to E14.5, i.e. until the onset of SMC differentiation. Second, RA synthesizing enzyme genes *Aldh1a2* and *Aldh1a3* were transiently expressed in the proximal ureter but maintained in its distal aspect in line with the delayed onset of SMC differentiation at this site^2^. An independent function of this distal expression domain for bladder insertion of the distal ureter can, however, not be excluded^11^. Third, our pharmacological loss- and gain-of-function experiments delimited the functional requirement of RA to the interval E11.5 to E14.5. Furthermore, a relative expansion of SMCs at the expense of lamina propria fibrocytes under RA loss-of-function conditions suggests that the mesenchymal progenitors of the ureter prematurely and preferentially differentiated towards the SMC lineage. This hypothesis was further supported by the relative expansion of lamina propria fibrocytes under RA gain-of-function conditions. Importantly, the analysis of differentiation markers revealed a precocious differentiation of SMCs when RA signaling was inhibited.

Microarray analysis characterized the RA-responsive transcriptome in the early ureter. Interestingly, a considerable number of identified genes including *Tgm5*, *Tnfsf13b*, *Il33*, *Elf5* and *Ecm1* were previously reported to be RA-responsive in a similar assay performed for the renal stroma of the embryonic kidney^13^. *Tnfsf13b* and *Il33* activate NFκB signaling and have been reported to control renal branching morphogenesis, while *Ecm1* exerts a similar function via control of GNDF/RET signaling^12,13^. The present study newly identified the RA-responsive expression of two genes encoding antagonists of the WNT signaling pathway in the ureter. *Hic1* encodes for a transcriptional repressor that attenuates canonical WNT signaling by sequestration of TCF-4 and CTNNB1 in specific nuclear compartments^34^. *Shisa3* encodes a member of the SHISA family of proteins that inhibit WNT signaling by retention of WNT receptors in the endoplasmic reticulum and/or by promoting CTNNB1 degradation^35,36^. Importantly, we observed an upregulation of *Axin2* expression, i.e. of canonical WNT signaling in ureter explants treated with BMS493. Thus, a negative modulation of mesenchymal WNT signaling by RA represents a potential molecular mechanism to control or at least partly contribute to the timing of ureteric SMC differentiation.

Since uncommitted mesenchymal progenitors first diversify into adventitial fibrocytes and bipotent progenitors of SMCs and lamina propria fibrocytes, we conclude that RA is specifically required in the bipotent progenitor to prevent precocious differentiation towards the SMC lineage. The late re-expression of *Aldh1a2* in the lamina propria suggests that this cell population constitutes a SMC progenitor in homeostasis and regeneration (Fig. 6C).

Our previous work has shown that I-cells arise in the ureteric epithelium at E13.5 and differentiate into S-cells and B-cells at E15.5 and E16.5, respectively^2^. How this strict temporal sequence of differentiation is molecularly regulated has remained elusive. Studies in the developing and regenerating bladder urothelium reported a function of RA signaling in urothelial specification of endodermal precursors^14^. Given the distinct developmental origin of the ureter urothelium from the intermediate mesoderm, it has remained unclear whether this finding also accounts for the ureter. The present study suggests that RA signaling in the ureter urothelium is involved in the temporal control of cellular differentiation rather than in the specification of progenitor cells. First, pharmacological inhibition of RA signaling in ureter explant cultures between E11.5 and E14.5 resulted in a relative expansion of S-cells at the expense of I-cells. Moreover, compared to control specimens S-cells and B-cells differentiated one day earlier under RA loss-of-function conditions. Second, pharmacological overactivation of RA signaling between E11.5 and E14.5 reduced S-cell differentiation, completely blocked B-cell differentiation and resulted in an expansion of I-cells. Even at later time points was excessive RA sufficient to decrease the abundance of B-cells and to expand I-cells.

A negative effect of RA signaling on epithelial differentiation and especially on the expression of cytokeratins has been reported in the context of keratinocytes, tracheal and bronchial epithelial cells and the salivary gland epithelium^37–39^. *In vitro* studies of the *Krt5* and *Krt14* promotor identified an unconventional mechanism of RA-mediated direct transcriptional repression that involves ligand bound homodimers of RARs^40^. The precocious upregulation of several cytokeratin genes under RA loss-of-function conditions indicates that such a mechanism may be utilized to control the timing of B-cell differentiation in the urothelium. Alternatively, RA may maintain urothelial progenitors indirectly via intermediate factors. Our transcriptome analysis revealed RA-dependent expression of *Elf5* in the undifferentiated ureteric epithelium. The paralogous gene *Elf3* has been reported to activate urothelial differentiation downstream of PPARG signaling^41^. Recent *in vitro* studies identified an enrichment of ELF5 binding sites in regulatory elements of urothelial differentiation genes^42^. Interestingly, misexpression of *Elf5* in the lung epithelium interferes with terminal differentiation^43^. Moreover, *Elf5* regulates stem cell self-renewal and counteracts precocious differentiation in the trophoblast^44,45^. It is conceivable that, in contrast to *Elf3*, *Elf5* negatively regulates the urothelial differentiation program. Our molecular analysis uncovered that RA signaling suppresses the expression of *Dkk1* which encodes a WNT signaling antagonists in the ureteric epithelium^46^. Since WNT signaling is thought to be absent from E12.5 in the ureteric epithelium^23^, the significance of this molecular regulation remains unclear. We conclude that RA signaling acts specifically in urothelial progenitors to prevent precocious differentiation of B- and S-cells and to control progenitor self-renewal and maintenance in development and possibly in homeostasis (Fig. 6D).

## Materials and Methods

### Animals

*Gt(ROSA)26So* ^*tm4(ACTB-tdTomato-EGFP)Luo*^ (synonym: *R26* ^*mTmG*^)^21^ and *Tbx18* ^*tm4(cre)Akis*^ (synonym: *Tbx18* ^*cre*^)^22^ mouse lines were maintained on an NMRI outbred background. Embryos for gene expression and microarray analysis were derived from matings of NMRI wildtype mice. *Tbx18* ^*cre/*+^ *; R26* ^*mTmG*/+^ embryos were obtained from mating *Tbx18* ^*cre/*+^ males with *R26* ^*mTmG/mTmG*^ females. For timed pregnancies, vaginal plugs were checked in the morning after mating, and noon was defined as embryonic day (E) 0.5. Embryos and urogenital systems were dissected in PBS. Specimens were fixed in 4% PFA/PBS, transferred to methanol and stored at −20 °C prior to immunofluorescence or *in situ* hybridization analyses. PCR genotyping was performed on genomic DNA prepared from yolk sac or tail biopsies. All animal work conducted for this study was approved by A. Bleich, state head of the animal facility at Medizinische Hochschule Hannover and performed according to European and German legislation.

### Organ cultures

Cultures of embryonic ureter and kidney explants were performed as described^2^. BMS493 (#3509, Tocris) or RA (#0695, Tocris) were dissolved in DMSO and added to the medium at a final concentration of 1 µM. Culture medium was replaced every day.

### Histological analysis

Embryos, urogenital systems or explant cultures were paraffin-embedded and the proximal ureter was sectioned to 5 µm. Hematoxylin and eosin staining was performed according to standard procedures.

### *In situ* hybridization analysis

Whole-mount *in situ* hybridization was performed following a standard procedure with digoxigenin-labeled antisense riboprobes^47^. Stained specimens were transferred in 80% glycerol prior to documentation. *In situ* hybridization on 10-µm paraffin sections was performed essentially as described^48^. For each marker, at least three independent specimens were analyzed.

### Immunofluorescent detection of antigens

Immunofluorescent analysis on 5-µm paraffin sections was done as described^2^. At least three embryos of each genotype were used for each analysis.

### Cell proliferation and apoptosis assays

Cell proliferation rates in explant cultures (n = 3 per condition) were investigated by the detection of incorporated BrdU on 5 µm paraffin sections similar to published protocols^49^. Explant cultures where incubated for 2 h with 3.3 µg/ml BrdU in the culture medium. For each specimen 12 sections of the proximal ureter were assessed. The BrdU-labeling index was defined as the number of BrdU-positive nuclei relative to the total number of nuclei as detected by 4′,6-diamidino-2-phenylindole (DAPI) counterstaining in arbitrarily defined compartments of the ureter. Data were expressed as mean ± standard deviation. Apoptosis was analyzed on 5 µm paraffin sections using the ApopTag Plus Fluorescein *In Situ* Apoptosis Detection Kit (Chemicon).

### Quantitative reverse transcription PCR

Three independent pools of 10 E12.5 ureters were cultured with DMSO or 1 μM BMS493 for 18 h or 3 days. Total RNA was extracted with peqGOLD RNApure (PeqLab) and first-strand cDNA synthesis was performed with RevertAid reverse transcriptase (Fermentas). For quantitative PCR, the number of cycles was adjusted to the mid-logarithmic phase. The experiments were performed with three biological and two technical replicates. Quantification was performed with ImageJ^50^. Oligos and PCR conditions are provided upon request.

### Statistical analysis

Statistical analysis was performed using the two-tailed Student’s t-test. Data were expressed as mean ± standard deviation. For relative analyses wildtype values were set to 1. Differences were considered significant with a P-value below 0.05 (p < 0.05, *), highly significant (p ≤ 0.005, **) and extremely significant (p ≤ 0.0005, ***).

### Microarray

Three independent pools of 50 E12.5 ureters were cultured with DMSO or 1 μM BMS493 or 1 μM RA for 18 h. Total RNA was extracted with peqGOLD RNApure (PeqLab) and was sent to the Research Core Unit Transcriptomics of Hannover Medical School where RNA was Cy3-labeled and hybridized to Agilent Whole Mouse Genome Oligo v2 (4 × 44 K) Microarrays. To identify differentially expressed genes, normalized expression data was filtered using Excel based on an intensity threshold of 100 and a more than 1.2 fold change in all pools.

### Image documentation

Sections and organ cultures were photographed using a Leica DM5000 microscope with Leica DFC300FX digital camera or a Leica DM6000 microscope with Leica DFC350FX digital camera. Figures were prepared with Adobe Photoshop CS4.

### Data Availability

The datasets generated during the current study are available from the corresponding author on reasonable request.

## Electronic supplementary material

Supplementary Information
