# Supplementary material for: Retinoic acid signaling maintains epithelial and mesenchymal progenitors in the developing mouse ureter
Source: Sci Rep. 2017 Nov 1;7:14803. doi: 10.1038/s41598-017-14790-2 (PMC5665985; doi:10.1038/s41598-017-14790-2)
Supplement: Supplementary file 1 — Supplementary Information [file 41598_2017_14790_MOESM1_ESM.pdf]

Supplementary Information

Retinoic acid signaling maintains epithelial and mesenchymal progenitors in the developing mouse ureter

Tobias Bohnenpoll, Anna-Carina Weiss, Maurice Labuhn, Timo H. Lüdtke, M.-O. Trowe and Andreas Kispert

Institut für Molekularbiologie, Medizinische Hochschule Hannover, 30625 Hannover, Germany

Supplementary Figures

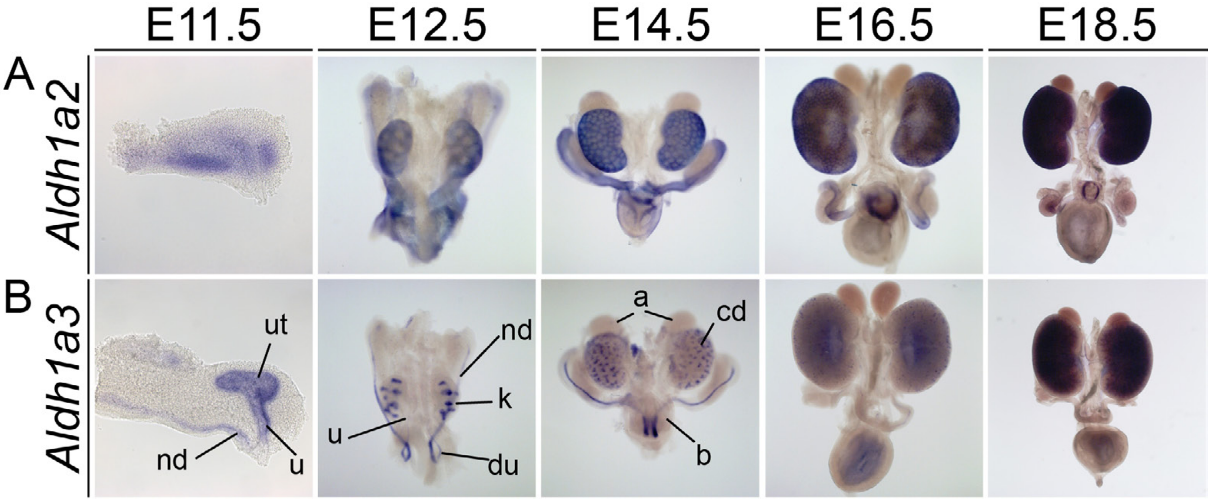

Supplementary Figure S1. Expression of RA producing enzymes in urogenital system development. (A,B) Expression analysis by whole mount *in situ* hybridization of *Aldh1a2* (A) and *Aldh1a3* (B) on E11.5 kidney rudiments and on E12.5, E14.5, E16.5 and E18.5 whole urogenital systems. (A) *Aldh1a2* is expressed throughout the ureteric mesenchyme at E11.5. From E12.5 onwards, expression is confined to the mesenchyme surrounding the distal ureter and the bladder. Additional expression domains comprise the kidney and the mesenchyme surrounding the nephric duct. (B) *Aldh1a3* is found in the epithelium of the nephric duct and the ureteric bud at E11.5. At subsequent stages, expression is confined to the distal ureter epithelium, the renal collecting duct system and the nephric duct epithelium. a, adrenal; b, bladder; cd, collecting ducts; du, distal ureter; k, kidney; nd, nephric duct; u, ureter; ureteric tip.

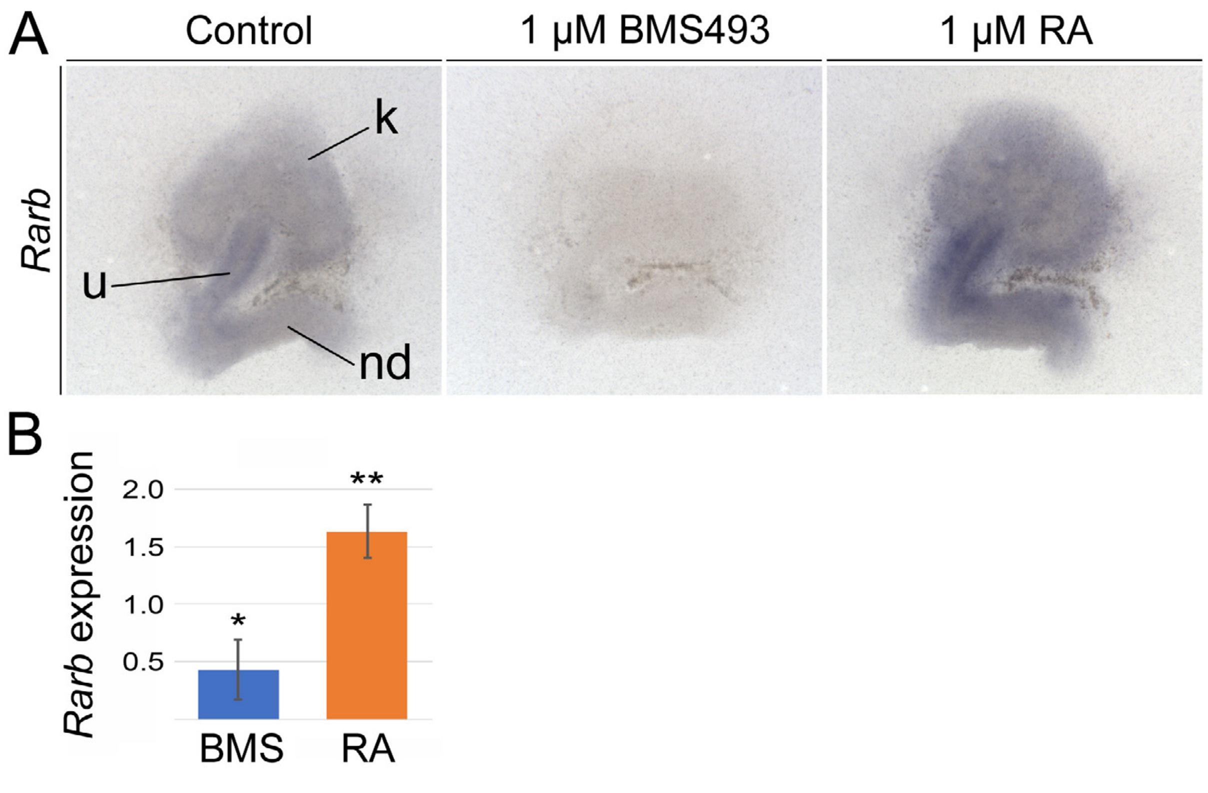

Supplementary Figure S2. Pharmacological manipulation of RA signaling in kidney/ureter explant cultures. (A) E11.5 kidney rudiments were cultured for 18 h in the presence of DMSO, 1 µ M BMS493 or 1 µ M RA, and were then subjected to *in situ* hybridization analysis for expression of the target gene of RA signaling, *Rarb*. In the control, *Rarb* shows strong expression in the ureteric mesenchyme and weak expression in the renal stroma. Upon BMS493 treatment, *Rarb* expression is lost in the entire explant, upon RA treatment expression is enhanced. (B) Quantification of changes of *Rarb* expression by RT-PCR analysis on pools of 10 ureters explanted at E11.5 and cultured for 18 h in the presence of DMSO, 1 µM BMS493 or 1 µM RA. Expression in BMS493 treated cultures is significantly reduced to 0.43±0.23, p=0.01, RA treatment highly significantly increases *Rarb* expression to 1.49±0.28, p=0.004. RT-PCRs were performed on three biological and two technical replicates. DMSO treated wildtype cultures used as reference were set to 1. Together, these results confirm the suitability of the chosen conditions to manipulate RA signaling in ureter cultures. k, kidney; nd, nephric duct; u, ureter.

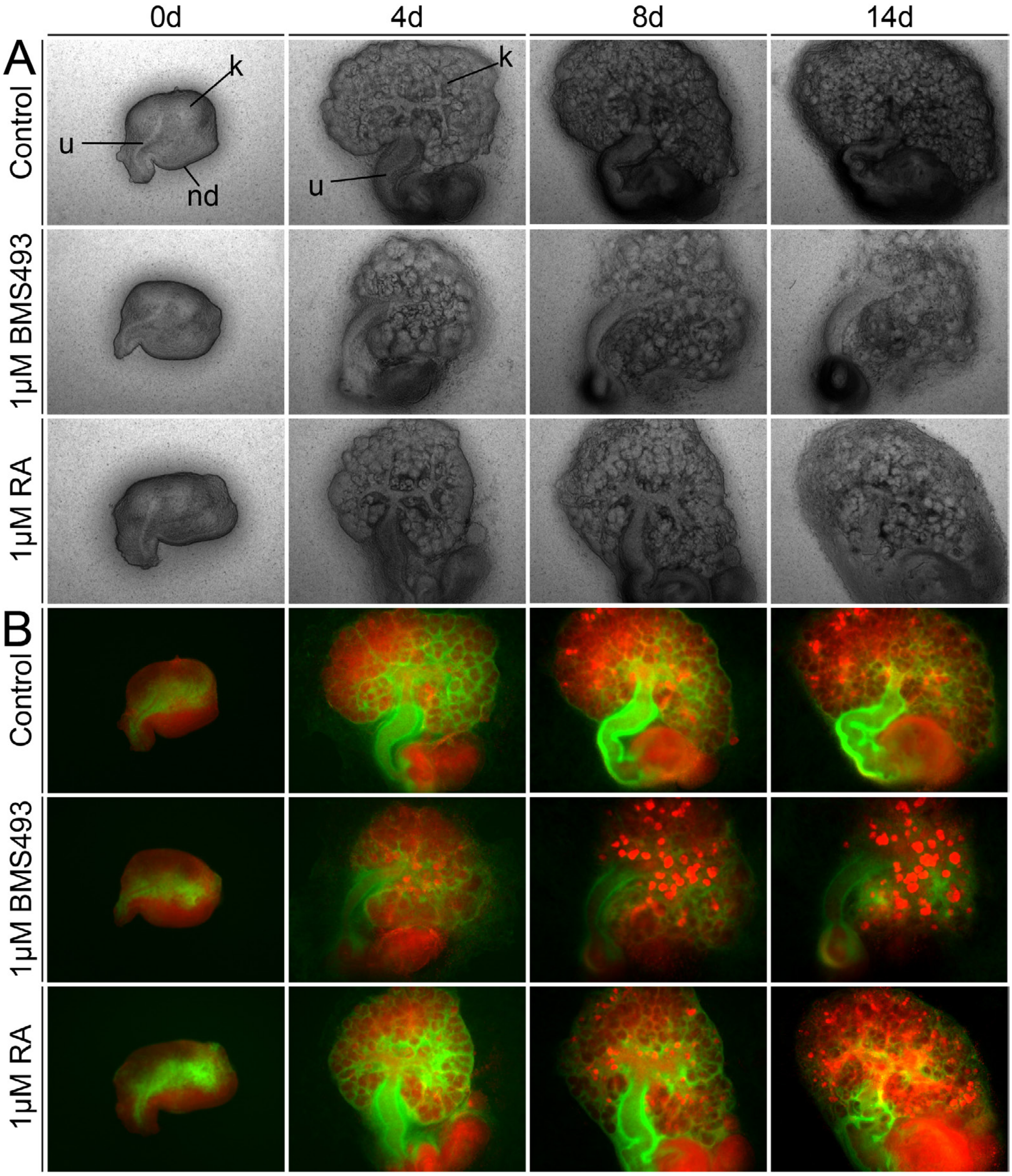

Supplementary Figure S3. Pharmacological manipulation of RA signaling in E11.5 kidney explant cultures. (A,B) E11.5 *Tbx18*$^{cre/+}$;*R26*$^{mTmG/+}$ kidney rudiments were explanted and cultured for 14 d in the presence of DMSO, 1 µM BMS493 or 1 µM RA. Brightfield (A) and GFP/RFP epifluorescence images (B) are displayed to visualize explant growth after 0 d, 4 d, 8 d and 14 d of culture under given conditions. GFP expression marks the ureteric mesenchyme and a sub-population of the renal stroma. Note that GFP expression appears reduced in the ureteric mesenchyme in explants cultured for 14 days with 1 µM BMS493. k, kidney; nd, nephric duct; u, ureter.

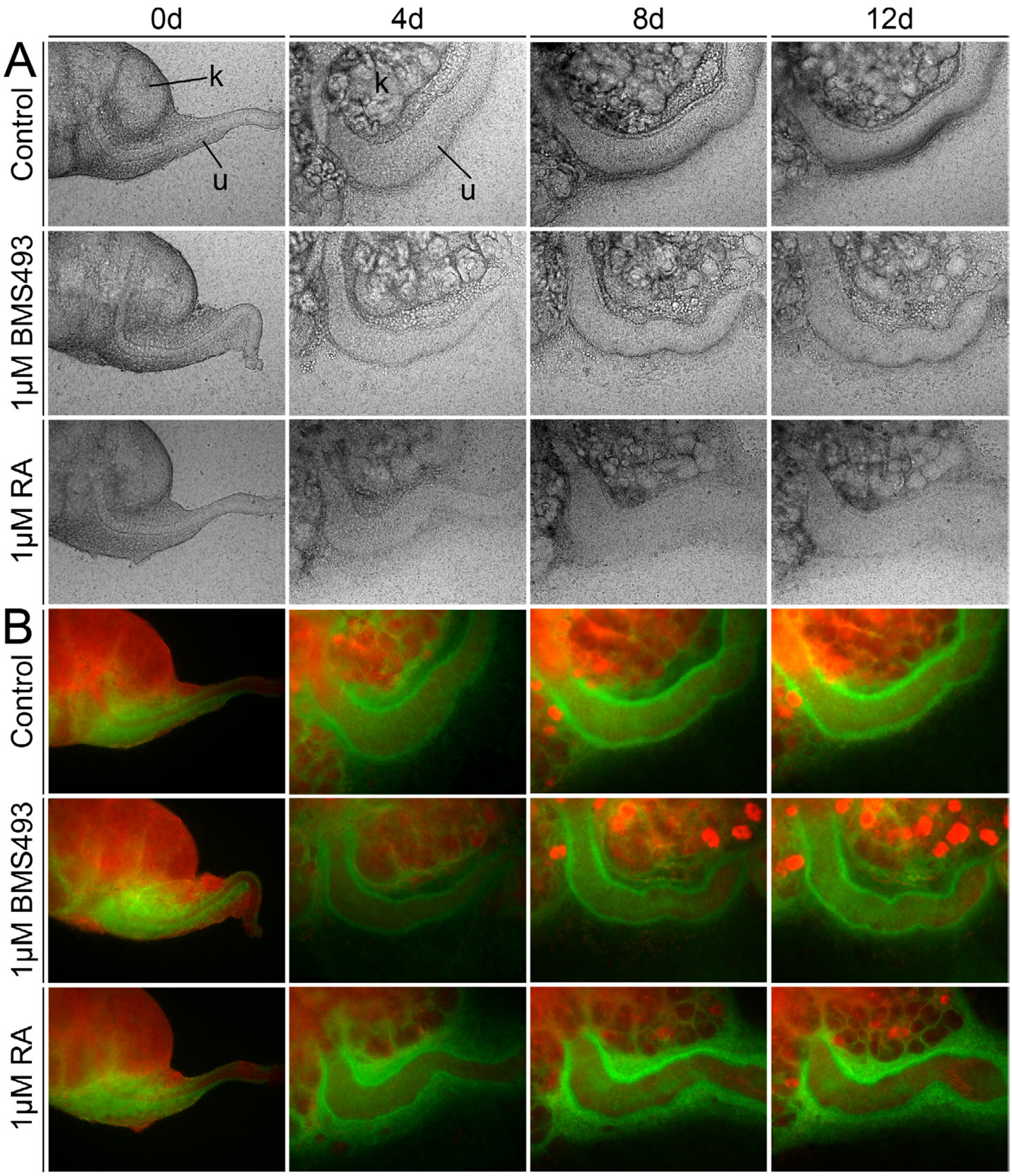

Supplementary Figure S4. Pharmacological manipulation of RA signaling in E12.5 kidney explant cultures. (A,B) E12.5 *Tbx18*$^{cre/+}$;*R26*$^{mTmG/+}$ kidney rudiments were explanted and cultured for 12 d in the presence of DMSO, 1 µM BMS493 or 1 µM RA. Brightfield (A) and GFP/RFP epifluorescence images (B) are displayed to visualize explant growth after 0 d, 4 d, 8 d and 12 d of culture under given conditions. GFP expression marks the ureteric mesenchyme and a sub-population of the renal stroma. Note its apparent reduction in the ureteric mesenchyme in explants cultured for 12 days with 1 µM BMS493. k, kidney; u, ureter.

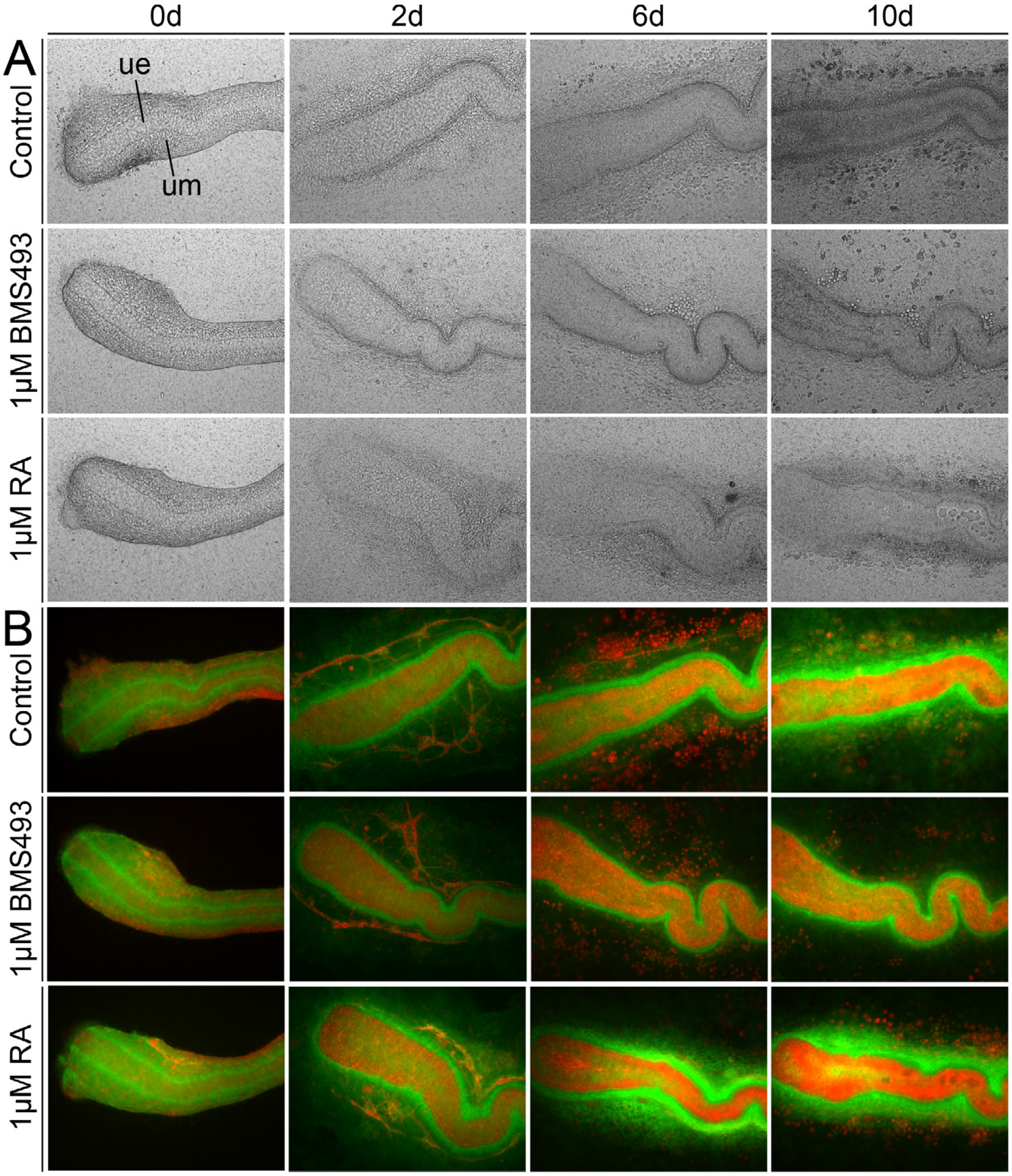

Supplementary Figure S5. Pharmacological manipulation of RA signaling in E14.5 ureter explant cultures. (A,B) E14.5 *Tbx18*$^{cre/+}$;*R26*$^{mTmG/+}$ ureters were explanted and cultured for 10 d in the presence of DMSO, 1 µM BMS493 or 1 µM RA. Brightfield (A) and GFP/RFP epifluorescence images (B) are displayed to visualize explant growth after 0 d, 2 d, 6 d and 10 d of culture under the given conditions. GFP expression marks the ureteric mesenchyme, RFP expression the ureteric epithelium and attached fibrocytes in the periphery of the ureter. GFP expression appears strongly reduced in the ureteric mesenchyme in explants cultured for 10 days with 1 µ M BMS493, and increased when cultures are grown in 1 µM RA. ue, ureteric epithelium; um, ureteric mesenchyme.

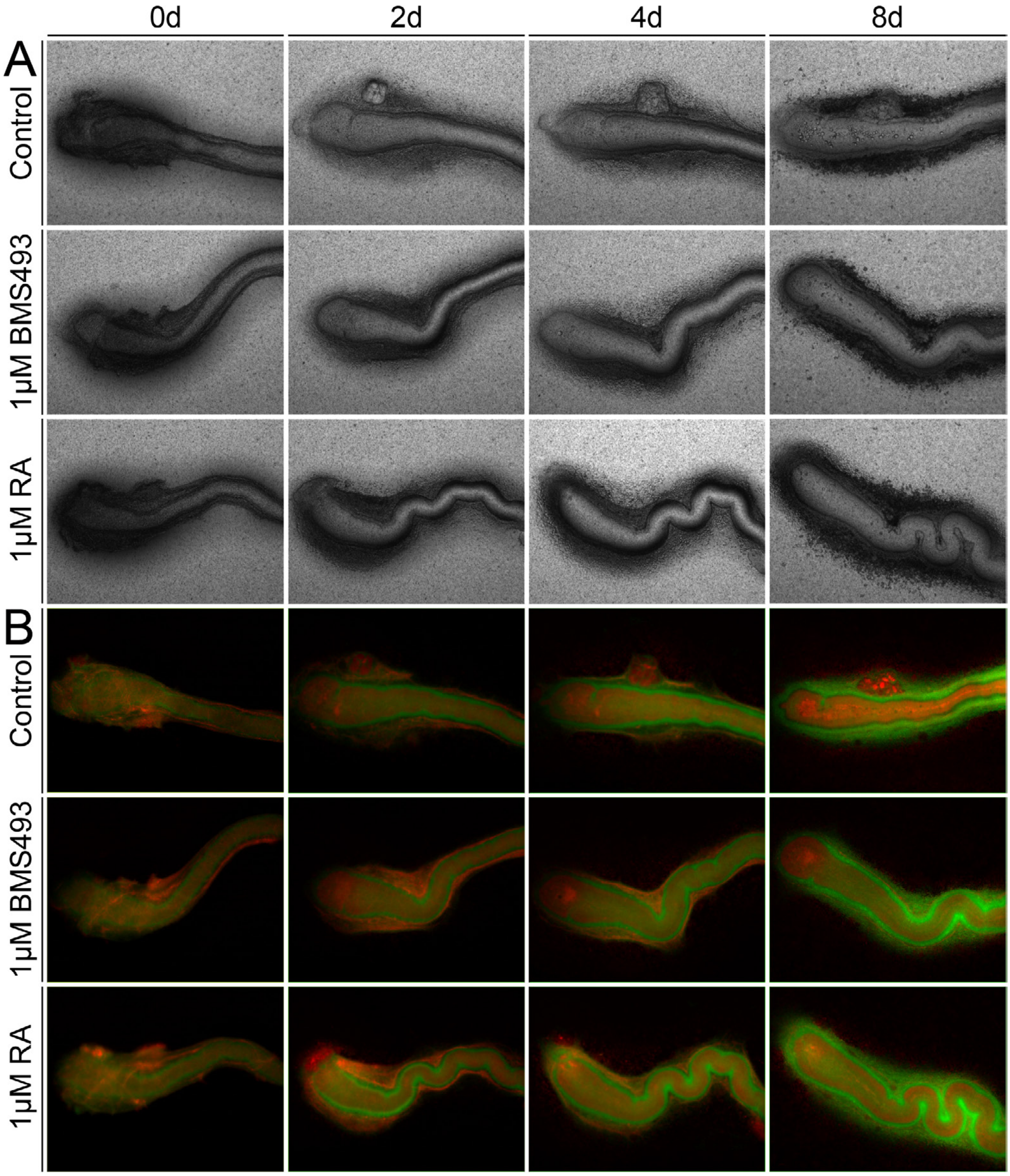

Supplementary Figure S6. Pharmacological manipulation of RA signaling in E16.5 ureter explant cultures. (A,B) E16.5 *Tbx18*$^{cre/+}$;*R26*$^{mTmG/+}$ ureters were explanted and cultured for 8 d in the presence of DMSO, 1 µM BMS493 or 1 µM RA. Brightfield (A) and GFP/RFP epifluorescence images (B) are displayed to visualize explant growth after 0 d, 2 d, 4 d and 8 d of culture under given conditions. GFP expression marks the ureteric mesenchyme, RFP expression the ureteric epithelium.

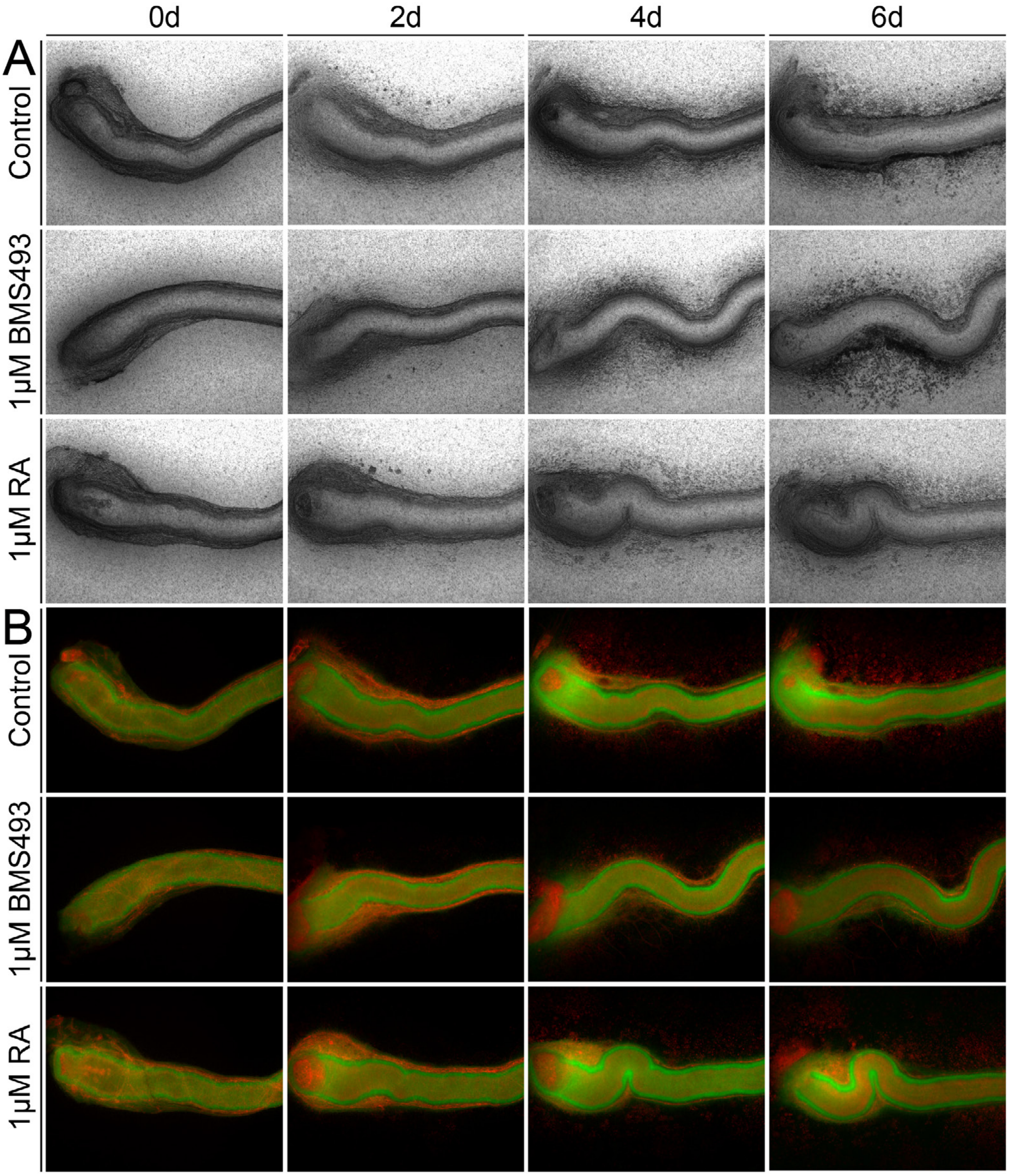

Supplementary Figure S7. Pharmacological manipulation of RA signaling in E18.5 ureter explant cultures. (A,B) E18.5 *Tbx18*$^{cre/+}$*;R26*$^{mTmG/+}$ ureters were explanted and cultured for 6 d in the presence of DMSO, 1 µM BMS493 or 1 µM RA. Brightfield (A) and GFP/RFP epifluorescence images (B) are displayed to visualize explant growth after 0 d, 2 d, 4 d and 6 d of culture under given conditions. GFP expression marks the ureteric mesenchyme.

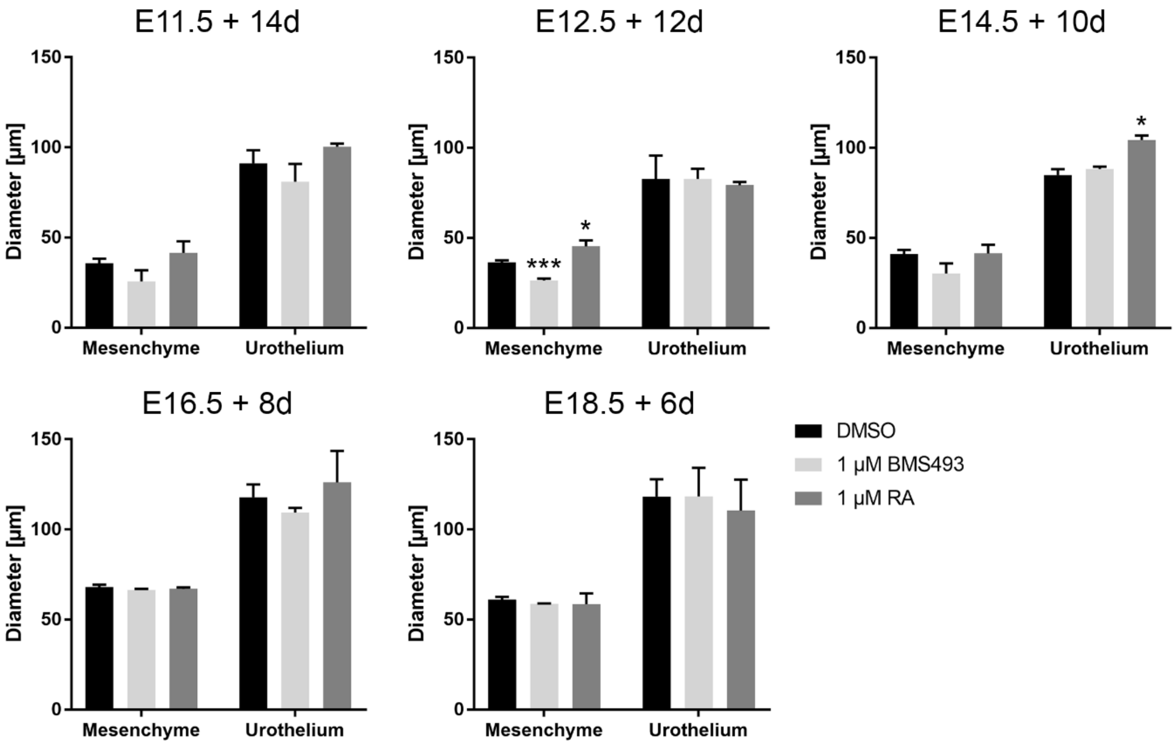

Supplementary Figure S8. Manipulation of RA signaling levels marginally affects the thickness of the ureteric tissue compartments. Mesenchymal and urothelial diameters were quantified on transverse proximal sections of E11.5 + 14d, E12.5 + 12d, E14.5 + 10d, E16.5 + 8d and E18.5 + 6d wildtype ureter explants that were treated with DMSO, 1 µM BMS493 or 1 µM RA. To adjust for the flattened morphology of ureter explant cultures, diameters were measured in parallel and perpendicular to the explant surface and the average was calculated. For each condition 3 individual specimens with 6 sections were analyzed. Data were expressed µm as mean ± standard deviation. E11.5 + 14d: Mesenchyme: 35.7 ± 2.6 (DMSO) vs. 25.6 ± 6.3 (BMS493) vs. 41.5 ± 6.3 (RA); Urothelium: 91.2 ± 7.2 (DMSO) vs. 80.9 ± 10.0 (BMS493) vs. 100.4 ± 1.7 (RA). E12.5 + 12d: Mesenchyme: 36.4 ± 1.2 (DMSO) vs. 26.4 ± 1.2 (BMS493, p=9.6E-04) vs. 45.3 ± 3.4 (RA, p=2.4E-02); Urothelium: 82.7 ± 12.9 (DMSO) vs. 82.8 ± 5.6 (BMS493) vs. 79.2 ± 1.8 (RA). E14.5 + 10d: Mesenchyme: 41.1 ± 2.3 (DMSO) vs. 30.3 ± 5.8 (BMS493) vs. 41.5 ± 4.8 (RA); Urothelium: 84.9 ± 3.3 (DMSO) vs. 88.3 ± 1.2 (BMS493) vs. 104.4 ± 2.5 (RA, p=2.6E-03). E16.5 + 8d: Mesenchyme: 68.0 ± 1.3 (DMSO) vs. 66.4 ± 0.6 (BMS493) vs. 67.0 ± 0.9 (RA); Urothelium: 117.8 ± 7.2 (DMSO) vs. 109.4 ± 2.6 (BMS493) vs. 126.1 ± 17.5 (RA). E18.5 + 8d: Mesenchyme: 61.0 ± 1.6 (DMSO) vs. 58.7 ± 0.2 (BMS493) vs. 58.6 ± 5.9 (RA); Urothelium: 118.1 ± 9.7 (DMSO) vs. 118.3 ± 15.9 (BMS493) vs. 110.4 ± 17.2 (RA). The two-tailed Student's t-test was used to test for significance. * p≤0.05; ** p≤0.001; *** p≤0.0001.

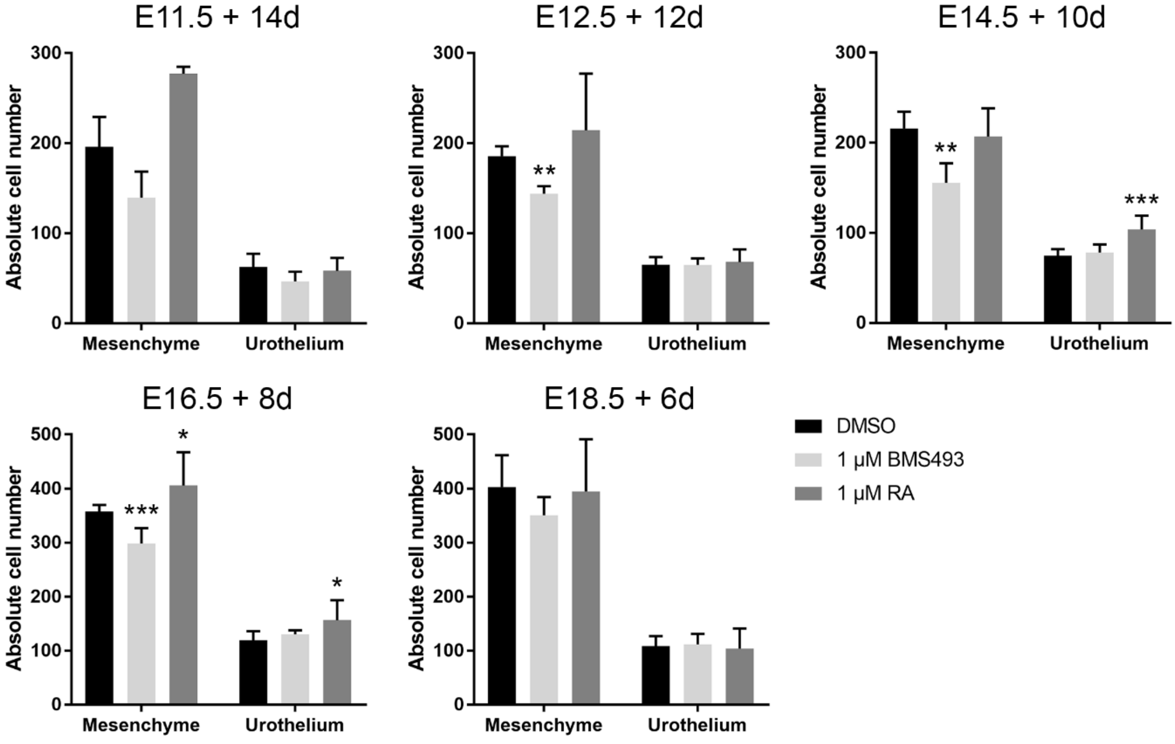

Supplementary Figure S9. Manipulation of RA signaling levels affects the cellularity of the ureteric tissue compartments. Absolute mesenchymal and urothelial cell numbers were quantified on transverse proximal sections of E11.5 + 14d, E12.5 + 12d, E14.5 + 10d, E16.5 + 8d and E18.5 + 6d wildtype ureter explants that were treated with DMSO, 1 μM BMS493 or 1 μM RA. For each condition 3 individual specimens with 6 sections were analyzed. Data were expressed as mean ± standard deviation. E11.5 + 14d: Mesenchyme: 196.0 ± 33.2 (DMSO) vs. 139.5 ± 29.0 (BMS493) vs. 277.0 ± 7.8 (RA, p=1.7E-02); Urothelium: 62.5 ± 14.7 (DMSO) vs. 46.5 ± 10.7 (BMS493) vs. 58.5 ± 14.5 (RA). E12.5 + 12d: Mesenchyme: 185.5 ± 11.1 (DMSO) vs. 143.8 ± 8.5 (BMS493, p=2.3E-04) vs. 214.4 ± 62.9 (RA); Urothelium: 65.2 ± 8.5 (DMSO) vs. 64.8 ± 7.4 (BMS493) vs. 68.0 ± 14.1 (RA). E14.5 + 10d: Mesenchyme: 215.8 ± 18.7 (DMSO) vs. 155.4 ± 21.9 (BMS493, p=1.6E-03) vs. 207.0 ± 31.4 (RA); Urothelium: 74.8 ± 7.0 (DMSO) vs. 78.3 ± 8.9 (BMS493) vs. 103.8 ± 15.1 (RA, p=8.4E-05). E16.5 + 8d: Mesenchyme: 357.4 ± 12.3 (DMSO) vs. 298.3 ± 28.2 (BMS493, p=2.9E-05) vs. 405.7 ± 61.4 (RA3.4E-02); Urothelium: 119.0 ± 17.1 (DMSO) vs. 130.2 ± 7.4 (BMS493) vs. 156.3 ± 36.9 (RA, p=1.4E-02). E18.5 + 8d: Mesenchyme: 402.5 ± 59.4 (DMSO) vs. 350.3 ± 34.1 (BMS493) vs. 394.5 ± 96.7 (RA); Urothelium: 108.5 ± 18.6 (DMSO) vs. 111.5 ± 19.8 (BMS493) vs. 103.7 ± 37.5 (RA). The two-tailed Student's t-test was used to test for significance. * p≤0.05; ** p≤0.001; *** p≤0.0001.

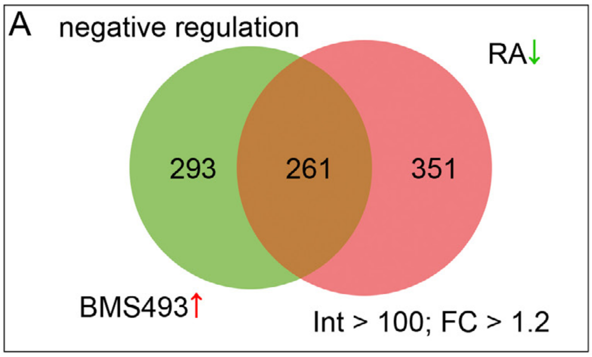

B

Rank	Gene symbol	BMS493 FC	RA FC
1	*S100a4*	4.2	-3.8
2	*Krt6a*	3.4	-3.6
3	*Rgcc*	3.3	-4.2
4	*Serpine1*	2.9	-4.2
5	*Ugt2b34*	2.7	-4.7
6	*Dkk1*	2.7	-4.1
7	*Kcnc2*	2.7	-2.2
8	*Osr2*	2.7	-1.9
9	*Ryr3*	2.6	-1.9
10	*Wif1*	2.5	-2.4
11	*Serpina3f*	2.5	-2.8
12	*Penk*	2.5	-3.0
13	*Glis1*	2.5	-1.9
14	*Aard*	2.4	-2.3
15	*Serpina3i*	2.3	-2.8
16	*Lypd3*	2.3	-1.8
17	*Slc26a7*	2.3	-2.1
18	*Grm7*	2.3	-4.1
19	*Trim29*	2.2	-2.2
20	*Nts*	2.2	-5.6
21	*Gabra1*	2.2	-5.7
22	*Npy*	2.2	-5.2
23	*Vcam1*	2.1	-3.0
24	*Krt5*	2.1	-2.5
25	*Rasd2*	2.1	-2.7
87	*Actg2*	1.6	-2.2
145	*Acta1*	1.5	-1.8
174	*Actc1*	1.4	-2.2
229	*Tagln*	1.3	-2.0

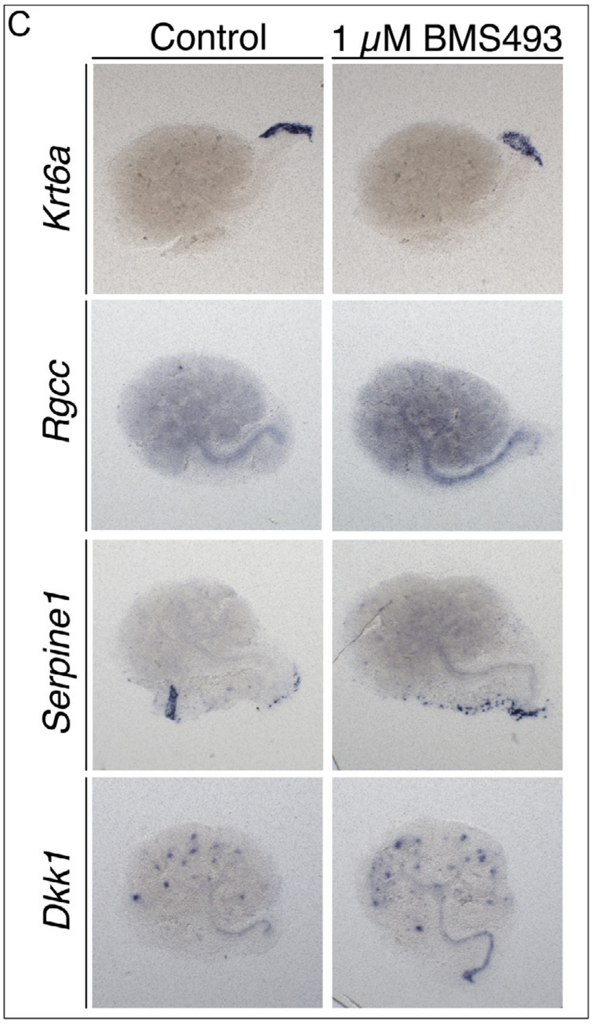

Supplementary Figure S10. Identification of genes negatively regulated by RA signaling in microarray experiments. (A) Summary of the results from the microarray analysis of E12.5 ureters explanted and treated with DMSO or 1 μM BMS493 or 1 μM RA for 18 h filtered with an intensity (Int) threshold of 100 and a fold change (FC) cut-off of 1.2. The Venn diagram displays transcripts that were negatively regulated by RA signaling, i.e. up-regulated upon BMS493 treatment (554, green) or down-regulated upon RA treatment (612, red). The intersection shows the common group of negatively regulated transcripts (261). (B) Top 25 of the common negatively regulated genes were ranked according to their FC up-regulation upon BMS493 treatment. (C) *In situ* hybridization analysis of expression of selected genes (*Krt6a, Rgcc*, *Serpine1*, *Dkk1*) which were negatively regulated by RA signaling on E12.5 kidney explant cultures, treated with 1 μM BMS493 for 1 day.

Supplementary Tables

Supplementary Table S1. Ratios of mesenchymal cell types under RA signaling loss- and gain-of-function conditions. Values are displayed in % as mean ± sd of adventitial cells (A-cells), smooth muscle cells (SM-cells) and lamina propria cells (LP-cells) with respect to the total cell number. Quantification is based on 6 sections of the proximal ureter from 3 individuals of each stage and condition. Student's t-test was applied to test for significant differences between control (DMSO) and test groups (BMS493 or RA). P-values are shown in brackets when $p \leq 0.01$.

**E11.5 + 14d**	**A-cells (%)**	**SM-cells (%)**	**LP-cells (%)**
DMSO	31.6 ± 4.5	44.6 ± 2.6	23.8 ± 3.2
1 µM BMS493	42.4 ± 4.7	53.7 ± 2.6 (p=2.4E-03)	3.9 ± 2.5 (p=6.2E-05)
1 µM RA	37.4 ± 0.6	35.5 ± 2.4 (p=5.4E-03)	27.1 ± 2.4
**E12.5 + 12d**	**A-cells (%)**	**SM-cells (%)**	**LP-cells (%)**
DMSO	43.5 ± 11.8	40.8 ± 11.7	15.7 ± 2.2
1 µM BMS493	38.1 ± 2.3	56.2 ± 2.1 (p=3.6E-06)	5.7 ± 2.2 (6.1E-05)
1 µM RA	49.5 ± 4.1	30.2 ± 5.2 (p=2.2E-03)	20.3 ± 2.0 (1.0E-02)
**E14.5 + 10d**	**A-cells (%)**	**SM-cells (%)**	**LP-cells (%)**
DMSO	25.6 ± 3.5	57.9 ± 4.3	16.5 ± 1.1
1 µM BMS493	19.3 ± 2.1 (p=9.2E-03)	70.1 ± 3.0 (p=8.5E-04)	10.6 ± 3.6 (p=7.2E-03)
1 µM RA	28.5 ± 2.8	54.0 ± 5.7	17.5 ± 4.4
**E16.5 + 8d**	**A-cells (%)**	**SM-cells (%)**	**LP-cells (%)**
DMSO	25.7 ± 2.5	59.5 ± 2.9	14.8 ± 0.8
1 µM BMS493	24.4 ± 2.1	60.5 ± 2.3	15.0 ± 1.6
1 µM RA	24.5 ± 2.1	60.7 ± 2.3	14.8 ± 1.6
**E18.5 + 6d**	**A-cells (%)**	**SM-cells (%)**	**LP-cells (%)**
DMSO	35.6 ± 2.2	53.0 ± 3.0	11.4 ± 1.3
1 µM BMS493	33.7 ± 3.2	54.0 ± 2.5	12.3 ± 2.9
1 µM RA	35.2 ± 1.4	53.2 ± 1.1	11.6 ± 1.5

Supplementary Table S2. Ratios of urothelial cell types under RA signaling loss- and gain-of-function conditions. Values are displayed in % as mean ± sd of basal cells (B-cells), intermediate cells (I-cells) and superficial cells (S-cells) with respect to the total cell number. Quantification is based on 6 sections of the proximal ureter from 3 individuals of each stage and condition. Student's t-test was applied to test for significant differences between control (DMSO) and test groups (BMS493 or RA). P-values are shown in brackets when p ≤ 0.01.

**E11.5 + 14d**	**B-cells (%)**	**I-cells (%)**	**S-cells (%)**
DMSO	35.0 ± 11.8	62.6 ± 11.7	2.4 ± 2.2
1 µM BMS493	42.0 ± 7.0	31.5 ± 10.7 (p=7.2E-04)	26.5 ± 7.4 (p=1.8E-05)
1 µM RA	2.0 ± 1.8 (p=4.8E-05)	95.0 ± 10.9 (p=4.9E-05)	3.0 ± 1.2
**E12.5 + 12d**	**B-cells (%)**	**I-cells (%)**	**S-cells (%)**
DMSO	44.7 ± 2.3	49.7 ± 2.3	5.6 ± 1.5
1 µM BMS493	45.3 ± 4.1	25.4 ± 9.3 (p=2.3E-04)	29.4 ± 8.2 (p=9.9E-05)
1 µM RA	6.9 ± 1.9 (p=3.5E-10)	85.9 ± 7.1 (p=8.3E-07)	7.1 ± 5.5
**E14.5 + 10d**	**B-cells (%)**	**I-cells (%)**	**S-cells (%)**
DMSO	24.3 ± 5.2	71.5 ± 7.2	4.2 ± 3.4
1 µM BMS493	22.9 ± 7.9	50.4 ± 4.9 (p=1.9E-06)	26.7 ± 5.3 (p=1.2E-08)
1 µM RA	3.4 ± 2.9 (p=1.3E-08)	89.2 ± 4.3 (p=1.1E-05)	7.4 ± 3.3
**E16.5 + 8d**	**B-cells (%)**	**I-cells (%)**	**S-cells (%)**
DMSO	53.1 ± 7.5	44.1 ± 7.0	2.8 ± 1.1
1 µM BMS493	52.3 ± 6.9	45.3 ± 7.0	2.3 ± 0.7
1 µM RA	32.8 ± 6.8 (p=1.8E-05)	65.3 ± 6.7 (p=6.3E-06)	1.9 ± 0.6
**E18.5 + 6d**	**B-cells (%)**	**I-cells (%)**	**S-cells (%)**
DMSO	80.4 ± 3.9	14.6 ± 3.8	5.0 ± 1.3
1 µM BMS493	84.5 ± 4.4	9.9 ± 2.8	5.6 ± 2.2
1 µM RA	71.3 ± 2.6 (p=8.4E-04)	22.9 ± 3.0 (p=2.0E-02)	5.8 ± 1.2

Supplementary Table S3. Proliferation rates in the developing ureter under RA signaling loss- and gain-of-function conditions. Shown are BrdU indices for the inner mesenchymal (IM) and outer mesenchymal compartment (OM) and the ureteric epithelium (E) as mean ± sd. Quantification is based on 12 sections of the proximal ureter from 3 individuals of each stage and condition. Student's t-test was applied to test for significant differences between control (DMSO) and test groups (BMS493 or RA). ** p=3.350E-05.

**E11.5 + 1d**	**IM**	**OM**	**E**
DMSO	0.266 ± 0.045	0.073 ± 0.022	0.006 ± 0.021
1 μM BMS493	0.159 ± 0.055 **	0.081 ± 0.033	0.042 ± 0.04
1 μM RA	0.261 ± 0.082	0.077 ± 0.021	0.032 ± 0.038
**E12.5 + 1d**	**IM**	**OM**	**E**
DMSO	0.293 ± 0.034	0.138 ± 0.023	0.234 ± 0.148
1 μM BMS493	0.265 ± 0.063	0.136 ± 0.017	0.166 ± 0.068
1 μM RA	0.261 ± 0.069	0.137 ± 0.018	0.2 ± 0.141
**E14.5 + 1d**	**IM**	**OM**	**E**
DMSO	0.263 ± 0.061	0.155 ± 0.020	0.336 ± 0.024
1 μM BMS493	0.218 ± 0.035	0.14 ± 0.012	0.321 ± 0.073
1 μM RA	0.258 ± 0.046	0.169 ± 0.037	0.374 ± 0.052

Supplementary Table S4. Relative expression of differentiation markers quantified by RT-PCR analysis of BMS493 treated ureter explants. Shown are the relative expression values of the SMC markers *Tagln* and *Acta2*, the S-cell marker *Upk1b* and the B-cell marker *Krt5* in ureters that were treated for 18 h and 3 days with DMSO or the RA signaling inhibitor BMS493. Values are displayed as mean ± sd. The data are derived from RT-PCR experiments with three biological (pools of 10 ureters each) and two technical replicates. Student's t-test was applied to test for significant differences between DMSO and BMS493 treated groups.

	E12.5 +18h			E12.5 + 3d		
	DMSO	1 µM BMS493	p-value	DMSO	1 µM BMS493	p-value
Tagln	1 ± 0.32	1.27 ± 0.46	0.2801	1 ± 0.35	1.19 ± 0.35	0.6518
Acta2	1 ± 0.36	1.20 ± 0.41	0.3896	1 ± 0.24	1.01 ± 0.15	0.9290
Upk1b	1 ± 0.32	0.69 ± 0.22	0.0841	1 ± 0.35	1.32 ± 0.20	0.2084
Krt5	1 ± 0.73	2.35 ± 0.87	0.0162	1 ± 0.86	6.51 ± 2.58	0.0007

Supplementary Table S5. Relative expression of target genes of SHH, WNT and BMP signaling quantified by RT-PCR analysis of BMS493 or RA treated ureter explants. Shown are the relative expression values of the SHH target *Ptch1*, the WNT target *Axin2* and the BMP target *Id2* in explants of E11.5 ureters that were treated for 18 h with DMSO, with 1 μM of the RA signaling inhibitor BMS493 or with 1 μM RA. Values are displayed as mean ± sd. The data are derived from RT-PCR experiments with three biological (pools of 10 ureters each) and two technical replicates. Student's t-test was applied to test for significant differences between DMSO and BMS493 treated groups.

	E12.5 +18h				
	DMSO	1 μM BMS493	p-value	1 μM RA	p-value
Ptch1	1 ± 0.19	1.03 ± 0.18	0.8108	0.85 ± 0.23	0.41
Axin2	1 ± 0.60	1.94 ± 0.52	0.0158	0.82 ± 0.14	0.26
Id2	1 ± 0.14	0.88 ± 0.11	0.1141	0.93 ± 0.12	0.59

Supplementary Table S6. Transcripts identified by microarray analysis that were upregulated upon BMS493 treatment in the early ureter. Shown is a list of transcripts that were upregulated after BMS493 treatment of E12.5 ureter explants. Three groups for each condition were compared to untreated controls and the resulting fold changes (FC) in expression are displayed. Intensity thresholds were >100; fold changes were larger than 1.2.

Gene symbol	FC 1	FC 2	FC 3	Avg FC
BC100530	12.9	4.3	2.2	6.5
3830417A13Rik	3.8	4.2	4.7	4.2
S100a4	4.0	4.5	4.0	4.2
Krt6b	4.4	5.0	2.6	4.0
Col17a1	3.1	4.7	2.9	3.6
Krt6a	4.0	4.2	2.1	3.4
Rgcc	3.1	3.5	3.3	3.3
Krt14	3.3	3.7	2.3	3.1
Irx3	2.6	3.0	3.3	3.0
Serpine1	3.3	2.9	2.6	2.9
Ugt2b34	2.3	2.7	3.2	2.7
Gm5476	2.4	3.5	2.1	2.7
Dkk1	2.3	2.6	3.1	2.7
Kcnc2	2.5	2.9	2.5	2.7
Osr2	3.4	2.6	2.0	2.7
Ryr3	2.8	2.5	2.5	2.6
Adcyap1	3.3	2.5	2.0	2.6
Mc4r	3.0	2.3	2.2	2.5
Wif1	2.5	2.8	2.3	2.5
Serpina3f	2.2	2.8	2.6	2.5
Pcp4l1	2.3	2.4	2.8	2.5
ENSMUST0000017796	1.8	1.2	4.5	2.5
Penk	2.4	2.2	2.9	2.5
Glis1	2.2	2.4	2.8	2.5
Rasgrf1	2.4	2.8	2.0	2.4
Cbr2	2.8	2.4	2.0	2.4
Aard	2.7	2.1	2.3	2.4
Serpina3i	2.4	2.7	1.9	2.3
Higd1c	2.9	2.6	1.4	2.3
Lypd3	2.6	2.3	2.0	2.3
Gm10639	1.8	2.3	2.8	2.3
Slc26a7	2.4	1.9	2.5	2.3
Grm7	2.0	2.7	2.1	2.3
Trim29	2.8	2.2	1.7	2.2
Nts	2.0	2.8	1.8	2.2
Mettl7a2Higd1c	2.3	2.4	1.9	2.2
Pde4d	2.0	2.0	2.6	2.2
Vgf	1.9	2.9	1.8	2.2
Gabra1	2.1	1.9	2.5	2.2
Npy	2.9	2.0	1.6	2.2
Cxcl5	1.9	2.4	2.2	2.2
C1qtnf7	2.2	2.3	2.0	2.2
Krt17	2.3	2.7	1.3	2.1
Vcam1	2.1	2.2	2.0	2.1
Krt5	2.4	2.2	1.7	2.1
ENSMUST0000009904	1.8	1.2	3.2	2.1
ENSMUST0000009905	1.9	1.2	3.1	2.1
Rasl10a	2.1	2.1	2.0	2.1
Dmkn	1.8	2.1	2.3	2.1
Rasd2	1.9	2.6	1.7	2.1
Cd55	1.8	2.0	2.4	2.1
Abi3bp	1.7	2.1	2.4	2.1
Gem	2.0	2.1	2.0	2.0
Kcnab1	2.0	1.9	2.1	2.0

Gene symbol	FC 1	FC 2	FC 3	Avg FC
Ltbp2	2.0	2.3	1.8	2.0
Emp2	1.5	2.8	1.8	2.0
Mt2	1.9	2.0	2.1	2.0
Artn	1.9	2.1	2.0	2.0
Pgam2	2.0	1.9	2.0	2.0
Guca2a	2.2	2.2	1.5	2.0
Dmrta2	1.7	2.0	2.2	2.0
Krt16	2.0	1.6	2.4	2.0
Daf2	2.0	1.9	2.0	2.0
Anxa8	2.1	2.1	1.7	2.0
Svep1	1.8	2.1	1.8	1.9
Kcnip4	2.0	1.9	1.9	1.9
Hmcn1	2.1	2.0	1.7	1.9
Isl2	1.7	1.7	2.4	1.9
Ifi202b	2.0	2.2	1.5	1.9
Tox	1.9	1.9	1.9	1.9
Tcea3	1.9	2.3	1.5	1.9
S100a6	2.1	2.0	1.5	1.9
Tfap2a	1.8	2.6	1.3	1.9
Rasgef1a	1.4	2.2	2.0	1.9
Slc16a5	1.6	2.1	1.9	1.9
Pcdh10	2.2	1.9	1.6	1.9
Nfatc2	1.6	2.1	1.9	1.9
Cxcl14	2.0	1.8	1.8	1.9
Cx3cl1	1.8	1.8	1.9	1.9
Mmp12	1.7	1.9	2.0	1.9
Hspb2	2.0	2.1	1.5	1.8
Egr4	1.5	2.3	1.7	1.8
Aqp3	1.7	1.6	2.2	1.8
Zfp365	1.8	1.8	1.9	1.8
Lgals9	2.6	1.3	1.6	1.8
Arc	1.9	2.0	1.6	1.8
Fgf18	1.7	1.8	1.9	1.8
Dlk1	2.3	1.9	1.3	1.8
Vnn1	1.4	1.9	2.1	1.8
Ptchd4	1.5	2.2	1.7	1.8
Robo2	1.7	1.8	1.9	1.8
Sgk2	1.4	1.9	2.1	1.8
Tfap2b	2.0	2.0	1.3	1.8
Pgr	1.5	1.8	2.1	1.8
Gnas	1.8	2.1	1.5	1.8
Rgs5	1.7	1.8	1.9	1.8
Rgs4	1.6	1.8	1.9	1.8
Ctgf	1.7	1.9	1.8	1.8
Oas1a	1.8	1.6	2.0	1.8
Lrrc75b	1.7	2.1	1.6	1.8
Figf	1.9	2.1	1.3	1.8
Rab38	1.7	1.9	1.8	1.8
Nov	1.8	1.8	1.7	1.8
Perp	1.9	1.8	1.7	1.8
Gm10387	1.4	2.7	1.2	1.8
Grem1	1.7	1.8	1.8	1.8
Cyp1b1	1.4	1.7	2.2	1.8
Six2	1.6	1.7	2.0	1.8

Gene symbol	FC 1	FC 2	FC 3	Avg FC
Sytl2	2.0	1.5	1.8	1.8
TC1654523	1.6	2.1	1.6	1.8
Aldh1a2	1.8	1.8	1.6	1.7
Ndrg1	1.9	1.9	1.5	1.7
Kcnq5	1.9	1.9	1.5	1.7
Cited1	1.7	2.0	1.6	1.7
Zmat4	1.7	1.9	1.7	1.7
Tmem132c	1.6	1.8	1.8	1.7
Adra1d	1.8	1.8	1.6	1.7
Egr2	1.6	1.7	2.0	1.7
Mtus2	1.4	1.5	2.3	1.7
Ntm	1.6	1.8	1.8	1.7
Gm11783	1.5	2.1	1.6	1.7
Trp63	1.3	2.3	1.6	1.7
Gabrb2	1.8	2.0	1.4	1.7
Wnt6	1.6	1.6	2.0	1.7
Wbscr17	1.6	2.0	1.5	1.7
Rarres1	1.8	1.5	1.8	1.7
Itga7	1.9	1.8	1.5	1.7
Xaf1	1.7	1.3	2.2	1.7
Ascl2	1.9	1.3	1.9	1.7
Pip5k1a	1.6	1.8	1.7	1.7
Dclk2	2.0	1.7	1.5	1.7
Plekha4	1.7	1.8	1.7	1.7
Pitx2	1.6	1.8	1.7	1.7
Stc1	1.8	1.6	1.7	1.7
Trim47	1.6	1.7	1.7	1.7
Ctsc	1.7	2.0	1.4	1.7
A430106G13Rik	1.3	2.3	1.5	1.7
Dkk2	1.5	1.8	1.7	1.7
Cyr61	1.6	1.8	1.7	1.7
D630013G24Rik	1.5	1.8	1.6	1.7
Bgn	2.0	1.6	1.4	1.7
Ddah1	1.6	1.8	1.6	1.7
Osmr	2.0	1.8	1.2	1.7
Epha3	1.5	1.5	1.9	1.7
Nr4a1	1.8	1.7	1.5	1.7
Actg2	1.6	1.8	1.5	1.6
Ptgs2	1.6	1.8	1.5	1.6
D630010B17Rik	1.6	1.7	1.6	1.6
Nfkbiz	1.7	2.0	1.3	1.6
Prrx2	1.5	1.6	1.8	1.6
Nkd2	1.5	1.7	1.7	1.6
Nrtn	1.6	1.7	1.6	1.6
Agtr1a	1.6	1.7	1.5	1.6
Fgf12	1.8	1.7	1.4	1.6
Klhl14	1.3	1.7	1.9	1.6
Thrb	1.3	1.8	1.8	1.6
March11	1.5	1.7	1.6	1.6
Sfmbt2	1.3	1.6	1.9	1.6
Calcrl	2.0	1.4	1.4	1.6
Lgi2	1.7	1.5	1.6	1.6
LOC102641377	1.4	1.5	1.9	1.6
Btc	1.8	1.6	1.4	1.6
Chl1	1.5	1.7	1.6	1.6
A4galt	1.4	1.7	1.7	1.6
Col7a1	1.4	1.7	1.7	1.6
Slc23a3	1.4	1.5	2.0	1.6
Hopx	2.1	1.4	1.3	1.6
Ifi27l2a	1.8	1.3	1.6	1.6
Fam150b	1.7	1.8	1.3	1.6
Foxf2	1.5	1.5	1.8	1.6
Hist2h2bb	1.6	1.8	1.4	1.6
Tcp11	1.6	1.7	1.5	1.6

Gene symbol	FC 1	FC 2	FC 3	Avg FC
Ace2	1.7	1.7	1.4	1.6
Tnc	1.7	1.5	1.6	1.6
Gas2	1.4	1.5	1.8	1.6
Kcnn2	1.7	1.7	1.3	1.6
AI594674	1.4	2.1	1.3	1.6
Lhfpl3	1.9	1.4	1.5	1.6
Itga11	1.4	1.7	1.6	1.6
ENSMUST000000996	1.6	1.2	1.9	1.6
Ptchd1	1.6	1.6	1.5	1.6
Cd44	1.7	1.7	1.3	1.6
Ankrd1	1.6	1.6	1.5	1.6
Ppp1r3d	1.5	1.6	1.7	1.6
Jakmip1	1.5	1.8	1.5	1.6
Gfra2	1.7	1.6	1.4	1.6
Pdgfrb	1.6	1.6	1.4	1.6
Fas	1.4	1.7	1.5	1.6
Hand1	1.9	1.5	1.4	1.6
Atp6v0a4	1.5	1.6	1.6	1.6
F2rl1	1.5	1.6	1.5	1.6
Dusp2	1.5	1.5	1.6	1.6
Nrp2	1.6	1.9	1.2	1.6
Rspo1	1.2	1.7	1.8	1.6
Ntrk3	1.7	1.7	1.3	1.6
Gpc4	1.5	1.7	1.5	1.6
Rasgrp2	1.7	1.5	1.4	1.6
1500015O10Rik	1.5	1.7	1.5	1.6
Npas4	1.6	1.5	1.5	1.6
Col5a3	1.7	1.6	1.4	1.6
Lmo1	1.6	1.6	1.5	1.6
Lrrtm1	1.6	1.7	1.4	1.6
Cacna2d3	1.5	1.5	1.6	1.6
Tiam2	1.5	1.7	1.5	1.5
Syt13	1.5	1.8	1.3	1.5
Tmem26	1.6	1.6	1.5	1.5
Frzb	1.5	1.6	1.5	1.5
Tmem40	1.6	1.5	1.5	1.5
AY512938	1.4	1.4	1.7	1.5
Col12a1	1.5	1.5	1.6	1.5
Ahrr	1.2	1.5	2.0	1.5
Evx1	1.5	1.4	1.7	1.5
Tgfb1	1.6	1.6	1.3	1.5
Mamdc2	1.6	1.3	1.7	1.5
1810011O10Rik	1.5	1.4	1.7	1.5
Inhbb	1.6	1.8	1.3	1.5
Pcdh8	1.5	1.8	1.3	1.5
ENSMUST000000543	1.3	1.7	1.6	1.5
Arid5a	1.5	1.6	1.5	1.5
Ssfa2	1.7	1.5	1.3	1.5
Lum	1.5	1.4	1.6	1.5
Itga4	1.5	1.6	1.5	1.5
Ism1	1.5	1.6	1.4	1.5
Fos	1.4	1.5	1.6	1.5
Gadd45b	1.6	1.5	1.5	1.5
Plec	1.5	1.7	1.4	1.5
Rgs7bp	1.2	1.6	1.7	1.5
Parp12	1.6	1.3	1.7	1.5
Ifitm1	1.6	1.6	1.3	1.5
Map6	1.5	1.5	1.5	1.5
Magel2	1.6	1.3	1.6	1.5
Fam83g	1.4	1.7	1.4	1.5
S100a13	1.4	1.6	1.6	1.5
Aspn	1.6	1.5	1.5	1.5
5330426P16Rik	1.4	1.4	1.6	1.5
Calcr	1.5	1.5	1.4	1.5

Gene symbol	FC 1	FC 2	FC 3	Avg FC
NAP061760-1	1.8	1.4	1.3	1.5
Jag1	1.5	1.5	1.4	1.5
Inhba	1.5	1.8	1.3	1.5
Dgkk	1.4	1.7	1.4	1.5
Pamr1	1.4	1.7	1.5	1.5
Ccdc3	1.5	1.7	1.3	1.5
Rhob	1.4	1.5	1.5	1.5
Maob	1.3	1.5	1.7	1.5
Sfn	1.7	1.5	1.3	1.5
Cspg4	1.5	1.6	1.4	1.5
Cryab	1.9	1.3	1.3	1.5
2010111I01Rik	1.4	1.4	1.7	1.5
Ptprd	1.2	1.9	1.3	1.5
Runx1	1.7	1.3	1.4	1.5
Klc3	1.5	1.5	1.5	1.5
Acta1	1.7	1.4	1.4	1.5
Sytl1	1.4	1.4	1.6	1.5
Mmrn1	1.4	1.6	1.4	1.5
Cap2	1.7	1.3	1.5	1.5
Gm2347	1.2	1.9	1.3	1.5
Fxyd2	1.2	1.4	1.8	1.5
Ddx58	1.2	1.5	1.7	1.5
Phldb3	1.4	1.5	1.6	1.5
Kctd12	1.5	1.4	1.5	1.5
AK036131	1.3	1.7	1.4	1.5
Map3k6	1.5	1.5	1.5	1.5
Fbn2	1.6	1.7	1.2	1.5
Hspb1	1.5	1.2	1.6	1.5
Fn1	1.5	1.3	1.6	1.5
Rin1	1.4	1.6	1.4	1.5
Bdnf	1.3	1.5	1.6	1.5
Mme	1.6	1.5	1.4	1.5
Htra1	1.3	1.6	1.5	1.5
Chmp4c	1.6	1.6	1.2	1.5
Cdkn2b	1.5	1.6	1.3	1.5
1700040L02Rik	1.3	1.7	1.5	1.5
Dmrta1	1.3	1.6	1.5	1.5
C1qtnf2	1.8	1.2	1.4	1.5
Vwa2	1.5	1.5	1.4	1.5
Npr3	1.3	1.4	1.7	1.5
Tmem100	1.5	1.5	1.4	1.5
Celsr1	1.4	1.6	1.5	1.5
Sh3d19	1.5	1.4	1.5	1.5
Rgs8	1.3	1.7	1.4	1.5
Pmaip1	1.4	1.6	1.4	1.5
Plk3	1.4	1.5	1.5	1.5
Traf1	1.4	1.6	1.4	1.5
Krt13	1.5	1.5	1.3	1.5
Rnf150	1.3	1.2	1.9	1.5
1500009L16Rik	1.4	1.4	1.6	1.5
Rasgef1b	1.4	1.5	1.5	1.5
Gsn	1.4	1.5	1.4	1.5
Loxl1	1.7	1.4	1.2	1.5
Sync	1.4	1.4	1.5	1.5
Mef2c	1.5	1.6	1.3	1.4
Phlda2	1.5	1.3	1.5	1.4
Ier3	1.3	1.3	1.7	1.4
Prdm6	1.5	1.4	1.5	1.4
2610018G03Rik	1.7	1.2	1.5	1.4
Ptn	1.6	1.4	1.4	1.4
Adamts14	1.4	1.5	1.4	1.4
Lxn	1.2	1.6	1.5	1.4
Cited2	1.3	1.2	1.8	1.4
A_55_P1970825	1.8	1.3	1.3	1.4

Gene symbol	FC 1	FC 2	FC 3	Avg FC
Ogfrl1	1.5	1.4	1.5	1.4
Grem2	1.5	1.5	1.4	1.4
Lyst	1.8	1.3	1.2	1.4
2210011C24Rik	1.3	1.7	1.3	1.4
Wnt11	1.2	1.4	1.7	1.4
Ptk2b	1.5	1.5	1.3	1.4
Lilrb4	1.5	1.3	1.5	1.4
Id4	1.5	1.5	1.3	1.4
Vav3	1.4	1.5	1.4	1.4
Sqrdl	1.4	1.6	1.3	1.4
Klf8	1.3	1.5	1.6	1.4
Cth	1.5	1.5	1.3	1.4
Rem2	1.5	1.6	1.3	1.4
Actc1	1.5	1.4	1.5	1.4
Rdh10	1.4	1.3	1.6	1.4
Fam222a	1.3	1.5	1.5	1.4
Npb	1.2	1.5	1.6	1.4
Chst1	1.4	1.5	1.4	1.4
Cdo1	1.4	1.5	1.4	1.4
Dpp4	1.3	1.5	1.5	1.4
Dusp8	1.6	1.5	1.2	1.4
Smoc2	1.6	1.3	1.4	1.4
Egr1	1.3	1.5	1.5	1.4
Il1r1	1.5	1.6	1.3	1.4
Lox	1.4	1.5	1.4	1.4
Hspa1a	1.4	1.3	1.6	1.4
Il17rc	1.4	1.4	1.5	1.4
Kif21b	1.4	1.6	1.3	1.4
Grin3a	1.4	1.4	1.5	1.4
March3	1.6	1.4	1.3	1.4
Lrrn3	1.4	1.3	1.6	1.4
Mical2	1.4	1.6	1.3	1.4
Slc6a13	1.5	1.4	1.3	1.4
Runx1t1	1.4	1.4	1.5	1.4
Degs2	1.5	1.3	1.5	1.4
Scml4	1.4	1.4	1.4	1.4
Cnksr1	1.6	1.4	1.3	1.4
Pdlim2	1.7	1.3	1.3	1.4
Nog	1.7	1.3	1.2	1.4
Akr1c19	1.5	1.5	1.3	1.4
Palm3	1.3	1.4	1.5	1.4
ENSMUST00000074976	1.3	1.4	1.6	1.4
Lmna	1.6	1.4	1.2	1.4
Plcl1	1.4	1.6	1.2	1.4
Igfbp2	1.5	1.4	1.3	1.4
Ldlrad4	1.4	1.5	1.4	1.4
Cthrc1	1.3	1.4	1.5	1.4
Tep1	1.4	1.5	1.3	1.4
Ramp2	1.5	1.4	1.3	1.4
A_55_P2180347	1.4	1.2	1.6	1.4
Adamts5	1.8	1.2	1.2	1.4
2610316D01Rik	1.5	1.3	1.4	1.4
Cav1	1.6	1.4	1.2	1.4
Fbln2	1.4	1.6	1.2	1.4
Olfml2b	1.5	1.4	1.3	1.4
Cdh4	1.4	1.4	1.3	1.4
9330159M07Rik	1.2	1.3	1.7	1.4
Ldoc1	1.2	1.4	1.6	1.4
Nfatc1	1.4	1.4	1.4	1.4
Ckmt1	1.2	1.4	1.5	1.4
Nup62cl	1.4	1.4	1.4	1.4
Tacstd2	1.5	1.4	1.3	1.4
Nrbp2	1.4	1.4	1.3	1.4
B930095G15Rik	1.4	1.4	1.3	1.4

Gene symbol	FC 1	FC 2	FC 3	Avg FC
1700011H14Rik	1.2	1.6	1.4	1.4
Mmd	1.3	1.4	1.4	1.4
Ifi35	1.6	1.3	1.3	1.4
Nap1l2	1.2	1.3	1.7	1.4
Thbs1	1.4	1.4	1.3	1.4
A_55_P1972975	1.2	1.3	1.7	1.4
Cdc42ep3	1.2	1.4	1.5	1.4
Galnt18	1.6	1.2	1.3	1.4
Slc7a8	1.2	1.6	1.3	1.4
Dok4	1.3	1.6	1.3	1.4
Trps1	1.3	1.5	1.3	1.4
S100a10	1.4	1.2	1.5	1.4
Efcab4a	1.3	1.5	1.4	1.4
Atp1b2	1.4	1.3	1.4	1.4
Fbln5	1.4	1.5	1.3	1.4
Fgd3	1.4	1.5	1.3	1.4
Prr5l	1.3	1.4	1.4	1.4
Klf2	1.2	1.4	1.5	1.4
Rspo3	1.3	1.3	1.5	1.4
Lrig3	1.2	1.5	1.3	1.4
Dlgap3	1.2	1.7	1.2	1.4
Rbm24	1.4	1.4	1.4	1.4
Ms4a6d	1.4	1.2	1.5	1.4
1110032F04Rik	1.3	1.4	1.4	1.4
Ahnak	1.4	1.2	1.5	1.4
Rrad	1.5	1.4	1.2	1.4
Gyltl1b	1.4	1.4	1.3	1.4
Spry2	1.4	1.3	1.4	1.4
Dysf	1.3	1.5	1.3	1.4
Bmp5	1.3	1.6	1.3	1.4
Bai3	1.4	1.4	1.3	1.4
Hist1h1c	1.5	1.2	1.3	1.4
Cav2	1.3	1.3	1.4	1.4
Zc3hav1	1.3	1.3	1.5	1.4
Diras2	1.5	1.3	1.3	1.4
Pdzk1ip1	1.3	1.2	1.5	1.4
Pnma2	1.3	1.5	1.2	1.4
A930017K11Rik	1.5	1.4	1.2	1.4
Mturn	1.3	1.3	1.5	1.4
Rhbdf1	1.3	1.5	1.3	1.4
Cadm1	1.4	1.3	1.3	1.4
Sparcl1	1.4	1.4	1.3	1.4
Fstl3	1.5	1.3	1.3	1.4
Tril	1.4	1.3	1.4	1.4
Fxyd5	1.5	1.3	1.2	1.4
Smim24	1.4	1.4	1.3	1.4
Psd	1.4	1.4	1.3	1.4
Ube2ql1	1.5	1.3	1.3	1.4
Prss12	1.2	1.3	1.5	1.4
Anxa1	1.4	1.2	1.4	1.4
Nfil3	1.3	1.3	1.5	1.4
Arid5b	1.3	1.4	1.3	1.4
Fam217b	1.2	1.5	1.3	1.4
Rnd3	1.2	1.3	1.5	1.3
Emp1	1.3	1.3	1.4	1.3
Inf2	1.3	1.4	1.3	1.3
Stat5a	1.5	1.3	1.2	1.3
Nxph3	1.3	1.4	1.3	1.3
Htra4	1.4	1.3	1.3	1.3
Filip1l	1.5	1.3	1.3	1.3
Mir22hg	1.4	1.3	1.4	1.3
D430041D05Rik	1.4	1.4	1.2	1.3
Heyl	1.3	1.5	1.2	1.3
Oas1f	1.4	1.3	1.4	1.3
Sh3tc2	1.4	1.4	1.2	1.3
Sp6	1.3	1.3	1.4	1.3
Fam129b	1.3	1.4	1.3	1.3
Nuak2	1.3	1.5	1.2	1.3
Zfp608	1.3	1.3	1.4	1.3
Slc16a14	1.3	1.3	1.4	1.3
Tfcp2l1	1.3	1.3	1.4	1.3
9130022E09	1.3	1.4	1.3	1.3
Dgkh	1.3	1.3	1.4	1.3
Lgals3	1.3	1.4	1.2	1.3
Fibin	1.3	1.5	1.2	1.3
6430519N07Rik	1.2	1.4	1.4	1.3
Mapk4	1.3	1.4	1.3	1.3
Mafk	1.4	1.3	1.3	1.3
Tle4	1.3	1.3	1.3	1.3
Tbx2	1.2	1.2	1.5	1.3
LOC102634598	1.3	1.4	1.3	1.3
Emb	1.4	1.3	1.3	1.3
Rgs16	1.4	1.4	1.2	1.3
Tagln	1.3	1.3	1.4	1.3
Fgfr1	1.6	1.2	1.2	1.3
Myadm	1.4	1.4	1.2	1.3
Man2a1	1.2	1.5	1.3	1.3
Pcdh19	1.3	1.3	1.4	1.3
C77080	1.3	1.3	1.4	1.3
1110006E14Rik	1.3	1.5	1.2	1.3
Fam13c	1.3	1.4	1.3	1.3
A130072N09Rik	1.2	1.5	1.3	1.3
Slamf9	1.3	1.5	1.2	1.3
Mt1	1.3	1.3	1.4	1.3
Lamc2	1.2	1.4	1.3	1.3
Prkg2	1.2	1.5	1.3	1.3
Cys1	1.3	1.3	1.4	1.3
Rhcg	1.2	1.3	1.4	1.3
Rap1gap	1.2	1.3	1.4	1.3
Dmtn	1.3	1.4	1.3	1.3
Enho	1.4	1.2	1.3	1.3
Gxylt2	1.3	1.4	1.3	1.3
Cd109	1.3	1.4	1.3	1.3
Pdgfa	1.2	1.5	1.2	1.3
Gch1	1.4	1.2	1.3	1.3
Gng11	1.3	1.4	1.2	1.3
Phactr1	1.3	1.3	1.3	1.3
Gadd45g	1.4	1.2	1.3	1.3
Numbl	1.2	1.2	1.4	1.3
Fam83h	1.4	1.3	1.2	1.3
Smim1	1.5	1.2	1.2	1.3
Frmpd4	1.2	1.3	1.4	1.3
Prkcdbp	1.3	1.4	1.2	1.3
Hbegf	1.3	1.2	1.4	1.3
Fam212a	1.3	1.2	1.4	1.3
Plet1	1.3	1.3	1.3	1.3
Adk	1.3	1.4	1.2	1.3
Itga8	1.3	1.3	1.3	1.3
Cobl	1.3	1.4	1.2	1.3
Amotl2	1.3	1.3	1.3	1.3
Hoxd12	1.2	1.3	1.4	1.3
2310061J03Rik	1.3	1.3	1.3	1.3
A_55_P2005672	1.3	1.3	1.4	1.3
Arl4a	1.3	1.3	1.4	1.3
Itprip	1.3	1.3	1.3	1.3
Zfp503	1.3	1.4	1.2	1.3
Myo1c	1.2	1.4	1.3	1.3
Maff	1.2	1.3	1.3	1.3

Gene symbol	FC 1	FC 2	FC 3	Avg FC
Gpr176	1.3	1.2	1.4	1.3
Gjb3	1.4	1.3	1.2	1.3
Lhfpl1	1.4	1.2	1.3	1.3
Atp1a2	1.4	1.2	1.2	1.3
Sgpp2	1.3	1.4	1.2	1.3
Cybrd1	1.2	1.3	1.4	1.3
Myh14	1.2	1.4	1.3	1.3
Tjp3	1.3	1.3	1.2	1.3
Timp3	1.2	1.4	1.3	1.3
Eif4e3	1.3	1.2	1.3	1.3
Actn1	1.2	1.3	1.3	1.3
Ndufa4l2	1.3	1.4	1.2	1.3
Sertad1	1.4	1.2	1.3	1.3
Kremen1	1.3	1.3	1.3	1.3
Lifr	1.3	1.2	1.4	1.3
Timp1	1.3	1.4	1.2	1.3
Ankrd29	1.3	1.3	1.2	1.3
Ctbp2	1.3	1.2	1.3	1.3
Csf1	1.3	1.3	1.2	1.3
8430408G22Rik	1.3	1.2	1.3	1.3
Hnf1a	1.4	1.2	1.2	1.3
Hmga2-ps1	1.2	1.3	1.3	1.3
Enc1	1.2	1.4	1.2	1.3
D9Ertd115e	1.2	1.2	1.4	1.3
Plcxd3	1.2	1.3	1.3	1.3
Bcl10	1.3	1.3	1.3	1.3
Plau	1.3	1.3	1.2	1.3
Capg	1.3	1.3	1.2	1.3
Srpx	1.2	1.3	1.3	1.3
Lgals1	1.2	1.3	1.3	1.3
Phf1	1.2	1.3	1.3	1.3
Nfkbie	1.3	1.2	1.3	1.3
Clec4d	1.3	1.2	1.3	1.3
Dgat2	1.2	1.3	1.3	1.3
Tbc1d9	1.2	1.3	1.3	1.3
Msn	1.2	1.3	1.2	1.3
Cebpd	1.2	1.3	1.2	1.3
Caps2	1.2	1.3	1.3	1.3
Gm3364	1.2	1.3	1.3	1.3
Scube3	1.2	1.3	1.2	1.3
Osbpl8	1.2	1.2	1.3	1.3
Hoxb9	1.3	1.2	1.2	1.3
LOC102642487	1.3	1.2	1.3	1.3
Dleu2	1.2	1.3	1.2	1.3
Tob1	1.2	1.2	1.3	1.2
Flot2	1.2	1.3	1.2	1.2
Tnfaip3	1.2	1.3	1.2	1.2
Slc12a2	1.2	1.3	1.2	1.2
Cacna1c	1.2	1.3	1.3	1.2
Ptx3	1.3	1.2	1.2	1.2
Foxp1	1.2	1.3	1.2	1.2
Ank	1.2	1.2	1.3	1.2
Acvrl1	1.3	1.2	1.2	1.2
Hoxd3os1	1.2	1.3	1.2	1.2
Lrp2	1.2	1.2	1.3	1.2
Pdp1	1.2	1.2	1.3	1.2
Wnt7b	1.3	1.2	1.2	1.2
Tubg2	1.2	1.2	1.2	1.2
Hdac5	1.2	1.2	1.2	1.2
Adamtsl1	1.2	1.3	1.2	1.2
Sh2d3c	1.2	1.2	1.2	1.2
Dnajb4	1.2	1.2	1.2	1.2

Supplementary Table S7. Transcripts identified by microarray analysis that were downregulated upon RA treatment in the early ureter. Shown is a list of transcripts that were downregulated after RA treatment of E12.5 ureter explants. Three groups for each condition were compared to untreated controls and the resulting fold changes (FC) in expression are displayed. Intensity thresholds were >100; fold changes were larger than 1.2.

Gene symbol	FC 1	FC 2	FC 3	Avg FC
Insm2	-4.7	-9.4	-7.5	-7.2
Gabra1	-5.3	-5.8	-5.9	-5.7
Nts	-3.4	-4.0	-9.5	-5.6
Ace2	-6.1	-5.1	-4.9	-5.4
Npy	-5.4	-4.9	-5.4	-5.2
A930009L07Rik	-3.1	-7.2	-4.8	-5.0
Chgb	-3.6	-6.1	-4.6	-4.8
Rgs5	-3.3	-5.5	-5.5	-4.8
Ugt2b34	-4.3	-4.8	-5.0	-4.7
Fam150b	-2.8	-3.1	-7.8	-4.6
Rgs4	-4.1	-3.5	-5.2	-4.3
Akr1b7	-5.3	-3.0	-4.6	-4.3
Serpine1	-2.8	-2.5	-7.3	-4.2
Rgcc	-2.6	-3.9	-6.1	-4.2
Grm7	-4.4	-3.7	-4.2	-4.1
Dkk1	-4.3	-3.5	-4.4	-4.1
S100a4	-3.7	-2.8	-5.0	-3.8
Cartpt	-2.4	-4.1	-4.9	-3.8
Ctgf	-3.0	-3.1	-5.1	-3.7
Dkk2	-3.0	-3.4	-4.5	-3.6
Krt6a	-2.9	-2.8	-4.9	-3.6
Egr2	-2.6	-2.4	-5.3	-3.4
Gnas	-2.8	-3.6	-3.5	-3.3
Gm11783	-2.3	-3.9	-3.6	-3.3
Lum	-2.7	-3.5	-3.2	-3.1
Ltbp2	-2.4	-2.2	-4.6	-3.1
Penk	-2.6	-3.2	-3.2	-3.0
Dbh	-2.1	-3.5	-3.5	-3.0
Vcam1	-2.8	-3.0	-3.1	-3.0
Gja5	-2.7	-2.2	-3.8	-2.9
Chga	-2.0	-3.3	-3.4	-2.9
Tox	-3.1	-2.4	-3.1	-2.9
Serpina3i	-1.7	-1.7	-5.2	-2.8
Serpina3f	-1.9	-2.0	-4.5	-2.8
Gabrb2	-3.0	-2.3	-3.1	-2.8
Agtr2	-2.5	-3.0	-2.7	-2.8
Anxa8	-2.4	-2.2	-3.7	-2.8
Tm4sf1	-1.7	-1.8	-4.7	-2.7
Fas	-1.9	-2.2	-4.2	-2.7
Hr	-3.0	-2.6	-2.6	-2.7
Rasd2	-3.1	-2.5	-2.5	-2.7
Lmo1	-2.2	-2.8	-3.0	-2.6
Tfap2b	-2.4	-2.4	-2.9	-2.6
Krt5	-2.1	-1.9	-3.7	-2.5
Epha3	-2.4	-2.8	-2.4	-2.5
Kcnip4	-1.8	-2.7	-3.1	-2.5
Lrrc75b	-2.5	-2.4	-2.6	-2.5
Pfkp	-2.0	-2.0	-3.3	-2.5
Aqp3	-2.8	-2.7	-1.9	-2.5
Plau	-2.1	-2.4	-2.9	-2.5
Wif1	-2.4	-2.4	-2.3	-2.4
D930019F10Rik	-2.0	-3.4	-1.8	-2.4
Zfp365	-2.6	-2.2	-2.2	-2.3
Pcdh8	-2.2	-2.6	-2.2	-2.3
Rab38	-1.7	-2.1	-3.1	-2.3
Dcn	-1.9	-2.6	-2.3	-2.3
Figf	-2.1	-2.1	-2.8	-2.3
Kcnab1	-2.3	-1.8	-2.8	-2.3
Ptn	-2.3	-2.3	-2.3	-2.3
Aard	-2.2	-2.1	-2.6	-2.3
Lrrtm1	-2.1	-2.2	-2.6	-2.3
Gm6403	-2.4	-2.1	-2.3	-2.3
Fmod	-2.5	-1.9	-2.4	-2.3
Gpr17	-1.7	-2.5	-2.6	-2.3
Actg2	-2.1	-1.7	-2.9	-2.2
Tnfrsf12a	-1.5	-1.7	-3.5	-2.2
Actc1	-2.0	-2.2	-2.5	-2.2
Fam19a1	-1.9	-2.5	-2.3	-2.2
Trim29	-2.2	-1.4	-3.1	-2.2
Sema3g	-2.3	-1.8	-2.6	-2.2
Kcnc2	-3.0	-2.0	-1.7	-2.2
Ly6c1	-1.8	-1.6	-3.3	-2.2
Nrip3	-2.3	-2.6	-1.7	-2.2
Ly6a	-1.6	-1.6	-3.3	-2.2
Scin	-2.1	-2.1	-2.4	-2.2
Ptchd1	-2.1	-2.3	-2.2	-2.2
Perp	-2.0	-1.5	-3.0	-2.2
Cav2	-2.0	-1.9	-2.7	-2.2
Maob	-2.1	-2.2	-2.2	-2.2
Fzd9	-2.0	-2.0	-2.4	-2.1
S100a6	-1.7	-1.7	-3.0	-2.1
Arsj	-1.9	-2.6	-1.9	-2.1
Plcxd3	-1.7	-2.6	-2.1	-2.1
Rasgef1a	-2.4	-1.9	-2.1	-2.1
Gfra2	-2.2	-2.3	-1.9	-2.1
Lrrn3	-2.1	-2.2	-1.9	-2.1
Rbfox1	-1.8	-2.0	-2.5	-2.1
Trim47	-1.8	-1.6	-2.9	-2.1
Slc26a7	-2.6	-1.7	-2.0	-2.1
Fbln5	-2.1	-2.2	-2.0	-2.1
Plekha4	-1.9	-1.9	-2.4	-2.1
Grem2	-2.3	-2.0	-1.9	-2.1
Cav1	-1.7	-1.6	-2.9	-2.1
Lox	-2.5	-1.9	-1.9	-2.1
Sdpr	-2.1	-2.1	-2.0	-2.1
Mamdc2	-2.2	-1.8	-2.2	-2.1
Tagln	-1.7	-1.6	-2.7	-2.0
Postn	-1.7	-2.3	-2.1	-2.0
Dgkk	-1.8	-2.7	-1.6	-2.0
Scube2	-2.1	-2.3	-1.6	-2.0
Plet1	-1.8	-2.0	-2.2	-2.0
Cyr61	-1.9	-1.7	-2.5	-2.0
Pdgfrb	-2.3	-1.8	-1.9	-2.0
Hand1	-1.5	-2.0	-2.5	-2.0
Filip1l	-1.8	-1.9	-2.3	-2.0
Col14a1	-2.1	-1.6	-2.2	-2.0
Ntrk2	-1.9	-1.7	-2.4	-2.0
Gm7325	-1.8	-1.8	-2.4	-2.0

Gene symbol	FC 1	FC 2	FC 3	Avg FC
Mgp	-1.5	-1.8	-2.6	-2.0
Sfrp2	-2.1	-1.8	-2.0	-2.0
Aldh1a2	-2.3	-1.8	-1.8	-2.0
Anxa1	-1.5	-1.8	-2.6	-2.0
Th	-1.5	-1.9	-2.5	-2.0
Syt1	-2.2	-2.0	-1.7	-1.9
Dlk1	-1.7	-1.4	-2.7	-1.9
Cx3cl1	-1.5	-1.3	-3.1	-1.9
Glis1	-2.1	-2.0	-1.7	-1.9
Timp3	-1.7	-2.0	-2.1	-1.9
Btbd17	-1.7	-1.8	-2.3	-1.9
Fos	-1.6	-1.8	-2.4	-1.9
Ryr3	-1.6	-1.9	-2.3	-1.9
Egr1	-2.0	-1.7	-2.0	-1.9
Tek	-2.0	-1.6	-2.2	-1.9
Robo2	-2.2	-2.1	-1.5	-1.9
Frzb	-2.0	-1.7	-2.1	-1.9
Cd44	-1.5	-1.9	-2.4	-1.9
A_55_P1953377	-1.6	-1.4	-2.8	-1.9
Osr2	-2.1	-1.4	-2.2	-1.9
Btc	-2.0	-1.8	-1.9	-1.9
Sytl4	-1.7	-2.0	-2.0	-1.9
Stc1	-1.7	-2.2	-1.8	-1.9
Nr4a1	-1.7	-1.5	-2.5	-1.9
Fbln2	-1.8	-2.0	-1.9	-1.9
Matn4	-2.0	-1.9	-1.8	-1.9
Six2	-2.3	-1.4	-1.9	-1.9
Pmaip1	-1.4	-1.4	-2.9	-1.9
Thbs2	-1.7	-1.8	-2.1	-1.9
Itga11	-2.2	-1.3	-2.1	-1.9
1810011O10Rik	-1.5	-1.7	-2.4	-1.9
Rspo3	-1.8	-1.9	-1.9	-1.9
Ier3	-1.6	-1.5	-2.5	-1.9
Pappa2	-2.2	-1.5	-1.9	-1.9
4930431P19Rik	-2.4	-1.5	-1.6	-1.9
Hopx	-1.7	-1.7	-2.2	-1.9
Gp49a	-2.0	-1.4	-2.1	-1.9
Hpgd	-1.8	-1.5	-2.3	-1.8
Nup62cl	-1.8	-1.3	-2.4	-1.8
Gas2	-1.6	-1.8	-2.1	-1.8
Ctsc	-1.6	-1.5	-2.3	-1.8
Abcc9	-1.9	-1.6	-2.0	-1.8
Kctd12	-2.0	-2.1	-1.3	-1.8
Smoc1	-2.0	-1.4	-2.0	-1.8
Rarres1	-1.6	-1.6	-2.2	-1.8
Nfkbiz	-1.9	-1.5	-2.0	-1.8
Daf2	-1.8	-1.9	-1.7	-1.8
2700022O18Rik	-1.9	-1.8	-1.7	-1.8
Slc23a3	-1.9	-1.9	-1.6	-1.8
Bgn	-1.5	-1.5	-2.4	-1.8
Pcdh10	-1.7	-1.6	-2.1	-1.8
Tnfaip3	-1.7	-1.5	-2.1	-1.8
Kcnn2	-1.9	-1.6	-1.8	-1.8
Gem	-1.8	-1.5	-2.0	-1.8
Casp4	-1.3	-1.3	-2.7	-1.8
Ntrk3	-1.7	-1.4	-2.2	-1.8
Lhfpl3	-1.3	-2.2	-1.9	-1.8
Acta1	-1.2	-1.3	-2.7	-1.8
Sytl2	-1.9	-1.6	-1.9	-1.8
Myh11	-1.8	-1.3	-2.2	-1.8
March11	-1.4	-1.7	-2.2	-1.8
Fam129a	-1.6	-1.9	-1.7	-1.8
Lypd3	-1.6	-1.4	-2.3	-1.8
Rasgef1b	-1.9	-1.7	-1.7	-1.8

Gene symbol	FC 1	FC 2	FC 3	Avg FC
BC057651	-1.8	-1.6	-1.9	-1.8
Vnn1	-1.8	-1.8	-1.7	-1.8
Grhl3	-2.3	-1.2	-1.8	-1.8
Pitx2	-2.0	-1.5	-1.8	-1.8
Hmcn1	-1.9	-1.6	-1.7	-1.8
Zmat4	-1.6	-1.3	-2.4	-1.8
Mcam	-1.4	-1.3	-2.5	-1.8
Kcnq5	-2.1	-1.4	-1.8	-1.8
Ptchd4	-1.7	-1.5	-2.0	-1.8
Clec4d	-1.6	-1.5	-2.1	-1.7
5430435G22Rik	-2.0	-1.6	-1.6	-1.7
Pnma2	-1.9	-1.6	-1.7	-1.7
Glipr1	-1.3	-1.3	-2.7	-1.7
Fam46a	-1.5	-1.8	-1.9	-1.7
Tmem26	-1.6	-1.9	-1.7	-1.7
Mt2	-1.8	-1.9	-1.6	-1.7
Cryab	-1.4	-1.7	-2.1	-1.7
Kit	-1.9	-1.5	-1.7	-1.7
Gadd45b	-1.2	-1.6	-2.3	-1.7
Fxyd3	-1.6	-1.7	-1.9	-1.7
Rnd3	-2.1	-1.5	-1.5	-1.7
Cd55	-1.7	-2.1	-1.4	-1.7
Otor	-1.8	-1.4	-2.0	-1.7
LOC102636514	-1.4	-1.8	-1.9	-1.7
Tnfaip6	-1.3	-1.7	-2.1	-1.7
Smoc2	-1.7	-1.6	-1.8	-1.7
6430411K18Rik	-1.5	-1.7	-2.0	-1.7
Tmem132c	-2.0	-1.6	-1.5	-1.7
Artn	-1.8	-1.5	-1.8	-1.7
Mme	-2.1	-1.5	-1.5	-1.7
Svep1	-2.0	-1.4	-1.7	-1.7
1810041L15Rik	-1.6	-1.8	-1.8	-1.7
Nav3	-2.0	-1.4	-1.7	-1.7
Mfap5	-1.4	-1.7	-1.9	-1.7
Vav3	-1.8	-1.4	-1.9	-1.7
Spp1	-1.6	-1.6	-1.9	-1.7
Rdh10	-1.8	-1.5	-1.7	-1.7
Lilrb4	-1.8	-1.6	-1.7	-1.7
Lhfpl1	-1.6	-1.5	-2.0	-1.7
Paqr5	-1.5	-1.8	-1.7	-1.7
Lgals3	-1.8	-1.5	-1.8	-1.7
Kcnma1	-1.8	-1.5	-1.7	-1.7
Gpr97	-1.6	-1.5	-1.9	-1.7
Ctla2b	-1.7	-1.2	-2.1	-1.7
Dusp26	-1.3	-1.8	-1.9	-1.7
Adm	-1.4	-1.5	-2.1	-1.7
Thbs1	-1.5	-1.4	-2.0	-1.7
Ism1	-1.9	-1.5	-1.6	-1.7
Aplnr	-2.0	-1.2	-1.7	-1.7
Plac1	-1.7	-1.4	-1.8	-1.7
Ccdc3	-1.7	-1.5	-1.7	-1.7
Ctla2a	-1.8	-1.2	-1.9	-1.7
Pde1a	-1.4	-1.6	-1.9	-1.6
LOC102633497	-2.2	-1.5	-1.2	-1.6
Adam23	-1.9	-1.3	-1.7	-1.6
Wisp1	-1.4	-1.3	-2.2	-1.6
Il1r1	-1.6	-1.3	-2.1	-1.6
1110006E14Rik	-1.9	-1.5	-1.5	-1.6
Procr	-1.7	-1.6	-1.7	-1.6
Acta2	-1.6	-1.3	-2.0	-1.6
LOC102634502	-1.4	-1.8	-1.7	-1.6
Ssfa2	-1.7	-1.5	-1.7	-1.6
Slamf9	-1.4	-1.4	-2.2	-1.6
Sqrdl	-1.4	-1.5	-2.0	-1.6

Gene symbol	FC 1	FC 2	FC 3	Avg FC
Mogat2	-1.8	-1.3	-1.8	-1.6
Serpinb6b	-1.6	-1.3	-2.0	-1.6
Timp1	-1.3	-1.2	-2.4	-1.6
Rasgrp2	-1.5	-1.3	-2.0	-1.6
Fam13c	-1.8	-1.6	-1.5	-1.6
ENSMUST00000057427	-1.4	-1.4	-2.1	-1.6
2810055G20Rik	-1.7	-1.9	-1.2	-1.6
Fgf12	-1.6	-1.8	-1.4	-1.6
Phactr1	-1.6	-1.6	-1.6	-1.6
Ddah1	-1.3	-1.4	-2.1	-1.6
Msr1	-2.0	-1.4	-1.4	-1.6
Fxyd5	-1.3	-1.3	-2.2	-1.6
NAP061760-1	-1.2	-1.2	-2.4	-1.6
Acot11	-1.6	-1.5	-1.7	-1.6
Fgd3	-1.4	-1.3	-2.2	-1.6
ENSMUST00000103740	-1.2	-1.5	-2.0	-1.6
Alox5ap	-1.4	-1.4	-2.0	-1.6
2610018G03Rik	-1.4	-1.9	-1.6	-1.6
Agtr1a	-1.6	-1.5	-1.6	-1.6
Aldh1a1	-1.6	-1.5	-1.7	-1.6
Sh3gl2	-1.8	-1.7	-1.4	-1.6
Tmem40	-1.4	-1.4	-2.0	-1.6
Rxfp1	-1.6	-1.3	-1.9	-1.6
Ramp2	-1.5	-1.5	-1.8	-1.6
Sprr2a2	-1.5	-1.6	-1.8	-1.6
Fgfr4	-1.8	-1.5	-1.4	-1.6
Fat3	-1.5	-1.3	-2.0	-1.6
Serpine2	-1.4	-1.5	-1.9	-1.6
Krt15	-1.5	-1.3	-2.0	-1.6
Nrk	-1.6	-2.0	-1.2	-1.6
Vstm2l	-1.4	-1.5	-1.8	-1.6
Lgi2	-1.4	-1.9	-1.5	-1.6
Maff	-1.3	-1.3	-2.1	-1.6
Pdlim1	-1.3	-1.3	-2.1	-1.6
Zeb2os	-1.4	-2.0	-1.4	-1.6
Id4	-1.6	-1.5	-1.7	-1.6
Gpc4	-1.5	-1.2	-2.0	-1.6
Lmna	-1.4	-1.4	-1.9	-1.6
Adamts6	-1.3	-1.8	-1.6	-1.6
Slc18a2	-1.4	-1.6	-1.8	-1.6
Habp2	-1.6	-1.5	-1.6	-1.6
Pdzk1ip1	-1.5	-1.6	-1.7	-1.6
Syt4	-1.7	-1.7	-1.4	-1.6
Sfmbt2	-1.6	-1.7	-1.4	-1.6
Cdh6	-1.8	-1.6	-1.3	-1.6
Tnc	-1.4	-1.7	-1.6	-1.6
Ptx3	-1.4	-1.7	-1.6	-1.6
Loxl1	-1.5	-1.3	-1.9	-1.6
Spon1	-1.5	-1.6	-1.6	-1.6
Sox6	-1.7	-1.5	-1.5	-1.6
Nupr1	-1.2	-1.3	-2.2	-1.6
Mirg	-1.4	-1.8	-1.5	-1.6
Sox10	-1.6	-1.6	-1.5	-1.6
Nrxn1	-1.8	-1.5	-1.4	-1.6
Oxsm	-1.5	-1.5	-1.6	-1.6
C1qtnf2	-1.4	-1.6	-1.7	-1.6
Runx1t1	-1.6	-1.8	-1.3	-1.6
Bai3	-1.7	-1.4	-1.5	-1.6
Plac9a	-1.2	-1.4	-2.1	-1.6
Pdlim2	-1.4	-1.5	-1.7	-1.6
Adamtsl1	-1.9	-1.5	-1.3	-1.6
Kcnt2	-1.8	-1.4	-1.5	-1.6
Spry2	-1.3	-1.3	-2.0	-1.6
Ell2	-1.6	-1.4	-1.7	-1.6

Gene symbol	FC 1	FC 2	FC 3	Avg FC
Pcdh19	-1.7	-1.6	-1.4	-1.6
Fxyd2	-1.4	-1.7	-1.6	-1.6
Bmp5	-1.5	-1.6	-1.5	-1.6
TC1682680	-1.3	-1.3	-2.0	-1.6
Megf10	-1.7	-1.3	-1.7	-1.6
Tspo	-1.3	-1.2	-2.1	-1.6
Degs2	-1.4	-1.5	-1.7	-1.5
Cbr3	-1.3	-1.3	-2.0	-1.5
Adcy4	-1.5	-1.2	-1.9	-1.5
Rgs2	-1.3	-1.8	-1.5	-1.5
Srpx	-1.3	-1.6	-1.7	-1.5
Adamts14	-1.4	-1.4	-1.8	-1.5
Lamc3	-1.6	-1.4	-1.6	-1.5
Tacstd2	-1.6	-1.3	-1.7	-1.5
Sema5b	-1.5	-1.2	-1.9	-1.5
TC1657454	-1.2	-1.3	-2.1	-1.5
Fcgr3	-1.8	-1.3	-1.5	-1.5
AK013505	-1.3	-1.7	-1.7	-1.5
Gimap8	-1.6	-1.5	-1.5	-1.5
Itga4	-1.6	-1.5	-1.5	-1.5
Arid5a	-1.5	-1.4	-1.7	-1.5
TC1636769	-1.4	-1.6	-1.6	-1.5
Npas4	-1.5	-1.4	-1.7	-1.5
Cnn1	-1.2	-1.3	-2.1	-1.5
Lamb3	-1.4	-1.2	-1.9	-1.5
Itm2a	-1.7	-1.4	-1.4	-1.5
Osr1	-1.3	-1.5	-1.7	-1.5
Mef2c	-1.2	-1.6	-1.7	-1.5
NAP096516-001	-1.3	-1.4	-1.9	-1.5
Acot9	-1.4	-1.2	-1.9	-1.5
Lrrc4c	-1.4	-2.0	-1.2	-1.5
Trps1	-1.5	-1.7	-1.3	-1.5
Gm13315	-1.3	-1.5	-1.8	-1.5
Ptrf	-1.7	-1.3	-1.6	-1.5
Chst1	-1.6	-1.5	-1.5	-1.5
Angpt1	-1.6	-1.5	-1.4	-1.5
Acot10	-1.3	-1.4	-1.8	-1.5
Aldh1a7	-1.5	-1.5	-1.5	-1.5
A_55_P2118810	-1.3	-1.4	-1.8	-1.5
Cers3	-1.6	-1.4	-1.6	-1.5
March3	-1.3	-1.3	-2.0	-1.5
8430408G22Rik	-1.5	-1.3	-1.7	-1.5
A_55_P2063471	-1.3	-1.4	-1.8	-1.5
Cfhr2	-1.8	-1.3	-1.4	-1.5
Col2a1	-1.6	-1.4	-1.5	-1.5
Jag1	-1.5	-1.4	-1.6	-1.5
5330426P16Rik	-1.5	-1.5	-1.5	-1.5
Ctnna2	-1.4	-1.4	-1.7	-1.5
Dlgap1	-1.3	-1.5	-1.8	-1.5
Lix1	-1.5	-1.5	-1.5	-1.5
Sh3tc2	-1.5	-1.4	-1.6	-1.5
Fam49a	-1.3	-1.5	-1.7	-1.5
Stxbp6	-1.5	-1.6	-1.4	-1.5
A_55_P2168781	-1.3	-1.3	-1.9	-1.5
Cntn6	-1.7	-1.3	-1.5	-1.5
Dmrta2	-1.6	-1.5	-1.3	-1.5
9330159M07Rik	-1.3	-1.6	-1.5	-1.5
Vegfc	-1.5	-1.4	-1.6	-1.5
Arl4a	-1.3	-1.4	-1.8	-1.5
Ndrg1	-1.6	-1.2	-1.7	-1.5
A_55_P2037689	-1.3	-1.3	-1.8	-1.5
Ptprz1	-1.3	-1.4	-1.8	-1.5
Mmd	-1.4	-1.5	-1.6	-1.5
1500009L16Rik	-1.3	-1.5	-1.7	-1.5

Gene symbol	FC 1	FC 2	FC 3	Avg FC
Osbpl3	-1.3	-1.3	-1.9	-1.5
Cd34	-1.4	-1.4	-1.7	-1.5
C430049B03Rik	-1.4	-1.6	-1.4	-1.5
Kcne4	-1.2	-1.6	-1.6	-1.5
Bcl2l14	-1.4	-1.5	-1.6	-1.5
Fbn2	-1.5	-1.6	-1.4	-1.5
Slc5a7	-1.3	-1.3	-1.9	-1.5
Rprm	-1.3	-1.2	-1.9	-1.5
Ggact	-1.3	-1.6	-1.5	-1.5
Arid5b	-1.8	-1.3	-1.4	-1.5
Ddx5	-1.4	-1.4	-1.5	-1.5
ENSMUST00000050753	-1.4	-1.3	-1.8	-1.5
Pip5k1a	-1.5	-1.4	-1.5	-1.5
Fxyd4	-1.3	-1.5	-1.6	-1.5
Neat1	-1.5	-1.3	-1.6	-1.5
Atxn1	-1.5	-1.6	-1.4	-1.5
1700011H14Rik	-1.4	-1.4	-1.6	-1.5
Vcl	-1.5	-1.4	-1.6	-1.5
Ldha	-1.3	-1.4	-1.7	-1.5
Kcns3	-1.4	-1.5	-1.6	-1.5
Adora2b	-1.2	-1.5	-1.7	-1.5
D230018H15Rik	-1.7	-1.3	-1.4	-1.5
Cyp2s1	-1.7	-1.4	-1.3	-1.5
Pcolce	-1.5	-1.4	-1.5	-1.5
Prr5l	-1.4	-1.4	-1.5	-1.5
Emb	-1.5	-1.5	-1.3	-1.5
Pawr	-1.4	-1.4	-1.6	-1.5
Grin3a	-1.3	-1.5	-1.6	-1.5
Itga8	-1.8	-1.4	-1.2	-1.5
Il6st	-1.5	-1.3	-1.6	-1.5
Bean1	-1.5	-1.3	-1.6	-1.5
Sned1	-1.5	-1.4	-1.4	-1.5
Gsg1l	-1.6	-1.2	-1.5	-1.5
Cfh	-1.6	-1.5	-1.3	-1.5
Stbd1	-1.3	-1.5	-1.6	-1.5
Lxn	-1.4	-1.4	-1.5	-1.5
Hist2h2bb	-1.7	-1.4	-1.3	-1.5
S100a13	-1.4	-1.4	-1.6	-1.5
C3ar1	-1.6	-1.3	-1.4	-1.5
A_55_P1997061	-1.2	-1.3	-1.8	-1.5
Chmp2b	-1.4	-1.6	-1.4	-1.5
9430060I03Rik	-1.7	-1.3	-1.3	-1.5
Traf1	-1.4	-1.3	-1.6	-1.5
Tcerg1l	-1.5	-1.5	-1.4	-1.5
Ccl9	-1.5	-1.3	-1.6	-1.4
Gm4132	-1.4	-1.4	-1.6	-1.4
Spats2l	-1.5	-1.4	-1.5	-1.4
Fam132a	-1.4	-1.4	-1.6	-1.4
Vim	-1.3	-1.5	-1.5	-1.4
Man2a1	-1.5	-1.4	-1.5	-1.4
F2r	-1.3	-1.3	-1.7	-1.4
Mt1	-1.6	-1.5	-1.2	-1.4
Dclk2	-1.5	-1.4	-1.4	-1.4
Cxcl12	-1.5	-1.5	-1.4	-1.4
Sulf1	-1.7	-1.2	-1.4	-1.4
Nrp2	-1.3	-1.3	-1.7	-1.4
Pycard	-1.2	-1.3	-1.8	-1.4
A_55_P2013273	-1.2	-1.4	-1.7	-1.4
Afm	-1.5	-1.4	-1.5	-1.4
ENSMUST00000055719	-1.5	-1.4	-1.4	-1.4
Col3a1	-1.6	-1.4	-1.3	-1.4
Hand2	-1.3	-1.4	-1.5	-1.4
Zcchc12	-1.3	-1.5	-1.5	-1.4
Dpp4	-1.4	-1.5	-1.4	-1.4
Tfap2a	-1.7	-1.2	-1.4	-1.4
Adamts4	-1.3	-1.2	-1.8	-1.4
Ahnak	-1.4	-1.4	-1.5	-1.4
Evi2a	-1.4	-1.7	-1.3	-1.4
Tmem100	-1.3	-1.4	-1.5	-1.4
Fam3c	-1.2	-1.4	-1.7	-1.4
Prdm6	-1.3	-1.6	-1.3	-1.4
Hspb1	-1.2	-1.5	-1.6	-1.4
Mitf	-1.6	-1.3	-1.4	-1.4
Prcp	-1.6	-1.4	-1.3	-1.4
Casp12	-1.3	-1.4	-1.5	-1.4
Kcne1l	-1.2	-1.3	-1.7	-1.4
Tceal6	-1.3	-1.5	-1.5	-1.4
Nrn1l	-1.4	-1.3	-1.6	-1.4
A530047J11Rik	-1.4	-1.3	-1.5	-1.4
Fcer1g	-1.2	-1.4	-1.6	-1.4
Nrbp2	-1.3	-1.5	-1.4	-1.4
Ankrd44	-1.3	-1.6	-1.4	-1.4
Glt8d2	-1.3	-1.4	-1.6	-1.4
Col12a1	-1.5	-1.3	-1.4	-1.4
Tomm7	-1.5	-1.3	-1.5	-1.4
Nme5	-1.6	-1.4	-1.2	-1.4
Rs5-8s1	-1.2	-1.4	-1.6	-1.4
Ndrg2	-1.4	-1.4	-1.4	-1.4
Tub	-1.4	-1.4	-1.5	-1.4
Dnajb4	-1.4	-1.3	-1.5	-1.4
Emp2	-1.5	-1.3	-1.4	-1.4
Ankrd29	-1.4	-1.3	-1.5	-1.4
Fn1	-1.4	-1.4	-1.4	-1.4
Foxp2	-1.5	-1.4	-1.3	-1.4
Tmem213	-1.3	-1.3	-1.6	-1.4
1500015A07Rik	-1.5	-1.2	-1.5	-1.4
Ncf4	-1.4	-1.2	-1.5	-1.4
NAP005064-002	-1.2	-1.4	-1.6	-1.4
Klf2	-1.4	-1.4	-1.4	-1.4
Lifr	-1.6	-1.4	-1.2	-1.4
Car3	-1.4	-1.3	-1.5	-1.4
Ptger4	-1.4	-1.3	-1.5	-1.4
Gdpd2	-1.7	-1.3	-1.2	-1.4
Prrx2	-1.2	-1.5	-1.5	-1.4
B230104C14Rik	-1.7	-1.2	-1.3	-1.4
Plcxd2	-1.3	-1.4	-1.5	-1.4
Gng11	-1.3	-1.3	-1.6	-1.4
Lrrc8c	-1.4	-1.2	-1.5	-1.4
Mylk	-1.4	-1.2	-1.5	-1.4
Cap2	-1.5	-1.3	-1.4	-1.4
Nexn	-1.4	-1.3	-1.4	-1.4
Elavl2	-1.6	-1.3	-1.2	-1.4
Dsc2	-1.4	-1.3	-1.5	-1.4
Adcyap1r1	-1.4	-1.2	-1.5	-1.4
Rerg	-1.3	-1.3	-1.5	-1.4
Itga6	-1.3	-1.2	-1.6	-1.4
Dusp6	-1.3	-1.2	-1.7	-1.4
5730416F02Rik	-1.3	-1.3	-1.5	-1.4
Lmo3	-1.5	-1.2	-1.4	-1.4
Nfatc2	-1.5	-1.3	-1.3	-1.4
Pamr1	-1.5	-1.2	-1.4	-1.4
Col23a1	-1.5	-1.4	-1.3	-1.4
Tfcp2l1	-1.5	-1.4	-1.2	-1.4
Npb	-1.3	-1.4	-1.5	-1.4
Tceal3	-1.3	-1.4	-1.5	-1.4
Ramp3	-1.4	-1.2	-1.5	-1.4
Hbb-b1	-1.3	-1.3	-1.5	-1.4
Bnc2	-1.5	-1.3	-1.3	-1.4

Gene symbol	FC 1	FC 2	FC 3	Avg FC
Ldoc1	-1.6	-1.3	-1.3	-1.4
Rn28s1	-1.3	-1.4	-1.4	-1.4
Glrx	-1.4	-1.3	-1.4	-1.4
A_55_P1998854	-1.3	-1.5	-1.3	-1.4
Maoa	-1.5	-1.4	-1.2	-1.4
Slc29a1	-1.4	-1.3	-1.3	-1.4
Msrb3	-1.4	-1.3	-1.4	-1.4
Abcg2	-1.4	-1.3	-1.4	-1.4
LOC102642487	-1.3	-1.3	-1.5	-1.4
Dgkh	-1.4	-1.4	-1.3	-1.4
Fcgrt	-1.3	-1.3	-1.5	-1.4
Thbd	-1.4	-1.4	-1.3	-1.4
Lyl1	-1.3	-1.2	-1.6	-1.4
Stat4	-1.3	-1.3	-1.5	-1.4
Eogt	-1.3	-1.2	-1.5	-1.4
Adk	-1.3	-1.2	-1.5	-1.4
Clca2	-1.3	-1.2	-1.6	-1.4
Ppp1r3d	-1.4	-1.4	-1.3	-1.4
Tgfbi	-1.5	-1.3	-1.2	-1.4
Ppfibp2	-1.4	-1.2	-1.4	-1.4
Pcdh18	-1.4	-1.4	-1.3	-1.4
Rnft1	-1.3	-1.5	-1.3	-1.4
Clca1	-1.2	-1.3	-1.6	-1.3
B130024G19Rik	-1.3	-1.6	-1.2	-1.3
Atp10b	-1.4	-1.3	-1.3	-1.3
Wnt6	-1.5	-1.3	-1.3	-1.3
Sept4	-1.3	-1.4	-1.4	-1.3
Acvrl1	-1.5	-1.2	-1.3	-1.3
Irf5	-1.4	-1.2	-1.4	-1.3
Akr1c19	-1.3	-1.3	-1.5	-1.3
Galnt18	-1.4	-1.2	-1.5	-1.3
Nrp1	-1.3	-1.3	-1.4	-1.3
Isl1	-1.3	-1.2	-1.5	-1.3
Klhl29	-1.5	-1.3	-1.2	-1.3
Man1a	-1.3	-1.5	-1.2	-1.3
Dgat2	-1.4	-1.2	-1.3	-1.3
Ugt1a6b	-1.3	-1.4	-1.3	-1.3
Marveld1	-1.2	-1.6	-1.2	-1.3
Crip1	-1.2	-1.2	-1.6	-1.3
Trim34a	-1.2	-1.2	-1.5	-1.3
Cdh2	-1.4	-1.3	-1.3	-1.3
Epdr1	-1.3	-1.4	-1.3	-1.3
Trem2	-1.3	-1.3	-1.4	-1.3
Amph	-1.4	-1.3	-1.2	-1.3
Ncf2	-1.3	-1.3	-1.4	-1.3
Gm13889	-1.2	-1.2	-1.5	-1.3
Ssbp1	-1.3	-1.3	-1.4	-1.3
Pcdhac2	-1.4	-1.4	-1.2	-1.3
Magel2	-1.4	-1.2	-1.4	-1.3
Nab1	-1.2	-1.4	-1.3	-1.3
Clybl	-1.3	-1.3	-1.4	-1.3
Irf6	-1.4	-1.2	-1.4	-1.3
A230070E04Rik	-1.4	-1.2	-1.3	-1.3
Tmtc4	-1.3	-1.3	-1.3	-1.3
Ddc	-1.4	-1.3	-1.3	-1.3
Pgm5	-1.3	-1.3	-1.4	-1.3
Lrp2	-1.4	-1.3	-1.3	-1.3
A930033H14Rik	-1.3	-1.3	-1.3	-1.3
Cx3cr1	-1.4	-1.2	-1.3	-1.3
Sgce	-1.3	-1.2	-1.4	-1.3

Gene symbol	FC 1	FC 2	FC 3	Avg FC
Gxylt2	-1.3	-1.2	-1.4	-1.3
Cdv3	-1.3	-1.4	-1.2	-1.3
Adam8	-1.3	-1.3	-1.3	-1.3
Agpat2	-1.2	-1.2	-1.5	-1.3
Gm2016	-1.2	-1.3	-1.4	-1.3
Gsn	-1.4	-1.2	-1.3	-1.3
Col16a1	-1.4	-1.2	-1.3	-1.3
Wscd2	-1.3	-1.2	-1.4	-1.3
Airn	-1.2	-1.4	-1.3	-1.3
Clvs1	-1.4	-1.3	-1.2	-1.3
Spns2	-1.4	-1.3	-1.2	-1.3
Rbms3	-1.2	-1.4	-1.3	-1.3
Gm6598	-1.3	-1.2	-1.4	-1.3
Irf2bp2	-1.4	-1.3	-1.3	-1.3
Cnksr3	-1.3	-1.2	-1.4	-1.3
Arhgap36	-1.5	-1.2	-1.2	-1.3
Prkcb	-1.2	-1.4	-1.3	-1.3
Tnip2	-1.3	-1.3	-1.4	-1.3
Adcy1	-1.2	-1.2	-1.4	-1.3
2610035D17Rik	-1.3	-1.3	-1.3	-1.3
Isl2	-1.4	-1.2	-1.3	-1.3
Eif1a	-1.3	-1.2	-1.4	-1.3
Slc18a1	-1.3	-1.4	-1.2	-1.3
Col9a2	-1.3	-1.3	-1.3	-1.3
P4ha2	-1.3	-1.3	-1.3	-1.3
Snx16	-1.4	-1.2	-1.3	-1.3
Cdh13	-1.3	-1.2	-1.3	-1.3
Nfib	-1.3	-1.2	-1.3	-1.3
Gsta3	-1.3	-1.2	-1.3	-1.3
Ctsl	-1.3	-1.2	-1.3	-1.3
Cd9	-1.2	-1.4	-1.2	-1.3
Gria3	-1.3	-1.2	-1.3	-1.3
Stambpl1	-1.2	-1.3	-1.3	-1.3
Lpar4	-1.3	-1.2	-1.3	-1.3
Ckmt1	-1.4	-1.2	-1.2	-1.3
Gm9856	-1.3	-1.3	-1.2	-1.3
Prrg1	-1.3	-1.3	-1.2	-1.3
Btg1	-1.3	-1.3	-1.3	-1.3
Tiam2	-1.3	-1.2	-1.3	-1.3
Car13	-1.2	-1.2	-1.4	-1.3
Leprel1	-1.3	-1.3	-1.2	-1.3
Palm3	-1.3	-1.3	-1.2	-1.3
Emr1	-1.3	-1.3	-1.2	-1.3
Gm13238	-1.3	-1.3	-1.2	-1.3
Tmem9b	-1.2	-1.3	-1.3	-1.3
Anxa2	-1.2	-1.3	-1.3	-1.3
Lst1	-1.2	-1.2	-1.3	-1.3
Arap2	-1.3	-1.2	-1.3	-1.3
Hist3h2a	-1.2	-1.2	-1.3	-1.3
Ppap2c	-1.3	-1.3	-1.2	-1.3
Lhpp	-1.3	-1.2	-1.2	-1.3
Wnt11	-1.3	-1.2	-1.2	-1.2
Inf2	-1.2	-1.2	-1.3	-1.2
Has2	-1.2	-1.3	-1.2	-1.2
Hspb6	-1.3	-1.2	-1.2	-1.2
C030034I22Rik	-1.2	-1.3	-1.3	-1.2
Epcam	-1.2	-1.2	-1.3	-1.2
Zfp217	-1.2	-1.2	-1.2	-1.2
Tfpi	-1.2	-1.2	-1.2	-1.2
Rab17	-1.2	-1.2	-1.2	-1.2

Supplementary Table S8. Transcripts identified by microarray analysis that were negatively regulated by RA in the early ureter. Shown is a list of transcripts that were negatively regulated by RA, i.e. upregulated after BMS493 treatment and downregulated after RA treatment of E12.5 ureter explants. Three groups for each condition were compared to untreated controls and the resulting fold changes (FC) in expression are displayed. Intensity thresholds were >100; fold changes were larger than 1.2.

Rank	Gene symbol	BMS493 FC 1	BMS493 FC 2	BMS493 FC 3	BMS493 Avg FC	RA FC 1	RA FC 2	RA FC 3	RA Avg FC
1	S100a4	4.0	4.5	4.0	4.2	-3.7	-2.8	-5.0	-3.8
2	Krt6a	4.0	4.2	2.1	3.4	-2.9	-2.8	-4.9	-3.6
3	Rgcc	3.1	3.5	3.3	3.3	-2.6	-3.9	-6.1	-4.2
4	Serpine1	3.3	2.9	2.6	2.9	-2.8	-2.5	-7.3	-4.2
5	Ugt2b34	2.3	2.7	3.2	2.7	-4.3	-4.8	-5.0	-4.7
6	Dkk1	2.3	2.6	3.1	2.7	-4.3	-3.5	-4.4	-4.1
7	Kcnc2	2.5	2.9	2.5	2.7	-3.0	-2.0	-1.7	-2.2
8	Osr2	3.4	2.6	2.0	2.7	-2.1	-1.4	-2.2	-1.9
9	Ryr3	2.8	2.5	2.5	2.6	-1.6	-1.9	-2.3	-1.9
10	Wif1	2.5	2.8	2.3	2.5	-2.4	-2.4	-2.3	-2.4
11	Serpina3f	2.2	2.8	2.6	2.5	-1.9	-2.0	-4.5	-2.8
12	Penk	2.4	2.2	2.[1]9	2.5	-2.6	-3.2	-3.2	-3.0
13	Glis1	2.2	2.4	2.8	2.5	-2.1	-2.0	-1.7	-1.9
14	Aard	2.7	2.1	2.3	2.4	-2.2	-2.1	-2.6	-2.3
15	Serpina3i	2.4	2.7	1.9	2.3	-1.7	-1.7	-5.2	-2.8
16	Lypd3	2.6	2.3	2.0	2.3	-1.6	-1.4	-2.3	-1.8
17	Slc26a7	2.4	1.9	2.5	2.3	-2.6	-1.7	-2.0	-2.1
18	Grm7	2.0	2.7	2.1	2.3	-4.4	-3.7	-4.2	-4.1
19	Trim29	2.8	2.2	1.7	2.2	-2.2	-1.4	-3.1	-2.2
20	Nts	2.0	2.8	1.8	2.2	-3.4	-4.0	-9.5	-5.6
21	Gabra1	2.1	1.9	2.5	2.2	-5.3	-5.8	-5.9	-5.7
22	Npy	2.9	2.0	1.6	2.2	-5.4	-4.9	-5.4	-5.2
23	Vcam1	2.1	2.2	2.0	2.1	-2.8	-3.0	-3.1	-3.0
24	Krt5	2.4	2.2	1.7	2.1	-2.1	-1.9	-3.7	-2.5
25	Rasd2	1.9	2.6	1.7	2.1	-3.1	-2.5	-2.5	-2.7
26	Cd55	1.8	2.0	2.4	2.1	-1.7	-2.1	-1.4	-1.7
27	Gem	2.0	2.1	2.0	2.0	-1.8	-1.5	-2.0	-1.8
28	Kcnab1	2.0	1.9	2.1	2.0	-2.3	-1.8	-2.8	-2.3
29	Ltbp2	2.0	2.3	1.8	2.0	-2.4	-2.2	-4.6	-3.1
30	Emp2	1.5	2.8	1.8	2.0	-1.5	-1.3	-1.4	-1.4
31	Mt2	1.9	2.0	2.1	2.0	-1.8	-1.9	-1.6	-1.7
32	Artn	1.9	2.1	2.0	2.0	-1.8	-1.5	-1.8	-1.7
33	Dmrta2	1.7	2.0	2.2	2.0	-1.6	-1.5	-1.3	-1.5
34	Daf2	2.0	1.9	2.0	2.0	-1.8	-1.9	-1.7	-1.8
35	Anxa8	2.1	2.1	1.7	2.0	-2.4	-2.2	-3.7	-2.8
36	Svep1	1.8	2.1	1.8	1.9	-2.0	-1.4	-1.7	-1.7
37	Kcnip4	2.0	1.9	1.9	1.9	-1.8	-2.7	-3.1	-2.5
38	Hmcn1	2.1	2.0	1.7	1.9	-1.9	-1.6	-1.7	-1.8
39	Isl2	1.7	1.7	2.4	1.9	-1.4	-1.2	-1.3	-1.3
40	Tox	1.9	1.9	1.9	1.9	-3.1	-2.4	-3.1	-2.9
41	S100a6	2.1	2.0	1.5	1.9	-1.7	-1.7	-3.0	-2.1
42	Tfap2a	1.8	2.6	1.3	1.9	-1.7	-1.2	-1.4	-1.4
43	Rasgef1a	1.4	2.2	2.0	1.9	-2.4	-1.9	-2.1	-2.1
44	Pcdh10	2.2	1.9	1.6	1.9	-1.7	-1.6	-2.1	-1.8
45	Nfatc2	1.6	2.1	1.9	1.9	-1.5	-1.3	-1.3	-1.4
46	Cx3cl1	1.8	1.8	1.9	1.9	-1.5	-1.3	-3.1	-1.9
47	Aqp3	1.7	1.6	2.2	1.8	-2.8	-2.7	-1.9	-2.5
48	Zfp365	1.8	1.8	1.9	1.8	-2.6	-2.2	-2.2	-2.3
49	Dlk1	2.3	1.9	1.3	1.8	-1.7	-1.4	-2.7	-1.9
50	Vnn1	1.4	1.9	2.1	1.8	-1.8	-1.8	-1.7	-1.8
51	Ptchd4	1.5	2.2	1.7	1.8	-1.7	-1.5	-2.0	-1.8
52	Robo2	1.7	1.8	1.9	1.8	-2.2	-2.1	-1.5	-1.9
53	Tfap2b	2.0	2.0	1.3	1.8	-2.4	-2.4	-2.9	-2.6
54	Gnas	1.8	2.1	1.5	1.8	-2.8	-3.6	-3.5	-3.3
55	Rgs5	1.7	1.8	1.9	1.8	-3.3	-5.5	-5.5	-4.8
56	Rgs4	1.6	1.8	1.9	1.8	-4.1	-3.5	-5.2	-4.3
57	Ctgf	1.7	1.9	1.8	1.8	-3.0	-3.1	-5.1	-3.7
58	Lrrc75b	1.7	2.1	1.6	1.8	-2.5	-2.4	-2.6	-2.5

Rank	Gene symbol	BMS493 FC 1	BMS493 FC 2	BMS493 FC 3	BMS493 Avg FC	RA FC 1	RA FC 2	RA FC 3	RA Avg FC
59	Figf	1.9	2.1	1.3	1.8	-2.1	-2.1	-2.8	-2.3
60	Rab38	1.7	1.9	1.8	1.8	-1.7	-2.1	-3.1	-2.3
61	Perp	1.9	1.8	1.7	1.8	-2.0	-1.5	-3.0	-2.2
62	Six2	1.6	1.7	2.0	1.8	-2.3	-1.4	-1.9	-1.9
63	Sytl2	2.0	1.5	1.8	1.8	-1.9	-1.6	-1.9	-1.8
64	Aldh1a2	1.8	1.8	1.6	1.7	-2.3	-1.8	-1.8	-2.0
65	Ndrg1	1.9	1.9	1.5	1.7	-1.6	-1.2	-1.7	-1.5
66	Kcnq5	1.9	1.9	1.5	1.7	-2.1	-1.4	-1.8	-1.8
67	Zmat4	1.7	1.9	1.7	1.7	-1.6	-1.3	-2.4	-1.8
68	Tmem132c	1.6	1.8	1.8	1.7	-2.0	-1.6	-1.5	-1.7
69	Egr2	1.6	1.7	2.0	1.7	-2.6	-2.4	-5.3	-3.4
70	Gm11783	1.5	2.1	1.6	1.7	-2.3	-3.9	-3.6	-3.3
71	Gabrb2	1.8	2.0	1.4	1.7	-3.0	-2.3	-3.1	-2.8
72	Wnt6	1.6	1.6	2.0	1.7	-1.5	-1.3	-1.3	-1.3
73	Rarres1	1.8	1.5	1.8	1.7	-1.6	-1.6	-2.2	-1.8
74	Pip5k1a	1.6	1.8	1.7	1.7	-1.5	-1.4	-1.5	-1.5
75	Dclk2	2.0	1.7	1.5	1.7	-1.5	-1.4	-1.4	-1.4
76	Plekha4	1.7	1.8	1.7	1.7	-1.9	-1.9	-2.4	-2.1
77	Pitx2	1.6	1.8	1.7	1.7	-2.0	-1.5	-1.8	-1.8
78	Stc1	1.8	1.6	1.7	1.7	-1.7	-2.2	-1.8	-1.9
79	Trim47	1.6	1.7	1.7	1.7	-1.8	-1.6	-2.9	-2.1
80	Ctsc	1.7	2.0	1.4	1.7	-1.6	-1.5	-2.3	-1.8
81	Dkk2	1.5	1.8	1.7	1.7	-3.0	-3.4	-4.5	-3.6
82	Cyr61	1.6	1.8	1.7	1.7	-1.9	-1.7	-2.5	-2.0
83	Bgn	2.0	1.6	1.4	1.7	-1.5	-1.5	-2.4	-1.8
84	Ddah1	1.6	1.8	1.6	1.7	-1.3	-1.4	-2.1	-1.6
85	Epha3	1.5	1.5	1.9	1.7	-2.4	-2.8	-2.4	-2.5
86	Nr4a1	1.8	1.7	1.5	1.7	-1.7	-1.5	-2.5	-1.9
87	Actg2	1.6	1.8	1.5	1.6	-2.1	-1.7	-2.9	-2.2
88	Nfkbiz	1.7	2.0	1.3	1.6	-1.9	-1.5	-2.0	-1.8
89	Prrx2	1.5	1.6	1.8	1.6	-1.2	-1.5	-1.5	-1.4
90	Agtr1a	1.6	1.7	1.5	1.6	-1.6	-1.5	-1.6	-1.6
91	Fgf12	1.8	1.7	1.4	1.6	-1.6	-1.8	-1.4	-1.6
92	March11	1.5	1.7	1.6	1.6	-1.4	-1.7	-2.2	-1.8
93	Sfmbt2	1.3	1.6	1.9	1.6	-1.6	-1.7	-1.4	-1.6
94	Lgi2	1.7	1.5	1.6	1.6	-1.4	-1.9	-1.5	-1.6
95	Btc	1.8	1.6	1.4	1.6	-2.0	-1.8	-1.9	-1.9
96	Slc23a3	1.4	1.5	2.0	1.6	-1.9	-1.9	-1.6	-1.8
97	Hopx	2.1	1.4	1.3	1.6	-1.7	-1.7	-2.2	-1.9
98	Fam150b	1.7	1.8	1.3	1.6	-2.8	-3.1	-7.8	-4.6
99	Hist2h2bb	1.6	1.8	1.4	1.6	-1.7	-1.4	-1.3	-1.5
100	Ace2	1.7	1.7	1.4	1.6	-6.1	-5.1	-4.9	-5.4
101	Tnc	1.7	1.5	1.6	1.6	-1.4	-1.7	-1.6	-1.6
102	Gas2	1.4	1.5	1.8	1.6	-1.6	-1.8	-2.1	-1.8
103	Kcnn2	1.7	1.7	1.3	1.6	-1.9	-1.6	-1.8	-1.8
104	Lhfpl3	1.9	1.4	1.5	1.6	-1.3	-2.2	-1.9	-1.8
105	Itga11	1.4	1.7	1.6	1.6	-2.2	-1.3	-2.1	-1.9
106	Ptchd1	1.6	1.6	1.5	1.6	-2.1	-2.3	-2.2	-2.2
107	Cd44	1.7	1.7	1.3	1.6	-1.5	-1.9	-2.4	-1.9
108	Ppp1r3d	1.5	1.6	1.7	1.6	-1.4	-1.4	-1.3	-1.4
109	Gfra2	1.7	1.6	1.4	1.6	-2.2	-2.3	-1.9	-2.1
110	Pdgfrb	1.6	1.6	1.4	1.6	-2.3	-1.8	-1.9	-2.0
111	Fas	1.4	1.7	1.5	1.6	-1.9	-2.2	-4.2	-2.7
112	Hand1	1.9	1.5	1.4	1.6	-1.5	-2.0	-2.5	-2.0
113	Nrp2	1.6	1.9	1.2	1.6	-1.3	-1.3	-1.7	-1.4
114	Ntrk3	1.7	1.7	1.3	1.6	-1.7	-1.4	-2.2	-1.8
115	Gpc4	1.5	1.7	1.5	1.6	-1.5	-1.2	-2.0	-1.6
116	Rasgrp2	1.7	1.5	1.4	1.6	-1.5	-1.3	-2.0	-1.6
117	Npas4	1.6	1.5	1.5	1.6	-1.5	-1.4	-1.7	-1.5
118	Lmo1	1.6	1.6	1.5	1.6	-2.2	-2.8	-3.0	-2.6
119	Lrrtm1	1.6	1.7	1.4	1.6	-2.1	-2.2	-2.6	-2.3
120	Tiam2	1.5	1.7	1.5	1.5	-1.3	-1.2	-1.3	-1.3
121	Tmem26	1.6	1.6	1.5	1.5	-1.6	-1.9	-1.7	-1.7
122	Frzb	1.5	1.6	1.5	1.5	-2.0	-1.7	-2.1	-1.9
123	Tmem40	1.6	1.5	1.5	1.5	-1.4	-1.4	-2.0	-1.6
124	Col12a1	1.5	1.5	1.6	1.5	-1.5	-1.3	-1.4	-1.4
125	Mamdc2	1.6	1.3	1.7	1.5	-2.2	-1.8	-2.2	-2.1

Rank	Gene symbol	BMS493 FC 1	BMS493 FC 2	BMS493 FC 3	BMS493 Avg FC	RA FC 1	RA FC 2	RA FC 3	RA Avg FC
126	1810011O10Rik	1.5	1.4	1.7	1.5	-1.5	-1.7	-2.4	-1.9
127	Pcdh8	1.5	1.8	1.3	1.5	-2.2	-2.6	-2.2	-2.3
128	Arid5a	1.5	1.6	1.5	1.5	-1.5	-1.4	-1.7	-1.5
129	Ssfa2	1.7	1.5	1.3	1.5	-1.7	-1.5	-1.7	-1.6
130	Lum	1.5	1.4	1.6	1.5	-2.7	-3.5	-3.2	-3.1
131	Itga4	1.5	1.6	1.5	1.5	-1.6	-1.5	-1.5	-1.5
132	Ism1	1.5	1.6	1.4	1.5	-1.9	-1.5	-1.6	-1.7
133	Fos	1.4	1.5	1.6	1.5	-1.6	-1.8	-2.4	-1.9
134	Gadd45b	1.6	1.5	1.5	1.5	-1.2	-1.6	-2.3	-1.7
135	Magel2	1.6	1.3	1.6	1.5	-1.4	-1.2	-1.4	-1.3
136	S100a13	1.4	1.6	1.6	1.5	-1.4	-1.4	-1.6	-1.5
137	5330426P16Rik	1.4	1.4	1.6	1.5	-1.5	-1.5	-1.5	-1.5
138	NAP061760-1	1.8	1.4	1.3	1.5	-1.2	-1.2	-2.4	-1.6
139	Jag1	1.5	1.5	1.4	1.5	-1.5	-1.4	-1.6	-1.5
140	Dgkk	1.4	1.7	1.4	1.5	-1.8	-2.7	-1.6	-2.0
141	Pamr1	1.4	1.7	1.5	1.5	-1.5	-1.2	-1.4	-1.4
142	Ccdc3	1.5	1.7	1.3	1.5	-1.7	-1.5	-1.7	-1.7
143	Maob	1.3	1.5	1.7	1.5	-2.1	-2.2	-2.2	-2.2
144	Cryab	1.9	1.3	1.3	1.5	-1.4	-1.7	-2.1	-1.7
145	Acta1	1.7	1.4	1.4	1.5	-1.2	-1.3	-2.7	-1.8
146	Cap2	1.7	1.3	1.5	1.5	-1.5	-1.3	-1.4	-1.4
147	Fxyd2	1.2	1.4	1.8	1.5	-1.4	-1.7	-1.6	-1.6
148	Kctd12	1.5	1.4	1.5	1.5	-2.0	-2.1	-1.3	-1.8
149	Fbn2	1.6	1.7	1.2	1.5	-1.5	-1.6	-1.4	-1.5
150	Hspb1	1.5	1.2	1.6	1.5	-1.2	-1.5	-1.6	-1.4
151	Fn1	1.5	1.3	1.6	1.5	-1.4	-1.4	-1.4	-1.4
152	Mme	1.6	1.5	1.4	1.5	-2.1	-1.5	-1.5	-1.7
153	C1qtnf2	1.8	1.2	1.4	1.5	-1.4	-1.6	-1.7	-1.6
154	Tmem100	1.5	1.5	1.4	1.5	-1.3	-1.4	-1.5	-1.4
155	Pmaip1	1.4	1.6	1.4	1.5	-1.4	-1.4	-2.9	-1.9
156	Traf1	1.4	1.6	1.4	1.5	-1.4	-1.3	-1.6	-1.5
157	1500009L16Rik	1.4	1.4	1.6	1.5	-1.3	-1.5	-1.7	-1.5
158	Rasgef1b	1.4	1.5	1.5	1.5	-1.9	-1.7	-1.7	-1.8
159	Gsn	1.4	1.5	1.4	1.5	-1.4	-1.2	-1.3	-1.3
160	Loxl1	1.7	1.4	1.2	1.5	-1.5	-1.3	-1.9	-1.6
161	Mef2c	1.5	1.6	1.3	1.4	-1.2	-1.6	-1.7	-1.5
162	Ier3	1.3	1.3	1.7	1.4	-1.6	-1.5	-2.5	-1.9
163	Prdm6	1.5	1.4	1.5	1.4	-1.3	-1.6	-1.3	-1.4
164	2610018G03Rik	1.7	1.2	1.5	1.4	-1.4	-1.9	-1.6	-1.6
165	Ptn	1.6	1.4	1.4	1.4	-2.3	-2.3	-2.3	-2.3
166	Adamts14	1.4	1.5	1.4	1.4	-1.4	-1.4	-1.8	-1.5
167	Lxn	1.2	1.6	1.5	1.4	-1.4	-1.4	-1.5	-1.5
168	Grem2	1.5	1.5	1.4	1.4	-2.3	-2.0	-1.9	-2.1
169	Wnt11	1.2	1.4	1.7	1.4	-1.3	-1.2	-1.2	-1.2
170	Lilrb4	1.5	1.3	1.5	1.4	-1.8	-1.6	-1.7	-1.7
171	Id4	1.5	1.5	1.3	1.4	-1.6	-1.5	-1.7	-1.6
172	Vav3	1.4	1.5	1.4	1.4	-1.8	-1.4	-1.9	-1.7
173	Sqrdl	1.4	1.6	1.3	1.4	-1.4	-1.5	-2.0	-1.6
174	Actc1	1.5	1.4	1.5	1.4	-2.0	-2.2	-2.5	-2.2
175	Rdh10	1.4	1.3	1.6	1.4	-1.8	-1.5	-1.7	-1.7
176	Npb	1.2	1.5	1.6	1.4	-1.3	-1.4	-1.5	-1.4
177	Chst1	1.4	1.5	1.4	1.4	-1.6	-1.5	-1.5	-1.5
178	Dpp4	1.3	1.5	1.5	1.4	-1.4	-1.5	-1.4	-1.4
179	Smoc2	1.6	1.3	1.4	1.4	-1.7	-1.6	-1.8	-1.7
180	Egr1	1.3	1.5	1.5	1.4	-2.0	-1.7	-2.0	-1.9
181	Il1r1	1.5	1.6	1.3	1.4	-1.6	-1.3	-2.1	-1.6
182	Lox	1.4	1.5	1.4	1.4	-2.5	-1.9	-1.9	-2.1
183	Grin3a	1.4	1.4	1.5	1.4	-1.3	-1.5	-1.6	-1.5
184	March3	1.6	1.4	1.3	1.4	-1.3	-1.3	-2.0	-1.5
185	Lrrn3	1.4	1.3	1.6	1.4	-2.1	-2.2	-1.9	-2.1
186	Runx1t1	1.4	1.4	1.5	1.4	-1.6	-1.8	-1.3	-1.6
187	Degs2	1.5	1.3	1.5	1.4	-1.4	-1.5	-1.7	-1.5
188	Pdlim2	1.7	1.3	1.3	1.4	-1.4	-1.5	-1.7	-1.6
189	Akr1c19	1.5	1.5	1.3	1.4	-1.3	-1.3	-1.5	-1.3
190	Palm3	1.3	1.4	1.5	1.4	-1.3	-1.3	-1.2	-1.3
191	Lmna	1.6	1.4	1.2	1.4	-1.4	-1.4	-1.9	-1.6
192	Ramp2	1.5	1.4	1.3	1.4	-1.5	-1.5	-1.8	-1.6

Rank	Gene symbol	BMS493 FC 1	BMS493 FC 2	BMS493 FC 3	BMS493 Avg FC	RA FC 1	RA FC 2	RA FC 3	RA Avg FC
193	Cav1	1.6	1.4	1.2	1.4	-1.7	-1.6	-2.9	-2.1
194	Fbln2	1.4	1.6	1.2	1.4	-1.8	-2.0	-1.9	-1.9
195	9330159M07Rik	1.2	1.3	1.7	1.4	-1.3	-1.6	-1.5	-1.5
196	Ldoc1	1.2	1.4	1.6	1.4	-1.6	-1.3	-1.3	-1.4
197	Ckmt1	1.2	1.4	1.5	1.4	-1.4	-1.2	-1.2	-1.3
198	Nup62cl	1.4	1.4	1.4	1.4	-1.8	-1.3	-2.4	-1.8
199	Tacstd2	1.5	1.4	1.3	1.4	-1.6	-1.3	-1.7	-1.5
200	Nrbp2	1.4	1.4	1.3	1.4	-1.3	-1.5	-1.4	-1.4
201	1700011H14Rik	1.2	1.6	1.4	1.4	-1.4	-1.4	-1.6	-1.5
202	Mmd	1.3	1.4	1.4	1.4	-1.4	-1.5	-1.6	-1.5
203	Thbs1	1.4	1.4	1.3	1.4	-1.5	-1.4	-2.0	-1.7
204	Galnt18	1.6	1.2	1.3	1.4	-1.4	-1.2	-1.5	-1.3
205	Trps1	1.3	1.5	1.3	1.4	-1.5	-1.7	-1.3	-1.5
206	Fbln5	1.4	1.5	1.3	1.4	-2.1	-2.2	-2.0	-2.1
207	Fgd3	1.4	1.5	1.3	1.4	-1.4	-1.3	-2.2	-1.6
208	Prr5l	1.3	1.4	1.4	1.4	-1.4	-1.4	-1.5	-1.5
209	Klf2	1.2	1.4	1.5	1.4	-1.4	-1.4	-1.4	-1.4
210	Rspo3	1.3	1.3	1.5	1.4	-1.8	-1.9	-1.9	-1.9
211	Ahnak	1.4	1.2	1.5	1.4	-1.4	-1.4	-1.5	-1.4
212	Spry2	1.4	1.3	1.4	1.4	-1.3	-1.3	-2.0	-1.6
213	Bmp5	1.3	1.6	1.3	1.4	-1.5	-1.6	-1.5	-1.6
214	Bai3	1.4	1.4	1.3	1.4	-1.7	-1.4	-1.5	-1.6
215	Cav2	1.3	1.3	1.4	1.4	-2.0	-1.9	-2.7	-2.2
216	Pdzk1ip1	1.3	1.2	1.5	1.4	-1.5	-1.6	-1.7	-1.6
217	Pnma2	1.3	1.5	1.2	1.4	-1.9	-1.6	-1.7	-1.7
218	Fxyd5	1.5	1.3	1.2	1.4	-1.3	-1.3	-2.2	-1.6
219	Anxa1	1.4	1.2	1.4	1.4	-1.5	-1.8	-2.6	-2.0
220	Arid5b	1.3	1.4	1.3	1.4	-1.8	-1.3	-1.4	-1.5
221	Rnd3	1.2	1.3	1.5	1.3	-2.1	-1.5	-1.5	-1.7
222	Inf2	1.3	1.4	1.3	1.3	-1.2	-1.2	-1.3	-1.2
223	Filip1l	1.5	1.3	1.3	1.3	-1.8	-1.9	-2.3	-2.0
224	Sh3tc2	1.4	1.4	1.2	1.3	-1.5	-1.4	-1.6	-1.5
225	Tfcp2l1	1.3	1.3	1.4	1.3	-1.5	-1.4	-1.2	-1.4
226	Dgkh	1.3	1.3	1.4	1.3	-1.4	-1.4	-1.3	-1.4
227	Lgals3	1.3	1.4	1.2	1.3	-1.8	-1.5	-1.8	-1.7
228	Emb	1.4	1.3	1.3	1.3	-1.5	-1.5	-1.3	-1.5
229	Tagln	1.3	1.3	1.4	1.3	-1.7	-1.6	-2.7	-2.0
230	Man2a1	1.2	1.5	1.3	1.3	-1.5	-1.4	-1.5	-1.4
231	Pcdh19	1.3	1.3	1.4	1.3	-1.7	-1.6	-1.4	-1.6
232	1110006E14Rik	1.3	1.5	1.2	1.3	-1.9	-1.5	-1.5	-1.6
233	Fam13c	1.3	1.4	1.3	1.3	-1.8	-1.6	-1.5	-1.6
234	Slamf9	1.3	1.5	1.2	1.3	-1.4	-1.4	-2.2	-1.6
235	Mt1	1.3	1.3	1.4	1.3	-1.6	-1.5	-1.2	-1.4
236	Gxylt2	1.3	1.4	1.3	1.3	-1.3	-1.2	-1.4	-1.3
237	Gng11	1.3	1.4	1.2	1.3	-1.3	-1.3	-1.6	-1.4
238	Phactr1	1.3	1.3	1.3	1.3	-1.6	-1.6	-1.6	-1.6
239	Plet1	1.3	1.3	1.3	1.3	-1.8	-2.0	-2.2	-2.0
240	Adk	1.3	1.4	1.2	1.3	-1.3	-1.2	-1.5	-1.4
241	Itga8	1.3	1.3	1.3	1.3	-1.8	-1.4	-1.2	-1.5
242	Arl4a	1.3	1.3	1.4	1.3	-1.3	-1.4	-1.8	-1.5
243	Maff	1.2	1.3	1.3	1.3	-1.3	-1.3	-2.1	-1.6
244	Lhfpl1	1.4	1.2	1.3	1.3	-1.6	-1.5	-2.0	-1.7
245	Timp3	1.2	1.4	1.3	1.3	-1.7	-2.0	-2.1	-1.9
246	Lifr	1.3	1.2	1.4	1.3	-1.6	-1.4	-1.2	-1.4
247	Timp1	1.3	1.4	1.2	1.3	-1.3	-1.2	-2.4	-1.6
248	Ankrd29	1.3	1.3	1.2	1.3	-1.4	-1.3	-1.5	-1.4
249	8430408G22Rik	1.3	1.2	1.3	1.3	-1.5	-1.3	-1.7	-1.5
250	Plcxd3	1.2	1.3	1.3	1.3	-1.7	-2.6	-2.1	-2.1
251	Plau	1.3	1.3	1.2	1.3	-2.1	-2.4	-2.9	-2.5
252	Srpx	1.2	1.3	1.3	1.3	-1.3	-1.6	-1.7	-1.5
253	Clec4d	1.3	1.2	1.3	1.3	-1.6	-1.5	-2.1	-1.7
254	Dgat2	1.2	1.3	1.3	1.3	-1.4	-1.2	-1.3	-1.3
255	LOC102642487	1.3	1.2	1.3	1.3	-1.3	-1.3	-1.5	-1.4
256	Tnfaip3	1.2	1.3	1.2	1.2	-1.7	-1.5	-2.1	-1.8
257	Ptx3	1.3	1.2	1.2	1.2	-1.4	-1.7	-1.6	-1.6
258	Acvrl1	1.3	1.2	1.2	1.2	-1.5	-1.2	-1.3	-1.3
259	Lrp2	1.2	1.2	1.3	1.2	-1.4	-1.3	-1.3	-1.3

Rank	Gene symbol	BMS493 FC 1	BMS493 FC 2	BMS493 FC 3	BMS493 Avg FC	RA FC 1	RA FC 2	RA FC 3	RA Avg FC
260	Adamtsl1	1.2	1.3	1.2	1.2	-1.9	-1.5	-1.3	-1.6
261	Dnajb4	1.2	1.2	1.2	1.2	-1.4	-1.3	-1.5	-1.4

Supplementary Table S9. Functional enrichment analysis for transcripts that were negatively regulated by RA in the early ureter. Functional enrichment analysis for 261 genes was performed with DAVID websoftware (https://david.ncifcrf.gov) using default settings. Shown are enriched terms for the annotation categories/databases GO:biological process and GO:molecular function with a p-value p<0.05.

Term	PValue	Enrichment FC	Bonferroni	Benjamini	FDR
GO:0010628~positive regulation of gene expression	9.25E-10	4.86E+00	1.62E-06	1.62E-06	1.57E-06
GO:0007507~heart development	1.61E-07	5.26E+00	2.81E-04	1.41E-04	2.72E-04
GO:0042060~wound healing	2.41E-06	8.59E+00	4.20E-03	1.40E-03	4.08E-03
GO:0007155~cell adhesion	2.59E-06	3.50E+00	4.51E-03	1.13E-03	4.38E-03
GO:0008201~heparin binding	3.42E-06	6.27E+00	1.45E-03	1.45E-03	4.82E-03
GO:0001822~kidney development	4.88E-06	6.83E+00	8.49E-03	1.70E-03	8.26E-03
GO:0005509~calcium ion binding	8.69E-06	2.82E+00	3.69E-03	1.84E-03	1.22E-02
GO:0008284~positive regulation of cell proliferation	1.36E-05	3.13E+00	2.35E-02	3.95E-03	2.30E-02
GO:0007160~cell-matrix adhesion	4.96E-05	8.28E+00	8.31E-02	1.23E-02	8.40E-02
GO:0035914~skeletal muscle cell differentiation	5.76E-05	1.03E+01	9.57E-02	1.25E-02	9.74E-02
GO:0045471~response to ethanol	1.02E-04	6.21E+00	1.63E-01	1.95E-02	1.72E-01
GO:0016477~cell migration	1.34E-04	4.65E+00	2.08E-01	2.31E-02	2.26E-01
GO:0001974~blood vessel remodeling	1.79E-04	1.13E+01	2.69E-01	2.81E-02	3.03E-01
GO:0045669~positive regulation of osteoblast differentiation	1.90E-04	8.31E+00	2.83E-01	2.73E-02	3.22E-01
GO:0001525~angiogenesis	1.95E-04	4.05E+00	2.89E-01	2.59E-02	3.30E-01
GO:0060710~chorio-allantoic fusion	2.08E-04	3.23E+01	3.05E-01	2.57E-02	3.52E-01
GO:0005515~protein binding	2.10E-04	1.47E+00	8.53E-02	2.93E-02	2.95E-01
GO:0032355~response to estradiol	2.54E-04	6.39E+00	3.59E-01	2.92E-02	4.30E-01
GO:0005178~integrin binding	2.73E-04	6.32E+00	1.10E-01	2.86E-02	3.84E-01
GO:0001937~negative regulation of endothelial cell proliferation	4.80E-04	1.35E+01	5.68E-01	5.10E-02	8.09E-01
GO:0002020~protease binding	7.07E-04	5.40E+00	2.60E-01	5.84E-02	9.91E-01
GO:0045766~positive regulation of angiogenesis	7.59E-04	5.34E+00	7.35E-01	7.51E-02	1.28E+00
GO:0030335~positive regulation of cell migration	9.61E-04	3.98E+00	8.14E-01	8.91E-02	1.61E+00
GO:0001656~metanephros development	1.20E-03	1.06E+01	8.76E-01	1.04E-01	2.01E+00
GO:0016055~Wnt signaling pathway	1.34E-03	3.79E+00	9.05E-01	1.11E-01	2.25E+00
GO:0007568~aging	1.40E-03	4.20E+00	9.13E-01	1.10E-01	2.34E+00
GO:0070836~caveola assembly	1.48E-03	4.84E+01	9.24E-01	1.11E-01	2.47E+00
GO:0090131~mesenchyme migration	1.48E-03	4.84E+01	9.24E-01	1.11E-01	2.47E+00
GO:0090090~negative regulation of canonical Wnt signaling	1.66E-03	5.54E+00	9.45E-01	1.18E-01	2.77E+00
GO:0010811~positive regulation of cell-substrate adhesion	1.75E-03	9.61E+00	9.53E-01	1.19E-01	2.91E+00
GO:0043066~negative regulation of apoptotic process	1.82E-03	2.42E+00	9.58E-01	1.19E-01	3.03E+00
GO:0042127~regulation of cell proliferation	2.08E-03	3.56E+00	9.74E-01	1.31E-01	3.46E+00
GO:0090280~positive regulation of calcium ion import	2.09E-03	1.54E+01	9.74E-01	1.27E-01	3.48E+00
GO:0051412~response to corticosterone	2.09E-03	1.54E+01	9.74E-01	1.27E-01	3.48E+00
GO:0045893~positive regulation of transcription, DNA-templated	2.17E-03	2.38E+00	9.77E-01	1.27E-01	3.61E+00
GO:0072201~negative regulation of mesenchymal cell proliferation	2.20E-03	4.04E+01	9.79E-01	1.24E-01	3.66E+00
GO:0005539~glycosaminoglycan binding	2.22E-03	1.50E+01	6.12E-01	1.46E-01	3.09E+00
GO:0001077~transcriptional activator activity, RNA polymerase II	2.29E-03	3.22E+00	6.22E-01	1.30E-01	3.17E+00
GO:0035924~cellular response to vascular endothelial growth	2.40E-03	1.47E+01	9.85E-01	1.30E-01	3.98E+00
GO:0002062~chondrocyte differentiation	2.45E-03	8.77E+00	9.86E-01	1.29E-01	4.06E+00
GO:0001666~response to hypoxia	2.69E-03	3.78E+00	9.91E-01	1.37E-01	4.45E+00
GO:0045944~positive regulation of transcription from RNA	2.84E-03	1.95E+00	9.93E-01	1.40E-01	4.70E+00
GO:0043565~sequence-specific DNA binding	2.84E-03	2.24E+00	7.02E-01	1.40E-01	3.93E+00
GO:0044212~transcription regulatory region DNA binding	2.87E-03	3.39E+00	7.05E-01	1.27E-01	3.97E+00
GO:0045444~fat cell differentiation	2.93E-03	6.13E+00	9.94E-01	1.40E-01	4.85E+00
GO:0002544~chronic inflammatory response	3.05E-03	3.46E+01	9.95E-01	1.42E-01	5.04E+00
GO:0043065~positive regulation of apoptotic process	3.07E-03	2.89E+00	9.95E-01	1.38E-01	5.06E+00
GO:0034097~response to cytokine	3.27E-03	5.98E+00	9.97E-01	1.43E-01	5.39E+00
GO:0051216~cartilage development	3.45E-03	5.91E+00	9.98E-01	1.47E-01	5.68E+00
GO:0007275~multicellular organism development	4.28E-03	1.88E+00	9.99E-01	1.75E-01	7.01E+00
GO:0030324~lung development	4.39E-03	4.56E+00	1.00E+00	1.75E-01	7.18E+00
GO:0050840~extracellular matrix binding	5.14E-03	1.13E+01	8.88E-01	1.97E-01	6.99E+00
GO:0001968~fibronectin binding	5.68E-03	1.09E+01	9.11E-01	1.97E-01	7.70E+00
GO:0001558~regulation of cell growth	5.68E-03	6.96E+00	1.00E+00	2.15E-01	9.19E+00
GO:0005216~ion channel activity	5.87E-03	3.71E+00	9.18E-01	1.88E-01	7.95E+00
GO:0032570~response to progesterone	5.88E-03	1.08E+01	1.00E+00	2.17E-01	9.49E+00
GO:0007229~integrin-mediated signaling pathway	5.89E-03	5.21E+00	1.00E+00	2.13E-01	9.51E+00
GO:0071305~cellular response to vitamin D	6.38E-03	2.42E+01	1.00E+00	2.25E-01	1.03E+01

Term	PValue	Enrichment FC	Bonferroni	Benjamini	FDR
GO:0046339~diacylglycerol metabolic process	6.38E-03	2.42E+01	1.00E+00	2.25E-01	1.03E+01
GO:0071560~cellular response to transforming growth factor beta	6.79E-03	6.62E+00	1.00E+00	2.32E-01	1.09E+01
GO:0071310~cellular response to organic substance	7.69E-03	9.78E+00	1.00E+00	2.54E-01	1.22E+01
GO:0032330~regulation of chondrocyte differentiation	7.74E-03	2.20E+01	1.00E+00	2.51E-01	1.23E+01
GO:0008285~negative regulation of cell proliferation	8.33E-03	2.52E+00	1.00E+00	2.63E-01	1.32E+01
GO:0071300~cellular response to retinoic acid	8.48E-03	6.21E+00	1.00E+00	2.62E-01	1.34E+01
GO:0071456~cellular response to hypoxia	8.99E-03	4.70E+00	1.00E+00	2.71E-01	1.42E+01
GO:0007264~small GTPase mediated signal transduction	9.12E-03	3.08E+00	1.00E+00	2.69E-01	1.44E+01
GO:0019221~cytokine-mediated signaling pathway	9.56E-03	3.87E+00	1.00E+00	2.76E-01	1.50E+01
GO:0043547~positive regulation of GTPase activity	9.56E-03	3.87E+00	1.00E+00	2.76E-01	1.50E+01
GO:0042493~response to drug	9.59E-03	2.62E+00	1.00E+00	2.72E-01	1.50E+01
GO:0031748~D1 dopamine receptor binding	9.61E-03	1.97E+01	9.84E-01	2.71E-01	1.27E+01
GO:0035115~embryonic forelimb morphogenesis	9.79E-03	8.97E+00	1.00E+00	2.73E-01	1.53E+01
GO:0030336~negative regulation of cell migration	1.01E-02	4.57E+00	1.00E+00	2.76E-01	1.58E+01
GO:0008083~growth factor activity	1.02E-02	3.81E+00	9.87E-01	2.69E-01	1.35E+01
GO:0030501~positive regulation of bone mineralization	1.06E-02	8.73E+00	1.00E+00	2.82E-01	1.64E+01
GO:0060411~cardiac septum morphogenesis	1.08E-02	1.86E+01	1.00E+00	2.83E-01	1.68E+01
GO:0003197~endocardial cushion development	1.08E-02	1.86E+01	1.00E+00	2.83E-01	1.68E+01
GO:0043537~negative regulation of blood vessel endothelial cell	1.08E-02	1.86E+01	1.00E+00	2.83E-01	1.68E+01
GO:0032496~response to lipopolysaccharide	1.13E-02	3.28E+00	1.00E+00	2.89E-01	1.74E+01
GO:0008191~metalloendopeptidase inhibitor activity	1.13E-02	1.82E+01	9.92E-01	2.75E-01	1.47E+01
GO:0009887~organ morphogenesis	1.13E-02	4.44E+00	1.00E+00	2.86E-01	1.75E+01
GO:0001568~blood vessel development	1.15E-02	5.68E+00	1.00E+00	2.86E-01	1.78E+01
GO:0045165~cell fate commitment	1.21E-02	5.61E+00	1.00E+00	2.93E-01	1.86E+01
GO:0042472~inner ear morphogenesis	1.21E-02	5.61E+00	1.00E+00	2.93E-01	1.86E+01
GO:0048701~embryonic cranial skeleton morphogenesis	1.22E-02	8.28E+00	1.00E+00	2.92E-01	1.88E+01
GO:0031076~embryonic camera-type eye development	1.25E-02	1.73E+01	1.00E+00	2.94E-01	1.92E+01
GO:0042803~protein homodimerization activity	1.25E-02	1.88E+00	9.95E-01	2.84E-01	1.62E+01
GO:0043434~response to peptide hormone	1.26E-02	5.53E+00	1.00E+00	2.93E-01	1.94E+01
GO:0004857~enzyme inhibitor activity	1.29E-02	8.10E+00	9.96E-01	2.78E-01	1.68E+01
GO:0001649~osteoblast differentiation	1.31E-02	4.29E+00	1.00E+00	2.98E-01	2.00E+01
GO:0030198~extracellular matrix organization	1.35E-02	4.25E+00	1.00E+00	3.03E-01	2.06E+01
GO:0050921~positive regulation of chemotaxis	1.43E-02	1.61E+01	1.00E+00	3.13E-01	2.16E+01
GO:0035909~aorta morphogenesis	1.43E-02	1.61E+01	1.00E+00	3.13E-01	2.16E+01
GO:0043524~negative regulation of neuron apoptotic process	1.45E-02	3.53E+00	1.00E+00	3.13E-01	2.19E+01
GO:0001618~virus receptor activity	1.49E-02	1.58E+01	9.98E-01	2.99E-01	1.91E+01
GO:0060326~cell chemotaxis	1.58E-02	5.17E+00	1.00E+00	3.32E-01	2.36E+01
GO:0043010~camera-type eye development	1.58E-02	5.17E+00	1.00E+00	3.32E-01	2.36E+01
GO:2000649~regulation of sodium ion transmembrane transporter	1.62E-02	1.51E+01	1.00E+00	3.35E-01	2.42E+01
GO:1902043~positive regulation of extrinsic apoptotic signaling	1.62E-02	1.51E+01	1.00E+00	3.35E-01	2.42E+01
GO:0014032~neural crest cell development	1.82E-02	1.42E+01	1.00E+00	3.64E-01	2.68E+01
GO:0000122~negative regulation of transcription from RNA	1.90E-02	1.88E+00	1.00E+00	3.72E-01	2.77E+01
GO:0006813~potassium ion transport	2.06E-02	3.81E+00	1.00E+00	3.93E-01	2.97E+01
GO:0030182~neuron differentiation	2.06E-02	3.81E+00	1.00E+00	3.93E-01	2.97E+01
GO:0060021~palate development	2.10E-02	4.75E+00	1.00E+00	3.94E-01	3.01E+01
GO:0045599~negative regulation of fat cell differentiation	2.13E-02	6.73E+00	1.00E+00	3.94E-01	3.05E+01
GO:0009636~response to toxic substance	2.18E-02	4.69E+00	1.00E+00	3.97E-01	3.11E+01
GO:0044849~estrous cycle	2.26E-02	1.27E+01	1.00E+00	4.04E-01	3.20E+01
GO:1990089~response to nerve growth factor	2.45E-02	8.07E+01	1.00E+00	4.26E-01	3.43E+01
GO:0070172~positive regulation of tooth mineralization	2.45E-02	8.07E+01	1.00E+00	4.26E-01	3.43E+01
GO:0010596~negative regulation of endothelial cell migration	2.49E-02	1.21E+01	1.00E+00	4.27E-01	3.47E+01
GO:0007611~learning or memory	2.50E-02	6.33E+00	1.00E+00	4.24E-01	3.48E+01
GO:0010165~response to X-ray	2.73E-02	1.15E+01	1.00E+00	4.49E-01	3.74E+01
GO:0006954~inflammatory response	2.73E-02	2.35E+00	1.00E+00	4.46E-01	3.74E+01
GO:0071277~cellular response to calcium ion	2.76E-02	6.09E+00	1.00E+00	4.45E-01	3.77E+01
GO:0009968~negative regulation of signal transduction	2.76E-02	6.09E+00	1.00E+00	4.45E-01	3.77E+01
GO:0003151~outflow tract morphogenesis	2.90E-02	5.98E+00	1.00E+00	4.57E-01	3.92E+01
GO:0019900~kinase binding	2.91E-02	4.29E+00	1.00E+00	4.83E-01	3.40E+01
GO:0000976~transcription regulatory region sequence-specific DNA	2.92E-02	5.96E+00	1.00E+00	4.67E-01	3.41E+01
GO:0006807~nitrogen compound metabolic process	2.97E-02	1.10E+01	1.00E+00	4.62E-01	4.00E+01
GO:0048704~embryonic skeletal system morphogenesis	3.04E-02	5.87E+00	1.00E+00	4.65E-01	4.07E+01
GO:0008270~zinc ion binding	3.08E-02	1.62E+00	1.00E+00	4.70E-01	3.57E+01
GO:0030178~negative regulation of Wnt signaling pathway	3.18E-02	5.77E+00	1.00E+00	4.77E-01	4.21E+01
GO:0001701~in utero embryonic development	3.25E-02	2.43E+00	1.00E+00	4.81E-01	4.28E+01
GO:0071837~HMG box domain binding	3.37E-02	1.03E+01	1.00E+00	4.84E-01	3.82E+01
GO:0007519~skeletal muscle tissue development	3.48E-02	5.57E+00	1.00E+00	5.01E-01	4.51E+01
GO:0001894~tissue homeostasis	3.50E-02	1.01E+01	1.00E+00	4.99E-01	4.53E+01

Term	PValue	Enrichment FC	Bonferroni	Benjamini	FDR
GO:0042474~middle ear morphogenesis	3.50E-02	1.01E+01	1.00E+00	4.99E-01	4.53E+01
GO:0051966~regulation of synaptic transmission, glutamatergic	3.50E-02	1.01E+01	1.00E+00	4.99E-01	4.53E+01
GO:0034394~protein localization to cell surface	3.50E-02	1.01E+01	1.00E+00	4.99E-01	4.53E+01
GO:1903598~positive regulation of gap junction assembly	3.65E-02	5.38E+01	1.00E+00	5.11E-01	4.67E+01
GO:0033031~positive regulation of neutrophil apoptotic process	3.65E-02	5.38E+01	1.00E+00	5.11E-01	4.67E+01
GO:0072017~distal tubule development	3.65E-02	5.38E+01	1.00E+00	5.11E-01	4.67E+01
GO:0019065~receptor-mediated endocytosis of virus by host cell	3.65E-02	5.38E+01	1.00E+00	5.11E-01	4.67E+01
GO:0005102~receptor binding	3.66E-02	2.11E+00	1.00E+00	4.98E-01	4.08E+01
GO:0007267~cell-cell signaling	3.87E-02	3.92E+00	1.00E+00	5.27E-01	4.87E+01
GO:0032967~positive regulation of collagen biosynthetic process	4.05E-02	9.31E+00	1.00E+00	5.40E-01	5.03E+01
GO:0035904~aorta development	4.05E-02	9.31E+00	1.00E+00	5.40E-01	5.03E+01
GO:0072659~protein localization to plasma membrane	4.11E-02	5.21E+00	1.00E+00	5.42E-01	5.09E+01
GO:0005507~copper ion binding	4.17E-02	5.18E+00	1.00E+00	5.30E-01	4.51E+01
GO:0043236~laminin binding	4.22E-02	9.11E+00	1.00E+00	5.19E-01	4.55E+01
GO:0001228~transcriptional activator activity, RNA polymerase II	4.27E-02	3.80E+00	1.00E+00	5.10E-01	4.58E+01
GO:0030326~embryonic limb morphogenesis	4.28E-02	5.13E+00	1.00E+00	5.53E-01	5.23E+01
GO:0009612~response to mechanical stimulus	4.28E-02	5.13E+00	1.00E+00	5.53E-01	5.23E+01
GO:0042475~odontogenesis of dentin-containing tooth	4.28E-02	5.13E+00	1.00E+00	5.53E-01	5.23E+01
GO:0042476~odontogenesis	4.34E-02	8.97E+00	1.00E+00	5.54E-01	5.28E+01
GO:0008585~female gonad development	4.34E-02	8.97E+00	1.00E+00	5.54E-01	5.28E+01
GO:0035987~endodermal cell differentiation	4.34E-02	8.97E+00	1.00E+00	5.54E-01	5.28E+01
GO:0030325~adrenal gland development	4.34E-02	8.97E+00	1.00E+00	5.54E-01	5.28E+01
GO:0048146~positive regulation of fibroblast proliferation	4.63E-02	4.97E+00	1.00E+00	5.74E-01	5.51E+01
GO:0055074~calcium ion homeostasis	4.64E-02	8.65E+00	1.00E+00	5.71E-01	5.52E+01
GO:2000352~negative regulation of endothelial cell apoptotic	4.64E-02	8.65E+00	1.00E+00	5.71E-01	5.52E+01
GO:0070373~negative regulation of ERK1 and ERK2 cascade	4.80E-02	4.89E+00	1.00E+00	5.81E-01	5.65E+01
GO:0002634~regulation of germinal center formation	4.84E-02	4.04E+01	1.00E+00	5.80E-01	5.68E+01
GO:0072210~metanephric nephron development	4.84E-02	4.04E+01	1.00E+00	5.80E-01	5.68E+01
GO:0060437~lung growth	4.84E-02	4.04E+01	1.00E+00	5.80E-01	5.68E+01
GO:0060675~ureteric bud morphogenesis	4.84E-02	4.04E+01	1.00E+00	5.80E-01	5.68E+01
GO:0010273~detoxification of copper ion	4.84E-02	4.04E+01	1.00E+00	5.80E-01	5.68E+01
GO:2001258~negative regulation of cation channel activity	4.84E-02	4.04E+01	1.00E+00	5.80E-01	5.68E+01
GO:0007423~sensory organ development	4.84E-02	4.04E+01	1.00E+00	5.80E-01	5.68E+01
GO:0070252~actin-mediated cell contraction	4.84E-02	4.04E+01	1.00E+00	5.80E-01	5.68E+01
GO:0048738~cardiac muscle tissue development	4.94E-02	8.35E+00	1.00E+00	5.84E-01	5.76E+01
GO:0045840~positive regulation of mitotic nuclear division	4.94E-02	8.35E+00	1.00E+00	5.84E-01	5.76E+01
GO:0002040~sprouting angiogenesis	4.94E-02	8.35E+00	1.00E+00	5.84E-01	5.76E+01
GO:0016917~GABA receptor activity	4.95E-02	3.95E+01	1.00E+00	5.50E-01	5.10E+01
GO:0051259~protein oligomerization	4.99E-02	4.82E+00	1.00E+00	5.84E-01	5.79E+01
GO:0043154~negative regulation of cysteine-type endopeptidase	4.99E-02	4.82E+00	1.00E+00	5.84E-01	5.79E+01
GO:0001938~positive regulation of endothelial cell proliferation	4.99E-02	4.82E+00	1.00E+00	5.84E-01	5.79E+01

Supplementary Table S10. Transcripts identified by microarray analysis that were downregulated upon BMS493 treatment in the early ureter. Shown is a list of transcripts that were downregulated after BMS493 treatment of E12.5 ureter explants. Three groups for each condition were compared to untreated controls and the resulting fold changes (FC) in expression are displayed. Intensity thresholds were >100; fold changes were larger than 1.2.

Gene symbol	FC 1	FC 2	FC 3	Avg FC
Tgm5	-15.4	-16.3	-14.4	-15.4
Clca3	-7.3	-16.7	-15.8	-13.3
Tnfsf13b	-10.1	-7.4	-11.1	-9.5
Dhrs3	-6.5	-7.4	-5.7	-6.5
Pnliprp1	-6.3	-5.7	-6.6	-6.2
Il33	-7.6	-5.6	-4.8	-6.0
Sst	-5.1	-4.7	-4.9	-4.9
Elf5	-5.1	-4.7	-4.6	-4.8
Ntsr1	-4.7	-3.4	-4.8	-4.3
Hic1	-4.5	-3.5	-4.3	-4.1
Colq	-4.1	-3.3	-4.9	-4.1
Ecm1	-3.7	-4.1	-4.0	-3.9
Neu3	-4.0	-3.3	-3.5	-3.6
Angptl7	-3.5	-3.5	-3.4	-3.5
Ednrb	-4.0	-2.9	-3.5	-3.5
Shisa3	-3.4	-3.5	-3.2	-3.4
Tgm2	-3.0	-3.2	-3.6	-3.3
Slc38a5	-3.6	-2.5	-3.4	-3.2
Ptplad2	-3.9	-3.0	-2.5	-3.1
Slitrk1	-3.7	-2.5	-2.5	-2.9
Akap12	-2.7	-2.7	-3.2	-2.9
Tmem62	-3.1	-2.9	-2.6	-2.9
Gna14	-3.0	-2.4	-3.1	-2.8
Kcnk2	-2.8	-2.7	-3.0	-2.8
Fut9	-3.1	-2.7	-2.6	-2.8
Rbm46	-2.6	-3.2	-2.4	-2.7
Ogn	-2.4	-2.2	-3.5	-2.7
Spon2	-2.4	-2.8	-2.8	-2.7
Cntn1	-3.0	-2.8	-2.2	-2.7
Adamtsl4	-3.2	-2.3	-2.5	-2.6
Gdf10	-2.7	-2.2	-3.0	-2.6
Sntg1	-2.7	-2.7	-2.3	-2.6
Slitrk5	-3.1	-2.2	-2.5	-2.6
Flrt1	-3.0	-2.1	-2.6	-2.6
AI593442	-2.4	-2.2	-2.9	-2.5
Npy1r	-2.5	-2.4	-2.6	-2.5
AK141540	-2.6	-2.5	-2.4	-2.5
Rarb	-2.4	-2.8	-2.4	-2.5
A_55_P1960936	-2.6	-2.5	-2.4	-2.5
Hoxc4	-2.7	-2.4	-2.3	-2.5
Hs3st6	-3.2	-2.3	-1.9	-2.5
Crhbp	-1.8	-2.5	-3.2	-2.5
BB713741	-2.5	-2.6	-2.3	-2.5
AK046833	-2.8	-2.5	-2.0	-2.5
Cyp7b1	-2.7	-2.2	-2.4	-2.4
Unc13c	-2.6	-2.6	-2.1	-2.4
Sp5	-3.1	-2.2	-2.1	-2.4
Esrrg	-2.6	-2.2	-2.5	-2.4
Adh1	-2.7	-2.3	-2.1	-2.4
Mbd1	-2.5	-2.5	-2.2	-2.4
Crispld2	-2.7	-2.1	-2.3	-2.4
Hapln1	-2.3	-2.0	-2.7	-2.4
Wdr92	-2.6	-2.4	-2.1	-2.3
Gdf5	-2.0	-2.9	-2.1	-2.3
A_55_P1978866	-2.3	-2.5	-2.1	-2.3
Enpp1	-2.4	-2.0	-2.5	-2.3
Fam155a	-2.6	-2.2	-2.1	-2.3
Sprr1a	-2.1	-2.4	-2.3	-2.3
Glrb	-2.3	-2.5	-2.0	-2.3
Vipr1	-2.4	-2.1	-2.3	-2.3
Ccl6	-2.2	-2.1	-2.5	-2.3
Fabp4	-1.9	-2.1	-2.7	-2.2
Kcnj2	-2.5	-2.2	-2.0	-2.2
Nsg2	-2.2	-2.0	-2.4	-2.2
Fap	-2.2	-2.0	-2.4	-2.2
Cd83	-2.3	-2.1	-2.2	-2.2
Anxa9	-2.5	-2.2	-1.9	-2.2
AK035396	-2.4	-2.2	-1.9	-2.2
Cpne8	-2.4	-2.3	-1.8	-2.2
A_55_P1998811	-2.6	-2.3	-1.6	-2.2
Gfra1	-1.9	-1.9	-2.5	-2.1
F830016B08Rik	-2.6	-2.2	-1.6	-2.1
3930401B19Rik	-2.4	-2.1	-1.9	-2.1
Sstr1	-2.4	-1.9	-2.0	-2.1
Gabra3	-2.2	-1.8	-2.3	-2.1
Tspan1	-1.8	-2.4	-2.2	-2.1
A_55_P2051596	-2.5	-2.1	-1.7	-2.1
Pdgfd	-2.2	-2.0	-2.0	-2.1
AK132033	-2.2	-2.1	-1.9	-2.1
Cd38	-2.1	-1.8	-2.3	-2.1
Ephb1	-2.9	-1.8	-1.4	-2.1
Gm4951	-2.3	-2.5	-1.4	-2.0
Synpr	-2.0	-2.0	-2.1	-2.0
Rbp1	-2.1	-1.8	-2.2	-2.0
BC057675	-2.4	-2.1	-1.6	-2.0
TC1616199	-2.4	-2.0	-1.7	-2.0
Cntn6	-1.8	-1.8	-2.4	-2.0
Fabp7	-1.6	-1.3	-3.1	-2.0
A_55_P2107785	-1.9	-1.9	-2.3	-2.0
Ptprd	-2.1	-1.8	-2.0	-2.0
B3galt1	-1.7	-2.5	-1.7	-2.0
Cntn4	-1.7	-1.9	-2.2	-2.0
Iigp1	-2.2	-2.2	-1.5	-2.0
TC1703733	-2.1	-2.4	-1.3	-2.0
Mab21l1	-1.8	-1.8	-2.3	-1.9
5730446D14Rik	-1.9	-2.0	-2.0	-1.9
Moxd1	-2.0	-1.7	-2.1	-1.9
Lurap1l	-1.8	-2.0	-2.0	-1.9
Adamts18	-2.1	-2.1	-1.6	-1.9
Insc	-2.0	-1.4	-2.3	-1.9
Hspa12a	-1.8	-2.1	-1.6	-1.9
Grik4	-1.9	-1.8	-1.9	-1.9
Anxa11	-2.2	-1.8	-1.7	-1.9
Lhfpl2	-2.0	-1.8	-1.8	-1.9
Enpp3	-1.9	-1.8	-1.9	-1.9
Syt6	-1.7	-1.5	-2.3	-1.8
Cmbl	-2.1	-1.5	-1.9	-1.8
AI427809	-2.0	-1.7	-1.8	-1.8

Gene symbol	FC 1	FC 2	FC 3	Avg FC
Stra6	-1.9	-1.5	-2.1	-1.8
D930019F10Rik	-2.3	-1.6	-1.6	-1.8
Kctd8	-2.0	-2.2	-1.2	-1.8
Slc18a3	-1.6	-1.3	-2.6	-1.8
Ptprb	-2.0	-1.5	-1.9	-1.8
6030408B16Rik	-1.8	-1.9	-1.8	-1.8
Igf1	-1.8	-1.7	-2.0	-1.8
Cplx2	-1.8	-1.7	-2.0	-1.8
Otud7b	-1.7	-2.1	-1.6	-1.8
Stmn2	-1.8	-1.5	-2.1	-1.8
Elovl5	-2.0	-1.8	-1.6	-1.8
ENSMUST00000076071	-1.9	-1.4	-2.0	-1.8
Apela	-2.0	-1.6	-1.8	-1.8
Plcb1	-1.9	-1.8	-1.6	-1.8
Lrrc3b	-2.3	-1.7	-1.3	-1.8
Snx22	-1.6	-1.9	-1.8	-1.8
Scnn1a	-1.7	-1.6	-2.0	-1.8
Ly86	-1.9	-1.8	-1.7	-1.8
Ptgs1	-1.6	-1.5	-2.2	-1.8
Oprl1	-1.8	-1.6	-1.9	-1.8
Snap91	-2.0	-1.8	-1.5	-1.8
Car3	-1.7	-1.3	-2.3	-1.7
LOC102640896	-1.6	-1.4	-2.2	-1.7
AI596198	-2.0	-1.8	-1.5	-1.7
Erc2	-2.0	-1.6	-1.6	-1.7
Lgi3	-1.4	-1.6	-2.2	-1.7
Hdhd3	-1.6	-1.9	-1.7	-1.7
Sepp1	-1.7	-1.8	-1.7	-1.7
Edil3	-1.8	-1.8	-1.6	-1.7
Neurl3	-1.6	-1.6	-1.9	-1.7
Ctse	-1.6	-1.6	-1.8	-1.7
Gria4	-1.8	-1.6	-1.7	-1.7
Zfp958	-1.5	-2.3	-1.3	-1.7
Foxa1	-2.0	-1.7	-1.4	-1.7
Hoxc9	-1.9	-1.7	-1.5	-1.7
Adamts8	-1.9	-1.4	-1.8	-1.7
Scd1	-1.7	-1.5	-1.9	-1.7
Scnn1b	-1.8	-1.5	-1.8	-1.7
Hoxc6	-1.8	-1.5	-1.7	-1.7
A_55_P2007495	-1.7	-1.7	-1.6	-1.7
Luzp2	-1.8	-1.7	-1.6	-1.7
AK144717	-1.8	-1.7	-1.6	-1.7
Plekhb1	-1.5	-1.6	-1.9	-1.7
Adra2a	-1.8	-1.3	-1.9	-1.7
Epha5	-2.1	-1.4	-1.5	-1.7
Cyp39a1	-1.6	-1.9	-1.5	-1.7
Aifm2	-1.5	-1.8	-1.7	-1.7
2010300C02Rik	-1.6	-1.7	-1.7	-1.7
Entpd3	-1.4	-1.8	-1.8	-1.7
Upk3a	-1.9	-1.6	-1.5	-1.7
Msln	-1.6	-1.5	-1.8	-1.7
Smim3	-1.8	-1.7	-1.4	-1.6
Wnt9b	-1.5	-1.7	-1.7	-1.6
Negr1	-1.6	-1.5	-1.8	-1.6
A_55_P2114318	-1.7	-1.7	-1.6	-1.6
Ihh	-1.6	-1.9	-1.5	-1.6
A_55_P2090505	-1.8	-1.6	-1.5	-1.6
B430316J06Rik	-1.8	-1.6	-1.6	-1.6
Oxnad1	-1.6	-1.7	-1.6	-1.6
Slitrk4	-1.8	-1.7	-1.4	-1.6
Adcy2	-1.4	-1.6	-1.9	-1.6
Cntn3	-1.4	-1.3	-2.1	-1.6
2810474O19Rik	-1.6	-1.4	-1.7	-1.6
Plac8	-1.7	-1.4	-1.6	-1.6

Gene symbol	FC 1	FC 2	FC 3	Avg FC
Fndc1	-1.8	-1.5	-1.5	-1.6
Calca	-1.7	-1.4	-1.7	-1.6
Usp53	-1.7	-1.8	-1.4	-1.6
Asic4	-1.6	-1.5	-1.6	-1.6
Stxbp5l	-1.8	-1.4	-1.7	-1.6
Adamts15	-1.6	-1.5	-1.7	-1.6
Rab27b	-1.6	-1.7	-1.5	-1.6
Fam78b	-1.6	-1.4	-1.8	-1.6
Trim9	-1.7	-1.4	-1.6	-1.6
Sox7	-1.6	-1.6	-1.6	-1.6
Aqp1	-1.6	-1.6	-1.6	-1.6
Fndc5	-1.6	-1.4	-1.8	-1.6
Olfml1	-1.8	-1.6	-1.4	-1.6
Agtpbp1	-1.8	-1.6	-1.4	-1.6
Stbd1	-1.7	-1.6	-1.5	-1.6
Masp1	-1.8	-1.5	-1.5	-1.6
Mid1ip1	-1.5	-1.6	-1.7	-1.6
Ncoa3	-1.7	-1.3	-1.7	-1.6
Foxd1	-1.4	-1.8	-1.5	-1.6
Ncald	-1.7	-1.4	-1.7	-1.6
F3	-1.8	-1.6	-1.2	-1.6
Mrvi1	-1.5	-1.4	-1.8	-1.6
Cldn11	-1.5	-1.4	-1.7	-1.6
Gata2	-1.3	-1.5	-1.9	-1.6
Gstt3	-1.5	-1.7	-1.5	-1.6
Tmc7	-1.5	-1.5	-1.7	-1.6
Rnf128	-1.8	-1.4	-1.5	-1.6
Ano1	-1.5	-1.4	-1.7	-1.6
Coro2a	-1.4	-1.5	-1.7	-1.6
Foxf1	-1.5	-1.5	-1.7	-1.6
Amigo2	-1.4	-1.5	-1.7	-1.5
6720475J19Rik	-1.4	-2.0	-1.2	-1.5
8430419K02Rik	-1.6	-1.5	-1.6	-1.5
Lmcd1	-1.5	-1.5	-1.6	-1.5
Eva1c	-1.5	-1.3	-1.8	-1.5
Pbx1	-1.7	-1.6	-1.3	-1.5
Casp12	-1.5	-1.4	-1.7	-1.5
Hoxa3	-1.5	-1.5	-1.6	-1.5
Gatm	-1.5	-1.7	-1.4	-1.5
Fam169a	-1.5	-1.4	-1.7	-1.5
Dzank1	-1.5	-1.4	-1.7	-1.5
Aldh1a7	-1.3	-1.8	-1.5	-1.5
Itpka	-1.4	-1.4	-1.8	-1.5
Arg1	-1.6	-1.7	-1.3	-1.5
Ubash3b	-1.9	-1.3	-1.4	-1.5
BB237529	-1.6	-1.7	-1.3	-1.5
Pparg	-1.7	-1.5	-1.3	-1.5
Trim24	-1.5	-1.6	-1.4	-1.5
Clec7a	-1.5	-1.4	-1.7	-1.5
Metrn	-1.3	-2.0	-1.3	-1.5
Hoxc10	-1.4	-1.4	-1.8	-1.5
Pde2a	-1.4	-1.5	-1.6	-1.5
Sema4a	-1.6	-1.4	-1.5	-1.5
Slc16a9	-1.6	-1.3	-1.7	-1.5
Cfl2	-1.6	-1.6	-1.4	-1.5
AK149472	-1.7	-1.6	-1.3	-1.5
Tmprss2	-1.6	-1.6	-1.4	-1.5
Pcsk5	-1.5	-1.4	-1.7	-1.5
Map3k5	-1.5	-1.4	-1.6	-1.5
Mpzl1	-1.7	-1.4	-1.4	-1.5
Fmo5	-1.7	-1.5	-1.4	-1.5
Bace2	-1.4	-1.5	-1.6	-1.5
Gbp2	-1.4	-1.6	-1.5	-1.5
LOC102642336	-1.6	-1.4	-1.5	-1.5

Gene symbol	FC 1	FC 2	FC 3	Avg FC
Tmem27	-1.6	-1.4	-1.5	-1.5
Gucy1a2	-1.5	-1.5	-1.5	-1.5
Efemp1	-1.6	-1.6	-1.3	-1.5
Plat	-1.3	-1.7	-1.5	-1.5
Slc6a15	-1.5	-1.4	-1.6	-1.5
A_55_P1993371	-1.6	-1.6	-1.3	-1.5
Ncoa4	-1.5	-1.6	-1.4	-1.5
Gpr126	-1.6	-1.3	-1.5	-1.5
Gm6557	-1.3	-1.5	-1.6	-1.5
Rpa1	-1.5	-1.6	-1.3	-1.5
Wnt5b	-1.6	-1.4	-1.4	-1.5
Brinp1	-1.4	-1.5	-1.5	-1.5
Gm20939	-1.4	-1.6	-1.5	-1.5
Nrip1	-1.6	-1.5	-1.3	-1.5
Egfr	-1.4	-1.4	-1.6	-1.5
Rnf152	-1.6	-1.5	-1.4	-1.5
Ank2	-1.4	-1.3	-1.8	-1.5
Sprr2g	-1.9	-1.3	-1.3	-1.5
Hdc	-1.6	-1.4	-1.4	-1.5
Magi1	-1.3	-1.5	-1.6	-1.5
Scara3	-1.4	-1.2	-1.8	-1.5
Dhrs7	-1.6	-1.4	-1.4	-1.5
Ncapg	-1.5	-1.5	-1.4	-1.5
Clmn	-1.5	-1.3	-1.6	-1.5
Bcl11a	-1.7	-1.2	-1.4	-1.5
Ephx2	-1.5	-1.6	-1.3	-1.5
Pitx1	-1.7	-1.3	-1.4	-1.5
Gpr116	-1.8	-1.2	-1.4	-1.5
Pou3f1	-1.4	-1.3	-1.6	-1.5
Tshz2	-1.3	-1.4	-1.6	-1.5
Spata33	-1.5	-1.4	-1.5	-1.5
Fbxl2	-1.4	-1.3	-1.6	-1.5
Rad54b	-1.3	-1.7	-1.3	-1.5
Slc40a1	-1.6	-1.5	-1.3	-1.5
Avpr1a	-1.4	-1.4	-1.6	-1.5
Lypd6b	-1.5	-1.4	-1.4	-1.5
Kbtbd11	-1.2	-1.4	-1.7	-1.5
1700017B05Rik	-1.4	-1.4	-1.6	-1.4
A_55_P2091328	-1.5	-1.5	-1.4	-1.4
Snhg18	-1.5	-1.2	-1.7	-1.4
Add3	-1.4	-1.5	-1.5	-1.4
Arl5c	-1.6	-1.4	-1.3	-1.4
Clu	-1.4	-1.4	-1.5	-1.4
Itpr1	-1.4	-1.5	-1.4	-1.4
Mmp2	-1.5	-1.3	-1.5	-1.4
Echdc1	-1.6	-1.3	-1.4	-1.4
Rtn4rl1	-1.4	-1.2	-1.7	-1.4
Fam101a	-1.4	-1.5	-1.4	-1.4
Snrpd3	-1.2	-1.8	-1.2	-1.4
Adam12	-1.4	-1.3	-1.6	-1.4
Larp6	-1.5	-1.3	-1.4	-1.4
Ormdl2	-1.4	-1.2	-1.7	-1.4
Lcorl	-1.4	-1.6	-1.2	-1.4
Adam17	-1.3	-1.4	-1.6	-1.4
S1pr3	-1.3	-1.4	-1.5	-1.4
Fam13a	-1.6	-1.3	-1.4	-1.4
Larp1b	-1.7	-1.4	-1.2	-1.4
Upk1a	-1.4	-1.5	-1.4	-1.4
Prr15	-1.7	-1.3	-1.3	-1.4
St8sia2	-1.4	-1.3	-1.6	-1.4
Egfl6	-1.5	-1.3	-1.4	-1.4
Smco4	-1.3	-1.4	-1.5	-1.4
Cdca7l	-1.3	-1.4	-1.5	-1.4
E530011L22Rik	-1.4	-1.5	-1.3	-1.4

Gene symbol	FC 1	FC 2	FC 3	Avg FC
Bhlhe40	-1.4	-1.3	-1.5	-1.4
Acsl1	-1.6	-1.4	-1.2	-1.4
Dbi	-1.4	-1.4	-1.4	-1.4
Ptp4a3	-1.2	-1.4	-1.7	-1.4
Kcnj10	-1.8	-1.3	-1.2	-1.4
Nudt16	-1.3	-1.4	-1.6	-1.4
Tekt1	-1.4	-1.2	-1.6	-1.4
Clip4	-1.5	-1.4	-1.3	-1.4
Isoc1	-1.4	-1.3	-1.5	-1.4
9030601B04Rik	-1.7	-1.3	-1.2	-1.4
Hoxa5	-1.6	-1.4	-1.2	-1.4
Akr1c14	-1.4	-1.4	-1.4	-1.4
Scarb2	-1.4	-1.4	-1.4	-1.4
Rasl10b	-1.2	-1.3	-1.6	-1.4
Hey2	-1.4	-1.4	-1.5	-1.4
Cntnap2	-1.6	-1.3	-1.3	-1.4
Ccndbp1	-1.4	-1.4	-1.3	-1.4
Lmo2	-1.4	-1.3	-1.5	-1.4
Hoxa2	-1.4	-1.4	-1.4	-1.4
Grik1	-1.4	-1.5	-1.3	-1.4
Sfxn1	-1.6	-1.3	-1.2	-1.4
Pde3b	-1.5	-1.3	-1.3	-1.4
Tbxas1	-1.3	-1.3	-1.6	-1.4
Smyd4	-1.4	-1.5	-1.2	-1.4
Hmgn3	-1.2	-1.3	-1.6	-1.4
Stard5	-1.3	-1.3	-1.6	-1.4
Rab33a	-1.3	-1.2	-1.6	-1.4
Rec8	-1.4	-1.4	-1.3	-1.4
G630016G05Rik	-1.3	-1.3	-1.5	-1.4
Dna2	-1.4	-1.4	-1.4	-1.4
Tle3	-1.5	-1.2	-1.4	-1.4
Zfp445	-1.2	-1.7	-1.2	-1.4
Mylip	-1.2	-1.5	-1.4	-1.4
Veph1	-1.2	-1.6	-1.3	-1.4
Cacnb2	-1.3	-1.3	-1.5	-1.4
Hey1	-1.5	-1.3	-1.3	-1.4
Bcl3	-1.3	-1.3	-1.6	-1.4
Abca1	-1.4	-1.5	-1.3	-1.4
Clhc1	-1.4	-1.5	-1.3	-1.4
Tmeff2	-1.2	-1.3	-1.6	-1.4
Wfs1	-1.5	-1.3	-1.4	-1.4
Lbh	-1.6	-1.3	-1.3	-1.4
Car13	-1.3	-1.3	-1.5	-1.4
Rrm2	-1.4	-1.4	-1.4	-1.4
Syndig1	-1.4	-1.4	-1.3	-1.4
Fgf7	-1.5	-1.3	-1.3	-1.4
Cdc25b	-1.3	-1.3	-1.6	-1.4
Bmp7	-1.6	-1.2	-1.3	-1.4
Klhl13	-1.5	-1.3	-1.2	-1.4
Rpgrip1	-1.7	-1.2	-1.2	-1.4
Rnf144a	-1.4	-1.3	-1.4	-1.4
Plxnc1	-1.3	-1.2	-1.5	-1.4
Ropn1l	-1.4	-1.4	-1.3	-1.4
Neil3	-1.4	-1.3	-1.4	-1.4
Cenpk	-1.3	-1.2	-1.5	-1.4
LOC102635234	-1.4	-1.3	-1.4	-1.4
Upk1b	-1.3	-1.5	-1.3	-1.4
Tmem164	-1.4	-1.5	-1.2	-1.4
Cldn8	-1.4	-1.4	-1.3	-1.4
Rad51b	-1.4	-1.3	-1.4	-1.4
Tspan13	-1.5	-1.4	-1.2	-1.4
Klf3	-1.4	-1.3	-1.3	-1.4
Stac	-1.3	-1.5	-1.3	-1.4
Syt16	-1.5	-1.4	-1.2	-1.4

Gene symbol	FC 1	FC 2	FC 3	Avg FC
Enpp2	-1.3	-1.3	-1.4	-1.4
Vcan	-1.4	-1.3	-1.4	-1.4
Kank4	-1.3	-1.3	-1.4	-1.4
Rab11fip1	-1.5	-1.4	-1.2	-1.4
Phyhd1	-1.5	-1.2	-1.4	-1.4
Slc24a5	-1.4	-1.3	-1.3	-1.3
Rab8b	-1.4	-1.2	-1.4	-1.3
Zeb1	-1.4	-1.2	-1.5	-1.3
Gal3st4	-1.3	-1.2	-1.5	-1.3
Vegfc	-1.3	-1.3	-1.5	-1.3
Tm7sf2	-1.4	-1.3	-1.3	-1.3
Zfp930	-1.3	-1.5	-1.2	-1.3
Celf2	-1.5	-1.2	-1.3	-1.3
Flrt3	-1.6	-1.3	-1.2	-1.3
Olfm1	-1.5	-1.3	-1.2	-1.3
Rad51	-1.4	-1.4	-1.3	-1.3
Xkr5	-1.4	-1.2	-1.4	-1.3
6030451C04Rik	-1.4	-1.3	-1.3	-1.3
Neto2	-1.4	-1.2	-1.4	-1.3
Jarid2	-1.4	-1.2	-1.4	-1.3
Fam210b	-1.3	-1.4	-1.3	-1.3
Gsr	-1.4	-1.4	-1.3	-1.3
Hoxb2	-1.4	-1.3	-1.3	-1.3
Glb1l2	-1.5	-1.3	-1.3	-1.3
Stx17	-1.2	-1.2	-1.6	-1.3
Mettl20	-1.4	-1.3	-1.3	-1.3
Zfp937	-1.5	-1.2	-1.2	-1.3
Hrsp12	-1.5	-1.3	-1.2	-1.3
LOC545966	-1.4	-1.4	-1.2	-1.3
Pcdh7	-1.4	-1.3	-1.3	-1.3
Pdpn	-1.4	-1.3	-1.3	-1.3
AI429812	-1.5	-1.3	-1.2	-1.3
Acss2	-1.5	-1.2	-1.2	-1.3
Pdgfc	-1.3	-1.3	-1.3	-1.3
2810417H13Rik	-1.4	-1.3	-1.3	-1.3
Ebf3	-1.3	-1.3	-1.4	-1.3
Lcor	-1.3	-1.4	-1.3	-1.3
Mif	-1.3	-1.4	-1.3	-1.3
Rab37	-1.3	-1.2	-1.4	-1.3
Gse1	-1.3	-1.2	-1.4	-1.3
Camta1	-1.4	-1.3	-1.3	-1.3
Spdya	-1.4	-1.3	-1.3	-1.3
Slc43a3	-1.4	-1.3	-1.3	-1.3
A_55_P2024391	-1.3	-1.4	-1.3	-1.3
Hexb	-1.3	-1.4	-1.2	-1.3
Nuf2	-1.4	-1.2	-1.3	-1.3
Exo1	-1.4	-1.3	-1.3	-1.3
Unc13b	-1.2	-1.4	-1.3	-1.3
Rnf125	-1.4	-1.4	-1.2	-1.3
Slc19a2	-1.3	-1.3	-1.3	-1.3
Epb4.1l4b	-1.4	-1.2	-1.4	-1.3
Akap5	-1.5	-1.2	-1.2	-1.3
Lypd6	-1.3	-1.4	-1.2	-1.3
ENSMUST00000063463	-1.4	-1.3	-1.2	-1.3
Tifa	-1.3	-1.3	-1.3	-1.3
Zranb3	-1.5	-1.2	-1.2	-1.3
Fblim1	-1.4	-1.3	-1.3	-1.3
Dcaf6	-1.3	-1.3	-1.4	-1.3
Fancd2	-1.2	-1.4	-1.4	-1.3
Ccnj	-1.3	-1.4	-1.3	-1.3
Phf6	-1.3	-1.4	-1.2	-1.3
Gyg	-1.3	-1.2	-1.4	-1.3
Plscr1	-1.3	-1.3	-1.3	-1.3
Arhgap12	-1.4	-1.3	-1.2	-1.3
Smagp	-1.3	-1.2	-1.4	-1.3

Gene symbol	FC 1	FC 2	FC 3	Avg FC
0610040J01Rik	-1.3	-1.3	-1.3	-1.3
AK140216	-1.5	-1.2	-1.2	-1.3
Krt23	-1.4	-1.2	-1.3	-1.3
Pbk	-1.3	-1.3	-1.3	-1.3
Fxyd6	-1.3	-1.2	-1.4	-1.3
Rbpms	-1.3	-1.2	-1.3	-1.3
Tmem35	-1.4	-1.3	-1.2	-1.3
Mms19	-1.3	-1.3	-1.4	-1.3
Hsd17b7	-1.3	-1.3	-1.2	-1.3
Acat2	-1.3	-1.3	-1.3	-1.3
Vopp1	-1.2	-1.3	-1.4	-1.3
Tesc	-1.3	-1.2	-1.3	-1.3
Pask	-1.2	-1.4	-1.3	-1.3
Olfml3	-1.2	-1.2	-1.4	-1.3
Ifit2	-1.3	-1.3	-1.3	-1.3
Fanca	-1.2	-1.3	-1.3	-1.3
Fam115c	-1.2	-1.3	-1.3	-1.3
Ncapg2	-1.4	-1.2	-1.3	-1.3
Mis18bp1	-1.2	-1.2	-1.4	-1.3
Brca1	-1.2	-1.3	-1.3	-1.3
Fry	-1.4	-1.3	-1.2	-1.3
Tnnt1	-1.2	-1.2	-1.4	-1.3
Fbxo30	-1.3	-1.3	-1.3	-1.3
Pcdhb4	-1.3	-1.3	-1.3	-1.3
Adam22	-1.3	-1.3	-1.2	-1.3
Dctn5	-1.2	-1.2	-1.4	-1.3
Fads2	-1.3	-1.2	-1.4	-1.3
Zfp69	-1.2	-1.2	-1.4	-1.3
Gpa33	-1.4	-1.2	-1.2	-1.3
Qk	-1.3	-1.3	-1.2	-1.3
Cisd3	-1.3	-1.3	-1.2	-1.3
Ahr	-1.2	-1.2	-1.4	-1.3
6430573F11Rik	-1.2	-1.2	-1.3	-1.3
H2-T9	-1.3	-1.2	-1.3	-1.3
Trp53inp1	-1.3	-1.3	-1.2	-1.3
Gmnn	-1.3	-1.2	-1.2	-1.3
Uaca	-1.3	-1.3	-1.2	-1.3
Pold3	-1.3	-1.3	-1.2	-1.3
Xndc1	-1.2	-1.2	-1.4	-1.3
Eml5	-1.3	-1.2	-1.2	-1.3
Mtfr2	-1.2	-1.3	-1.3	-1.3
Vps13d	-1.2	-1.2	-1.4	-1.3
Zmiz1	-1.3	-1.2	-1.3	-1.3
Slc39a8	-1.3	-1.2	-1.3	-1.3
Ptpru	-1.3	-1.3	-1.2	-1.3
Mex3b	-1.3	-1.2	-1.2	-1.3
Rai2	-1.3	-1.3	-1.2	-1.3
H2afy3	-1.3	-1.3	-1.2	-1.3
Nr2c1	-1.2	-1.2	-1.3	-1.3
Nhlrc1	-1.2	-1.2	-1.3	-1.3
Arhgap18	-1.3	-1.3	-1.2	-1.3
Gpam	-1.2	-1.3	-1.3	-1.3
Chd1l	-1.2	-1.2	-1.3	-1.3
2210008F06Rik	-1.2	-1.3	-1.3	-1.2
Idh1	-1.2	-1.3	-1.3	-1.2
Mreg	-1.2	-1.2	-1.3	-1.2
Melk	-1.2	-1.3	-1.2	-1.2
Nxnl2	-1.2	-1.3	-1.2	-1.2
Mns1	-1.3	-1.2	-1.2	-1.2
Mgst1	-1.2	-1.2	-1.3	-1.2
Gsg2	-1.2	-1.2	-1.2	-1.2
Ercc6l	-1.2	-1.2	-1.2	-1.2
Golm1	-1.2	-1.2	-1.3	-1.2
Cdc45	-1.2	-1.2	-1.2	-1.2

Supplementary Table S11. Transcripts identified by microarray analysis that were upregulated upon RA treatment in the early ureter. Shown is a list of transcripts that were upregulated after RA treatment of E12.5 ureter explants. Three groups for each condition were compared to untreated controls and the resulting fold changes (FC) in expression are displayed. Intensity thresholds were >100; fold changes were larger than 1.2.

Gene symbol	FC 1	FC 2	FC 3	Avg FC
Cyp26a1	17.3	17.3	14.8	16.5
Cyp26b1	14.9	13.3	14.1	14.1
Lrat	11.4	9.8	10.0	10.4
Fgf15	7.8	7.7	6.6	7.4
Hoxb13	5.5	7.4	6.4	6.4
Csn3	7.1	7.5	4.5	6.4
Ecm1	5.8	6.0	5.6	5.8
Bhlhe22	4.0	5.4	7.9	5.7
Nkx3-1	4.7	6.7	5.5	5.7
Htr2b	5.8	4.6	6.3	5.5
Sst	5.1	5.2	5.1	5.1
Tnfsf13b	4.5	5.0	5.3	4.9
Sh3bgr	4.1	5.9	3.9	4.6
Vipr1	3.7	4.2	5.5	4.5
Tspan11	3.8	4.8	3.7	4.1
Adamtsl4	3.9	4.0	4.2	4.1
Fgf5	3.0	3.8	5.1	3.9
5730585A16Rik	3.1	3.7	4.8	3.9
Cox8b	2.9	4.5	3.9	3.8
Gbp2	4.7	3.8	2.3	3.6
Hoxc4	3.0	3.2	4.6	3.6
NAP113361-1	3.4	3.3	4.2	3.6
Gcnt3	3.9	3.8	3.0	3.6
6530411M01Rik	3.4	3.4	3.6	3.5
TC1616199	3.6	3.6	3.1	3.4
Onecut2	3.1	3.3	3.8	3.4
2700046A07Rik	3.9	3.4	2.9	3.4
Nlrp6	2.7	3.7	3.8	3.4
Il33	3.7	2.8	3.6	3.4
Ntsr1	2.5	3.1	4.4	3.3
AK141540	3.3	3.4	3.1	3.2
Hic1	3.0	3.2	3.5	3.2
Myo18b	3.9	3.3	2.4	3.2
Olfm1	3.1	3.4	2.9	3.2
B4galnt2	2.3	2.7	4.4	3.1
Hoxa1	2.9	3.2	2.9	3.0
Dhrs3	2.7	2.8	3.6	3.0
3930401B19Rik	3.0	2.9	3.0	3.0
AK132033	3.0	2.9	3.0	2.9
AK035396	3.2	2.8	2.8	2.9
Gna14	2.8	3.1	2.8	2.9
Pnliprp2	2.7	2.8	3.2	2.9
Wt1	2.3	2.3	4.0	2.9
Hoxa4	2.9	3.1	2.6	2.9
A_55_P2023176	3.5	2.8	2.3	2.9
Brinp3	3.5	2.6	2.4	2.8
0610042G04Rik	3.1	2.6	2.8	2.8
Lyve1	3.3	2.6	2.4	2.7
Hspa12a	2.4	2.0	3.8	2.7
Gm13178	2.5	2.8	2.8	2.7
Kcnk2	2.1	2.5	3.5	2.7
Stra6	2.3	2.6	3.2	2.7
Zbtb7c	2.4	2.4	3.2	2.7
Angptl7	2.8	2.7	2.4	2.7

Gene symbol	FC 1	FC 2	FC 3	Avg FC
BB086117	2.1	3.0	2.9	2.7
Lrrc7	3.0	2.4	2.6	2.6
Cyp7b1	2.5	2.7	2.8	2.6
Gm5105	2.3	3.0	2.5	2.6
Asic2	2.7	3.0	2.1	2.6
Ackr4	2.6	2.3	2.9	2.6
Colq	2.2	2.8	2.7	2.6
Shisa9	3.2	2.6	2.0	2.6
Pnliprp1	1.9	2.7	3.1	2.6
Whrn	2.6	2.8	2.4	2.6
Wdr92	2.4	2.6	2.5	2.5
Slitrk1	2.8	2.3	2.5	2.5
Fbxo32	2.1	2.8	2.6	2.5
BC057675	2.8	1.8	2.5	2.4
Grk5	2.2	2.5	2.5	2.4
Flrt1	2.4	1.8	3.0	2.4
Adh1	2.0	2.6	2.6	2.4
Hoxc8	2.4	2.0	2.8	2.4
St8sia3	2.6	2.6	2.0	2.4
Hoxc9	2.5	2.2	2.4	2.4
LOC102637716	2.6	2.3	2.2	2.4
ENSMUST0000004810	2.4	2.7	2.0	2.4
Shisa3	2.3	2.1	2.6	2.3
Itgb3	2.7	2.5	1.7	2.3
Slc14a2	2.2	2.5	2.2	2.3
A_55_P2090505	2.3	2.2	2.5	2.3
Slc18a3	1.9	2.5	2.6	2.3
Tspan1	1.9	2.2	2.8	2.3
Adamts18	3.5	1.4	1.9	2.3
Erc2	2.2	2.4	2.3	2.3
Chat	2.4	1.6	2.9	2.3
Kank4	1.8	2.4	2.4	2.2
Ihh	2.0	2.1	2.5	2.2
Itpka	2.1	2.1	2.3	2.2
Paqr7	1.9	2.8	1.9	2.2
B230334C09Rik	2.1	2.1	2.3	2.2
Scube1	2.5	1.4	2.6	2.2
Slc16a12	2.0	2.6	1.9	2.2
Pnlip	2.2	2.1	2.2	2.2
Il1rapl1	2.0	1.8	2.7	2.2
Cntn1	1.9	1.6	3.0	2.2
Igf1	2.1	2.4	2.0	2.2
Nxnl2	2.2	2.1	2.2	2.1
Efemp1	2.5	2.0	1.9	2.1
Ppp1r3c	2.2	2.3	1.9	2.1
AI593442	1.5	2.3	2.4	2.1
Iqsec3	1.9	2.3	2.1	2.1
Rpp25	2.2	2.2	1.9	2.1
Usp53	1.7	2.0	2.5	2.1
Islr2	2.1	2.3	1.8	2.1
Tmem62	2.0	2.0	2.2	2.1
Enpp3	2.0	2.0	2.2	2.1
AI596198	2.1	1.8	2.3	2.1
Hoxa5	2.4	1.7	2.1	2.1

Gene symbol	FC 1	FC 2	FC 3	Avg FC
A_55_P1960936	1.9	2.0	2.2	2.1
Hs3st6	2.1	2.3	1.8	2.1
Neto1	2.5	2.0	1.7	2.0
Tmem255a	1.4	2.1	2.7	2.0
Ephx2	1.9	1.8	2.4	2.0
Asic4	1.8	1.8	2.4	2.0
Art4	2.2	2.1	1.8	2.0
Clec7a	1.5	1.8	2.6	2.0
AK149472	2.2	1.7	2.1	2.0
Tgm2	2.0	2.1	1.9	2.0
Sstr1	2.3	2.1	1.6	2.0
Gpr37	1.8	2.0	2.2	2.0
Gfra1	1.7	2.1	2.1	2.0
Esrrg	1.8	1.8	2.2	2.0
Igfbp6	2.3	1.8	1.7	2.0
Pdzd2	1.9	2.0	2.0	2.0
Akap12	1.7	2.2	2.0	2.0
Lhfpl2	1.7	2.0	2.1	2.0
Spon2	2.0	2.0	1.8	1.9
Ccdc85a	1.7	1.7	2.4	1.9
Fut9	1.6	1.5	2.7	1.9
Mbd1	1.8	1.8	2.2	1.9
Cd38	1.6	2.3	1.9	1.9
BB713741	1.7	1.9	2.2	1.9
Cd83	1.8	1.9	2.0	1.9
Gdf7	1.8	1.9	2.1	1.9
AK046833	1.7	1.5	2.5	1.9
Hoxa3	1.7	1.6	2.4	1.9
Grik4	1.9	1.8	2.0	1.9
Scrt1	1.7	2.0	2.1	1.9
Dtx4	1.6	2.1	1.9	1.9
Hoxa2	2.1	1.6	1.9	1.9
Slc38a5	1.8	1.7	2.1	1.9
Rarb	1.8	1.8	2.0	1.9
Ubash3b	2.3	1.6	1.8	1.9
Tgm5	1.6	1.9	2.1	1.9
Muc1	1.7	2.3	1.6	1.9
Gm2990	1.9	1.9	1.8	1.9
Ptger3	1.8	1.7	2.1	1.9
AK140216	1.6	1.8	2.1	1.9
Gbp7	2.4	1.7	1.5	1.9
Sord	2.1	1.6	1.9	1.9
Slc24a3	1.5	1.9	2.2	1.8
Foxc2	1.8	1.8	1.9	1.8
Lrfn2	1.6	2.0	1.9	1.8
Gdf5	1.8	1.7	2.1	1.8
Nckap1l	1.4	2.1	2.0	1.8
Bcl2l13	1.9	1.7	1.8	1.8
A_55_P1978866	1.7	1.6	2.1	1.8
Ifitm5	1.7	1.9	1.8	1.8
Eva1c	1.7	2.2	1.5	1.8
Zfp958	1.6	1.5	2.4	1.8
Pitx1	1.5	1.9	2.0	1.8
Clmn	1.7	1.8	2.0	1.8
Smug1	1.5	1.9	2.0	1.8
Pde11a	1.8	1.6	2.0	1.8
Sipa1l2	1.5	1.9	2.0	1.8
Casz1	1.5	2.0	1.8	1.8
Fabp4	1.9	1.8	1.7	1.8
Fmn2	1.3	1.9	2.2	1.8
Mical3	1.5	1.9	2.0	1.8
Pbx1	1.5	1.7	2.2	1.8
Syt6	1.4	2.1	1.9	1.8
Crispld2	1.7	1.9	1.7	1.8

Gene symbol	FC 1	FC 2	FC 3	Avg FC
Noxa1	1.7	1.9	1.8	1.8
Masp1	1.4	1.8	2.1	1.8
Fmo5	1.6	1.7	2.1	1.8
Dep1	1.5	1.4	2.4	1.8
Abi1	1.6	1.8	2.0	1.8
Fndc5	1.5	1.8	1.9	1.8
Muc20	2.0	1.9	1.5	1.8
Hpca	1.6	1.6	2.1	1.8
Synpr	2.1	1.5	1.7	1.8
Dcx	1.6	2.0	1.7	1.8
Hoxb2	1.7	1.8	1.8	1.8
AI427809	1.7	1.7	1.9	1.8
Sp5	1.5	1.6	2.1	1.8
Fstl5	1.5	1.8	2.0	1.8
Fam155a	1.7	1.7	1.8	1.8
Abcg5	1.6	1.7	2.0	1.8
Fst	2.3	1.6	1.3	1.7
Ecel1	1.6	1.8	1.8	1.7
Anxa11	1.5	1.8	1.9	1.7
A_55_P1998811	1.6	1.7	2.0	1.7
Ncoa4	1.5	1.6	2.0	1.7
Gm527	1.8	1.6	1.7	1.7
Rbm46	1.8	1.6	1.7	1.7
Gas7	1.5	1.7	1.9	1.7
Iigp1	1.8	1.5	1.9	1.7
Tec	1.6	1.8	1.7	1.7
TC1703733	1.9	1.2	1.9	1.7
H2-M3	1.8	1.9	1.3	1.7
Ptprr	1.9	1.6	1.5	1.7
Rbp1	1.6	1.6	1.9	1.7
LOC269472	1.7	1.5	1.8	1.7
Gm3893	1.7	1.4	2.0	1.7
3632451O06Rik	1.7	1.7	1.7	1.7
Slc2a13	1.5	1.7	1.9	1.7
Hey2	1.6	2.0	1.4	1.7
Kcnj12	1.7	2.0	1.3	1.7
Hoxb8	1.4	1.7	2.0	1.7
Gata2	1.3	1.8	1.9	1.7
Tmem132e	1.7	1.8	1.5	1.7
Cxcl13	1.9	1.2	1.9	1.7
LOC101056232	1.7	1.4	1.9	1.7
Tcf15	1.8	1.8	1.4	1.7
St8sia2	1.4	1.8	1.8	1.7
Tbx4	1.4	1.7	1.9	1.7
Foxl1	1.6	1.8	1.7	1.7
Mreg	1.5	1.9	1.6	1.7
5730446D14Rik	1.9	1.5	1.6	1.7
Gse1	1.6	1.5	1.9	1.7
Grin2c	1.6	1.6	1.8	1.7
Coro2a	1.5	1.8	1.6	1.7
Fry	1.5	1.8	1.7	1.7
Hoxc10	1.6	1.6	1.7	1.7
Hoxc6	1.3	1.7	1.9	1.6
Adra2a	1.3	1.7	1.9	1.6
Egfl6	1.4	1.7	1.9	1.6
Abhd15	1.4	1.5	2.0	1.6
Stard5	1.5	1.9	1.6	1.6
Cplx2	1.5	1.5	2.0	1.6
Ube2ql1	1.8	1.3	1.9	1.6
Lgr5	1.5	1.5	2.0	1.6
Hdhd3	1.8	1.6	1.5	1.6
Camk2n1	1.9	1.5	1.5	1.6
C230071I02Rik	1.5	1.2	2.2	1.6
Tex15	1.4	1.9	1.5	1.6

Gene symbol	FC 1	FC 2	FC 3	Avg FC
Ednrb	1.4	1.7	1.8	1.6
Hivep1	1.5	1.8	1.6	1.6
Arhgap33	1.6	1.7	1.6	1.6
Mab21l1	1.8	1.6	1.5	1.6
A_55_P2159595	1.4	1.6	1.8	1.6
Clhc1	1.8	1.6	1.4	1.6
Hoxa6	2.1	1.2	1.5	1.6
Fam183b	1.8	1.5	1.5	1.6
Nrip1	1.3	1.6	1.9	1.6
Sprr1a	1.7	1.7	1.4	1.6
Ccbl2	1.5	1.6	1.7	1.6
Mpzl1	1.6	1.6	1.6	1.6
Galnt6	1.4	2.2	1.2	1.6
Gm4951	2.0	1.3	1.6	1.6
Akap2	1.5	1.5	1.8	1.6
Adamts8	1.4	1.6	1.9	1.6
2010300C02Rik	1.6	1.6	1.6	1.6
Gabrb3	1.2	2.0	1.6	1.6
Abca1	1.4	1.3	2.0	1.6
Mgll	1.4	1.7	1.6	1.6
Gm12992	1.5	1.7	1.6	1.6
Tgfb3	1.6	1.8	1.5	1.6
Add3	1.3	1.5	1.9	1.6
Mcl1	1.3	1.7	1.7	1.6
Kbtbd11	1.5	1.8	1.5	1.6
Neto2	1.6	1.7	1.5	1.6
4632428N05Rik	1.6	1.8	1.3	1.6
Pou3f1	1.3	1.8	1.7	1.6
Dcaf6	1.5	1.6	1.7	1.6
Pth1r	1.5	1.7	1.5	1.6
Hoxb5	1.5	1.6	1.6	1.6
Ptprb	1.5	1.3	1.8	1.6
A_55_P2051596	1.4	1.5	1.8	1.6
Foxd1	1.6	1.3	1.9	1.6
Rnf165	1.6	1.5	1.6	1.6
D4Ertd681e	1.6	1.2	1.9	1.6
6030408B16Rik	1.7	1.6	1.4	1.6
Hoxb4	1.6	1.3	1.7	1.6
Syndig1	1.3	1.6	1.8	1.6
Nbeal2	1.5	1.8	1.3	1.6
Vps13d	1.4	1.5	1.8	1.6
Cdc25b	1.4	1.7	1.5	1.6
Gata6	1.5	1.5	1.7	1.6
Slc7a7	1.6	1.6	1.4	1.5
E2f2	1.7	1.4	1.6	1.5
Kcnk3	1.5	1.6	1.6	1.5
Ifitm1	1.6	1.7	1.3	1.5
Syk	1.3	1.6	1.7	1.5
Frmd5	1.5	1.5	1.6	1.5
Elf5	1.4	1.4	1.8	1.5
Ttll5	1.7	1.4	1.5	1.5
1700017B05Rik	1.3	1.7	1.6	1.5
Gm6557	1.4	1.7	1.6	1.5
TC1711418	1.5	1.3	1.7	1.5
Mast1	1.8	1.5	1.3	1.5
Aldh1l1	1.5	1.5	1.5	1.5
Tshz1	1.4	1.4	1.8	1.5
Tlx2	1.4	1.6	1.5	1.5
S1pr3	1.3	1.6	1.6	1.5
Rtn1	1.6	1.5	1.6	1.5
Gdf10	1.4	1.5	1.6	1.5
Ephb1	1.5	1.5	1.6	1.5
Scd1	1.4	1.9	1.3	1.5
Acss1	1.3	1.4	1.8	1.5

Gene symbol	FC 1	FC 2	FC 3	Avg FC
2210411A11Rik	1.4	1.3	1.8	1.5
Nsg2	1.5	1.4	1.6	1.5
Sfrp1	1.3	1.9	1.4	1.5
Enpp1	1.5	1.7	1.3	1.5
Gpa33	1.5	1.7	1.3	1.5
Sh3kbp1	1.4	1.6	1.6	1.5
A_55_P2107785	1.5	1.7	1.4	1.5
Cmbl	1.6	1.5	1.5	1.5
Clip4	1.8	1.3	1.4	1.5
AI661453	1.3	1.9	1.4	1.5
Hk2	1.6	1.5	1.5	1.5
Car14	1.6	1.7	1.2	1.5
Elavl3	1.7	1.4	1.5	1.5
Hoxb3	1.4	1.2	1.9	1.5
B130019D13Rik	1.4	1.3	1.8	1.5
Ackr3	1.6	1.4	1.5	1.5
Ampd3	1.3	1.6	1.5	1.5
Pdp2	1.3	1.8	1.4	1.5
Pde2a	1.3	1.8	1.4	1.5
Trim9	1.4	1.5	1.6	1.5
Agfg2	1.7	1.2	1.6	1.5
Mex3b	1.7	1.2	1.5	1.5
Fgf18	1.7	1.2	1.6	1.5
Gm4907	1.8	1.3	1.3	1.5
Ano1	1.6	1.5	1.4	1.5
Calca	1.6	1.5	1.3	1.5
Tes	1.6	1.5	1.4	1.5
Traf3ip2	1.4	1.6	1.4	1.5
Gm11744	1.6	1.6	1.3	1.5
A_55_P2007495	1.3	1.5	1.6	1.5
H2afz	1.5	1.4	1.5	1.5
Tmem108	1.3	1.4	1.7	1.5
Sez6l2	1.2	1.6	1.6	1.5
Irf8	1.5	1.6	1.3	1.5
Ppp2r3d	1.3	1.6	1.5	1.5
Smim3	1.8	1.4	1.3	1.5
Cfl2	1.3	1.5	1.7	1.5
Rtn4rl1	1.2	1.6	1.6	1.5
AK032893	1.6	1.4	1.4	1.5
Raph1	1.6	1.3	1.6	1.5
Fblim1	1.4	1.3	1.7	1.5
Mpped2	1.6	1.3	1.5	1.5
Cilp	1.3	1.6	1.4	1.5
Acsl1	1.5	1.4	1.5	1.5
Tshz2	1.3	1.4	1.7	1.5
Cpz	1.4	1.6	1.4	1.5
Hapln1	1.4	1.5	1.5	1.5
Shisa7	1.6	1.5	1.3	1.5
Anxa9	1.5	1.5	1.4	1.5
Foxp4	1.4	1.5	1.5	1.5
A_55_P2114318	1.3	1.5	1.6	1.5
Slitrk5	1.2	1.3	1.9	1.5
Fam78b	1.3	1.4	1.8	1.5
Bace2	1.6	1.3	1.4	1.5
A_55_P2087281	1.3	1.5	1.5	1.5
Xkr5	1.3	1.8	1.4	1.5
Emilin2	1.4	1.5	1.4	1.5
Ptp4a3	1.4	1.7	1.3	1.5
Sfxn1	1.6	1.2	1.5	1.4
Skap2	1.5	1.4	1.4	1.4
Ptpru	1.4	1.4	1.6	1.4
Gm4841	1.5	1.4	1.4	1.4
Gpr116	1.3	1.5	1.6	1.4
Npy1r	1.5	1.4	1.5	1.4

Gene symbol	FC 1	FC 2	FC 3	Avg FC
Rgl1	1.4	1.3	1.6	1.4
Spata33	1.6	1.3	1.4	1.4
Ccdc177	1.4	1.3	1.6	1.4
Ina	1.6	1.3	1.4	1.4
Nxn	1.2	1.4	1.6	1.4
Por	1.3	1.4	1.6	1.4
Image:619641	1.2	1.4	1.7	1.4
Fbxo2	1.4	1.5	1.4	1.4
Pnpla3	1.8	1.3	1.2	1.4
Rbpms	1.2	1.6	1.5	1.4
Oxnad1	1.5	1.4	1.4	1.4
Zfp937	1.4	1.3	1.6	1.4
Palm2	1.4	1.5	1.3	1.4
Hivep3	1.6	1.4	1.2	1.4
Ntn1	1.5	1.3	1.5	1.4
Kdm5b	1.5	1.3	1.5	1.4
Atp8a1	1.3	1.5	1.6	1.4
Dll1	1.4	1.5	1.4	1.4
Cenpm	1.7	1.4	1.2	1.4
Tnni3	1.6	1.3	1.3	1.4
Rasl10b	1.3	1.5	1.5	1.4
Tm7sf2	1.5	1.5	1.3	1.4
Pparg	1.2	1.5	1.6	1.4
Bmp7	1.3	1.5	1.5	1.4
Plekhb1	1.5	1.5	1.3	1.4
Scd3	1.6	1.4	1.3	1.4
Lrfn5	1.3	1.4	1.6	1.4
Qk	1.4	1.2	1.7	1.4
Chst8	1.4	1.5	1.3	1.4
Ush1c	1.4	1.3	1.5	1.4
Zscan29	1.3	1.2	1.7	1.4
Megf9	1.5	1.3	1.5	1.4
Enpep	1.2	1.3	1.7	1.4
Flrt3	1.4	1.3	1.5	1.4
Sdk1	1.2	1.5	1.5	1.4
Gpx3	1.4	1.5	1.3	1.4
Nkx1-1	1.5	1.3	1.4	1.4
Cyp2d11	1.5	1.4	1.3	1.4
Rec8	1.2	1.6	1.4	1.4
Uaca	1.3	1.4	1.5	1.4
Gmnn	1.4	1.4	1.4	1.4
Flrt2	1.4	1.4	1.5	1.4
Dapp1	1.3	1.6	1.4	1.4
Fads2	1.3	1.5	1.5	1.4
Fam101a	1.6	1.2	1.4	1.4
Git1	1.3	1.3	1.6	1.4
Spock2	1.2	1.5	1.5	1.4
Hist1h3i	1.5	1.4	1.3	1.4
Neurl3	1.3	1.6	1.4	1.4
Agtpbp1	1.4	1.4	1.4	1.4
Fap	1.3	1.4	1.4	1.4
Larp6	1.4	1.5	1.3	1.4
Ccnf	1.4	1.3	1.5	1.4
Hist1h3g	1.4	1.4	1.4	1.4
Kcnj10	1.2	1.4	1.5	1.4
Gstt3	1.3	1.5	1.4	1.4
Plat	1.3	1.4	1.5	1.4
Nefl	1.6	1.3	1.2	1.4
Lrrc3b	1.6	1.3	1.2	1.4
Hapln3	1.4	1.4	1.3	1.4
AK139043	1.3	1.3	1.6	1.4
Tars2	1.4	1.2	1.5	1.4
NAP112768-1	1.5	1.5	1.2	1.4
Aifm2	1.3	1.4	1.4	1.4

Gene symbol	FC 1	FC 2	FC 3	Avg FC
E030030I06Rik	1.4	1.5	1.2	1.4
Tgfb2	1.3	1.3	1.5	1.4
Zfp395	1.3	1.6	1.3	1.4
Ada	1.4	1.4	1.3	1.4
Adam12	1.3	1.6	1.2	1.4
Wdr12	1.4	1.4	1.3	1.4
A_55_P2175050	1.4	1.3	1.4	1.4
Ptch2	1.5	1.4	1.3	1.4
Stmn2	1.6	1.3	1.2	1.4
Foxf1	1.2	1.3	1.6	1.4
Insc	1.3	1.5	1.4	1.4
Bicd1	1.3	1.3	1.5	1.4
Mgat5b	1.3	1.5	1.3	1.4
Maged2	1.3	1.4	1.4	1.4
3110007F17Rik	1.4	1.5	1.2	1.4
Dzank1	1.4	1.3	1.5	1.4
4930422N03Rik	1.3	1.3	1.5	1.4
Gm10125	1.5	1.3	1.2	1.4
Mycl	1.2	1.3	1.5	1.4
Vipr2	1.4	1.5	1.3	1.4
Defb25	1.5	1.4	1.3	1.4
Ankrd13b	1.2	1.4	1.5	1.4
Arg1	1.4	1.5	1.2	1.4
Ccnjl	1.3	1.4	1.3	1.4
Hectd2	1.2	1.6	1.3	1.4
Isoc1	1.3	1.4	1.3	1.4
Fam131a	1.2	1.3	1.6	1.4
Hist1h1e	1.4	1.3	1.3	1.3
Ccndbp1	1.4	1.3	1.4	1.3
Grrp1	1.4	1.4	1.2	1.3
Hoxb6	1.3	1.3	1.4	1.3
Myl6b	1.2	1.4	1.4	1.3
Ncoa3	1.2	1.5	1.3	1.3
Srrm4	1.2	1.4	1.4	1.3
Doc2b	1.3	1.5	1.2	1.3
Dpf3	1.4	1.4	1.2	1.3
Faah	1.3	1.5	1.3	1.3
Tpx2	1.2	1.5	1.3	1.3
Scnn1b	1.3	1.4	1.4	1.3
Pde1b	1.3	1.3	1.4	1.3
Pcdh11x	1.3	1.5	1.2	1.3
Btg1	1.3	1.3	1.4	1.3
St3gal1	1.2	1.4	1.4	1.3
Zfp423	1.2	1.3	1.5	1.3
Trim24	1.2	1.2	1.5	1.3
Gm9099	1.3	1.3	1.3	1.3
Mrvi1	1.2	1.5	1.3	1.3
Ulk4	1.2	1.5	1.2	1.3
Kcnab3	1.2	1.3	1.4	1.3
NAP112100-1	1.4	1.3	1.3	1.3
Dnaaf3	1.3	1.4	1.3	1.3
Spry1	1.4	1.3	1.3	1.3
Ropn1l	1.3	1.3	1.4	1.3
Oprl1	1.3	1.5	1.3	1.3
Acaca	1.3	1.4	1.4	1.3
Resp18	1.4	1.3	1.2	1.3
Gcnt1	1.3	1.4	1.3	1.3
Nell1	1.2	1.3	1.4	1.3
Unc5a	1.3	1.3	1.3	1.3
Cntnap2	1.3	1.3	1.4	1.3
Fanca	1.3	1.2	1.4	1.3
Papss2	1.4	1.4	1.2	1.3
Pmepa1	1.4	1.3	1.2	1.3
Cdk15	1.4	1.2	1.3	1.3

Gene symbol	FC 1	FC 2	FC 3	Avg FC
Shcbp1	1.4	1.3	1.3	1.3
Gm13298	1.3	1.3	1.4	1.3
Ckap2l	1.3	1.3	1.3	1.3
Nfam1	1.2	1.3	1.4	1.3
E2f8	1.3	1.4	1.3	1.3
Chka	1.2	1.5	1.2	1.3
Ccl19	1.3	1.3	1.3	1.3
Tspan15	1.3	1.3	1.4	1.3
A_55_P2152407	1.3	1.3	1.3	1.3
Fam83d	1.3	1.3	1.3	1.3
Creg1	1.3	1.3	1.3	1.3
BB237529	1.3	1.3	1.4	1.3
Efcab11	1.3	1.3	1.3	1.3
Usp1	1.2	1.2	1.5	1.3
Tpd52	1.4	1.3	1.3	1.3
A_55_P2024391	1.4	1.3	1.2	1.3
Tmem27	1.3	1.4	1.2	1.3
Map3k5	1.3	1.3	1.3	1.3
2900008C10Rik	1.5	1.2	1.2	1.3
Chd7	1.4	1.2	1.3	1.3
Pitpnm2	1.2	1.4	1.3	1.3
Pitpnc1	1.2	1.2	1.4	1.3
Pdgfd	1.3	1.3	1.3	1.3
Ddx25	1.4	1.2	1.2	1.3
Clec2l	1.3	1.3	1.3	1.3
Ybx2	1.2	1.2	1.4	1.3
Cck	1.3	1.3	1.3	1.3
2810468N07Rik	1.4	1.2	1.3	1.3
Rbm22	1.3	1.2	1.4	1.3
Stac	1.2	1.2	1.4	1.3
Eml1	1.2	1.2	1.4	1.3
Celf2	1.3	1.3	1.2	1.3
Nrcam	1.2	1.4	1.3	1.3
Pbk	1.3	1.3	1.2	1.3
Mansc4	1.4	1.2	1.2	1.3
Slc4a8	1.2	1.4	1.2	1.3
Rufy3	1.2	1.4	1.2	1.3
Ttk	1.3	1.3	1.2	1.3
Eefsec	1.3	1.3	1.3	1.3
Celf3	1.3	1.2	1.3	1.3
1700019D03Rik	1.2	1.3	1.2	1.3
Trak1	1.2	1.2	1.3	1.3
Kif26b	1.2	1.3	1.3	1.3
Mybl2	1.2	1.3	1.2	1.3
Pdlim7	1.2	1.3	1.2	1.3
1700048M11Rik	1.3	1.3	1.2	1.2
Egfr	1.2	1.3	1.2	1.2
Dna2	1.2	1.3	1.2	1.2
Chd1l	1.3	1.2	1.2	1.2
Kif2c	1.2	1.3	1.2	1.2
3110043O21Rik	1.2	1.3	1.2	1.2
B930049P21Rik	1.2	1.2	1.2	1.2
Neil1	1.2	1.2	1.3	1.2
Epb4.1	1.2	1.2	1.2	1.2

Supplementary Table S12. Transcripts identified by microarray analysis that were positively regulated by RA in the early ureter. Shown is a list of transcripts that were positively regulated by RA, i.e. downregulated after BMS493 treatment and upregulated after RA treatment of E12.5 ureter explants. Three groups were for each condition were compared to untreated controls and the resulting fold changes (FC) in expression are displayed. Intensity thresholds were >100; fold changes were larger than 1.2.

Rank	Gene symbol	BMS493 FC 1	BMS493 FC 2	BMS493 FC 3	BMS493 Avg FC	RA FC 1	RA FC 2	RA FC 3	RA Avg FC
1	Tgm5	-15.4	-16.3	-14.4	-15.4	1.6	1.9	2.1	1.9
2	Tnfsf13b	-10.1	-7.4	-11.1	-9.5	4.5	5.0	5.3	4.9
3	Dhrs3	-6.5	-7.4	-5.7	-6.5	2.7	2.8	3.6	3.0
4	Pnliprp1	-6.3	-5.7	-6.6	-6.2	1.9	2.7	3.1	2.6
5	Il33	-7.6	-5.6	-4.8	-6.0	3.7	2.8	3.6	3.4
6	Sst	-5.1	-4.7	-4.9	-4.9	5.1	5.2	5.1	5.1
7	Elf5	-5.1	-4.7	-4.6	-4.8	1.4	1.4	1.8	1.5
8	Ntsr1	-4.7	-3.4	-4.8	-4.3	2.5	3.1	4.4	3.3
9	Hic1	-4.5	-3.5	-4.3	-4.1	3.0	3.2	3.5	3.2
10	Colq	-4.1	-3.3	-4.9	-4.1	2.2	2.8	2.7	2.6
11	Ecm1	-3.7	-4.1	-4.0	-3.9	5.8	6.0	5.6	5.8
12	Angptl7	-3.5	-3.5	-3.4	-3.5	2.8	2.7	2.4	2.7
13	Ednrb	-4.0	-2.9	-3.5	-3.5	1.4	1.7	1.8	1.6
14	Shisa3	-3.4	-3.5	-3.2	-3.4	2.3	2.1	2.6	2.3
15	Tgm2	-3.0	-3.2	-3.6	-3.3	2.0	2.1	1.9	2.0
16	Slc38a5	-3.6	-2.5	-3.4	-3.2	1.8	1.7	2.1	1.9
17	Slitrk1	-3.7	-2.5	-2.5	-2.9	2.8	2.3	2.5	2.5
18	Akap12	-2.7	-2.7	-3.2	-2.9	1.7	2.2	2.0	2.0
19	Tmem62	-3.1	-2.9	-2.6	-2.9	2.0	2.0	2.2	2.1
20	Gna14	-3.0	-2.4	-3.1	-2.8	2.8	3.1	2.8	2.9
21	Kcnk2	-2.8	-2.7	-3.0	-2.8	2.1	2.5	3.5	2.7
22	Fut9	-3.1	-2.7	-2.6	-2.8	1.6	1.5	2.7	1.9
23	Rbm46	-2.6	-3.2	-2.4	-2.7	1.8	1.6	1.7	1.7
24	Spon2	-2.4	-2.8	-2.8	-2.7	2.0	2.0	1.8	1.9
25	Cntn1	-3.0	-2.8	-2.2	-2.7	1.9	1.6	3.0	2.2
26	Adamtsl4	-3.2	-2.3	-2.5	-2.6	3.9	4.0	4.2	4.1
27	Gdf10	-2.7	-2.2	-3.0	-2.6	1.4	1.5	1.6	1.5
28	Slitrk5	-3.1	-2.2	-2.5	-2.6	1.2	1.3	1.9	1.5
29	Flrt1	-3.0	-2.1	-2.6	-2.6	2.4	1.8	3.0	2.4
30	AI593442	-2.4	-2.2	-2.9	-2.5	1.5	2.3	2.4	2.1
31	Npy1r	-2.5	-2.4	-2.6	-2.5	1.5	1.4	1.5	1.4
32	AK141540	-2.6	-2.5	-2.4	-2.5	3.3	3.4	3.1	3.2
33	Rarb	-2.4	-2.8	-2.4	-2.5	1.8	1.8	2.0	1.9
34	A_55_P1960936	-2.6	-2.5	-2.4	-2.5	1.9	2.0	2.2	2.1
35	Hoxc4	-2.7	-2.4	-2.3	-2.5	3.0	3.2	4.6	3.6
36	Hs3st6	-3.2	-2.3	-1.9	-2.5	2.1	2.3	1.8	2.1
37	BB713741	-2.5	-2.6	-2.3	-2.5	1.7	1.9	2.2	1.9
38	AK046833	-2.8	-2.5	-2.0	-2.5	1.7	1.5	2.5	1.9
39	Cyp7b1	-2.7	-2.2	-2.4	-2.4	2.5	2.7	2.8	2.6
40	Sp5	-3.1	-2.2	-2.1	-2.4	1.5	1.6	2.1	1.8
41	Esrrg	-2.6	-2.2	-2.5	-2.4	1.8	1.8	2.2	2.0
42	Adh1	-2.7	-2.3	-2.1	-2.4	2.0	2.6	2.6	2.4
43	Mbd1	-2.5	-2.5	-2.2	-2.4	1.8	1.8	2.2	1.9
44	Crispld2	-2.7	-2.1	-2.3	-2.4	1.7	1.9	1.7	1.8
45	Hapln1	-2.3	-2.0	-2.7	-2.4	1.4	1.5	1.5	1.5
46	Wdr92	-2.6	-2.4	-2.1	-2.3	2.4	2.6	2.5	2.5
47	Gdf5	-2.0	-2.9	-2.1	-2.3	1.8	1.7	2.1	1.8
48	A_55_P1978866	-2.3	-2.5	-2.1	-2.3	1.7	1.6	2.1	1.8
49	Enpp1	-2.4	-2.0	-2.5	-2.3	1.5	1.7	1.3	1.5
50	Fam155a	-2.6	-2.2	-2.1	-2.3	1.7	1.7	1.8	1.8
51	Sprr1a	-2.1	-2.4	-2.3	-2.3	1.7	1.7	1.4	1.6
52	Vipr1	-2.4	-2.1	-2.3	-2.3	3.7	4.2	5.5	4.5
53	Fabp4	-1.9	-2.1	-2.7	-2.2	1.9	1.8	1.7	1.8
54	Nsg2	-2.2	-2.0	-2.4	-2.2	1.5	1.4	1.6	1.5
55	Fap	-2.2	-2.0	-2.4	-2.2	1.3	1.4	1.4	1.4
56	Cd83	-2.3	-2.1	-2.2	-2.2	1.8	1.9	2.0	1.9
57	Anxa9	-2.5	-2.2	-1.9	-2.2	1.5	1.5	1.4	1.5

Rank	Gene symbol	BMS493 FC 1	BMS493 FC 2	BMS493 FC 3	BMS493 Avg FC	RA FC 1	RA FC 2	RA FC 3	RA Avg FC
58	AK035396	-2.4	-2.2	-1.9	-2.2	3.2	2.8	2.8	2.9
59	A_55_P1998811	-2.6	-2.3	-1.6	-2.2	1.6	1.7	2.0	1.7
60	Gfra1	-1.9	-1.9	-2.5	-2.1	1.7	2.1	2.1	2.0
61	3930401B19Rik	-2.4	-2.1	-1.9	-2.1	3.0	2.9	3.0	3.0
62	Sstr1	-2.4	-1.9	-2.0	-2.1	2.3	2.1	1.6	2.0
63	Tspan1	-1.8	-2.4	-2.2	-2.1	1.9	2.2	2.8	2.3
64	A_55_P2051596	-2.5	-2.1	-1.7	-2.1	1.4	1.5	1.8	1.6
65	Pdgfd	-2.2	-2.0	-2.0	-2.1	1.3	1.3	1.3	1.3
66	AK132033	-2.2	-2.1	-1.9	-2.1	3.0	2.9	3.0	2.9
67	Cd38	-2.1	-1.8	-2.3	-2.1	1.6	2.3	1.9	1.9
68	Ephb1	-2.9	-1.8	-1.4	-2.1	1.5	1.5	1.6	1.5
69	Gm4951	-2.3	-2.5	-1.4	-2.0	2.0	1.3	1.6	1.6
70	Synpr	-2.0	-2.0	-2.1	-2.0	2.1	1.5	1.7	1.8
71	Rbp1	-2.1	-1.8	-2.2	-2.0	1.6	1.6	1.9	1.7
72	BC057675	-2.4	-2.1	-1.6	-2.0	2.8	1.8	2.5	2.4
73	TC1616199	-2.4	-2.0	-1.7	-2.0	3.6	3.6	3.1	3.4
74	A_55_P2107785	-1.9	-1.9	-2.3	-2.0	1.5	1.7	1.4	1.5
75	Iigp1	-2.2	-2.2	-1.5	-2.0	1.8	1.5	1.9	1.7
76	TC1703733	-2.1	-2.4	-1.3	-2.0	1.9	1.2	1.9	1.7
77	Mab21l1	-1.8	-1.8	-2.3	-1.9	1.8	1.6	1.5	1.6
78	5730446D14Rik	-1.9	-2.0	-2.0	-1.9	1.9	1.5	1.6	1.7
79	Adamts18	-2.1	-2.1	-1.6	-1.9	3.5	1.4	1.9	2.3
80	Insc	-2.0	-1.4	-2.3	-1.9	1.3	1.5	1.4	1.4
81	Hspa12a	-1.8	-2.1	-1.6	-1.9	2.4	2.0	3.8	2.7
82	Grik4	-1.9	-1.8	-1.9	-1.9	1.9	1.8	2.0	1.9
83	Anxa11	-2.2	-1.8	-1.7	-1.9	1.5	1.8	1.9	1.7
84	Lhfpl2	-2.0	-1.8	-1.8	-1.9	1.7	2.0	2.1	2.0
85	Enpp3	-1.9	-1.8	-1.9	-1.9	2.0	2.0	2.2	2.1
86	Syt6	-1.7	-1.5	-2.3	-1.8	1.4	2.1	1.9	1.8
87	Cmbl	-2.1	-1.5	-1.9	-1.8	1.6	1.5	1.5	1.5
88	AI427809	-2.0	-1.7	-1.8	-1.8	1.7	1.7	1.9	1.8
89	Stra6	-1.9	-1.5	-2.1	-1.8	2.3	2.6	3.2	2.7
90	Slc18a3	-1.6	-1.3	-2.6	-1.8	1.9	2.5	2.6	2.3
91	Ptprb	-2.0	-1.5	-1.9	-1.8	1.5	1.3	1.8	1.6
92	6030408B16Rik	-1.8	-1.9	-1.8	-1.8	1.7	1.6	1.4	1.6
93	Igf1	-1.8	-1.7	-2.0	-1.8	2.1	2.4	2.0	2.2
94	Cplx2	-1.8	-1.7	-2.0	-1.8	1.5	1.5	2.0	1.6
95	Stmn2	-1.8	-1.5	-2.1	-1.8	1.6	1.3	1.2	1.4
96	Lrrc3b	-2.3	-1.7	-1.3	-1.8	1.6	1.3	1.2	1.4
97	Oprl1	-1.8	-1.6	-1.9	-1.8	1.3	1.5	1.3	1.3
98	AI596198	-2.0	-1.8	-1.5	-1.7	2.1	1.8	2.3	2.1
99	Erc2	-2.0	-1.6	-1.6	-1.7	2.2	2.4	2.3	2.3
100	Hdhd3	-1.6	-1.9	-1.7	-1.7	1.8	1.6	1.5	1.6
101	Neurl3	-1.6	-1.6	-1.9	-1.7	1.3	1.6	1.4	1.4
102	Zfp958	-1.5	-2.3	-1.3	-1.7	1.6	1.5	2.4	1.8
103	Hoxc9	-1.9	-1.7	-1.5	-1.7	2.5	2.2	2.4	2.4
104	Adamts8	-1.9	-1.4	-1.8	-1.7	1.4	1.6	1.9	1.6
105	Scd1	-1.7	-1.5	-1.9	-1.7	1.4	1.9	1.3	1.5
106	Scnn1b	-1.8	-1.5	-1.8	-1.7	1.3	1.4	1.4	1.3
107	Hoxc6	-1.8	-1.5	-1.7	-1.7	1.3	1.7	1.9	1.6
108	A_55_P2007495	-1.7	-1.7	-1.6	-1.7	1.3	1.5	1.6	1.5
109	Plekhb1	-1.5	-1.6	-1.9	-1.7	1.5	1.5	1.3	1.4
110	Adra2a	-1.8	-1.3	-1.9	-1.7	1.3	1.7	1.9	1.6
111	Aifm2	-1.5	-1.8	-1.7	-1.7	1.3	1.4	1.4	1.4
112	2010300C02Rik	-1.6	-1.7	-1.7	-1.7	1.6	1.6	1.6	1.6
113	Smim3	-1.8	-1.7	-1.4	-1.6	1.8	1.4	1.3	1.5
114	A_55_P2114318	-1.7	-1.7	-1.6	-1.6	1.3	1.5	1.6	1.5
115	Ihh	-1.6	-1.9	-1.5	-1.6	2.0	2.1	2.5	2.2
116	A_55_P2090505	-1.8	-1.6	-1.5	-1.6	2.3	2.2	2.5	2.3
117	Oxnad1	-1.6	-1.7	-1.6	-1.6	1.5	1.4	1.4	1.4
118	Calca	-1.7	-1.4	-1.7	-1.6	1.6	1.5	1.3	1.5
119	Usp53	-1.7	-1.8	-1.4	-1.6	1.7	2.0	2.5	2.1
120	Asic4	-1.6	-1.5	-1.6	-1.6	1.8	1.8	2.4	2.0
121	Fam78b	-1.6	-1.4	-1.8	-1.6	1.3	1.4	1.8	1.5
122	Trim9	-1.7	-1.4	-1.6	-1.6	1.4	1.5	1.6	1.5
123	Fndc5	-1.6	-1.4	-1.8	-1.6	1.5	1.8	1.9	1.8
124	Agtpbp1	-1.8	-1.6	-1.4	-1.6	1.4	1.4	1.4	1.4

Rank	Gene symbol	BMS493 FC 1	BMS493 FC 2	BMS493 FC 3	BMS493 Avg FC	RA FC 1	RA FC 2	RA FC 3	RA Avg FC
125	Masp1	-1.8	-1.5	-1.5	-1.6	1.4	1.8	2.1	1.8
126	Ncoa3	-1.7	-1.3	-1.7	-1.6	1.2	1.5	1.3	1.3
127	Foxd1	-1.4	-1.8	-1.5	-1.6	1.6	1.3	1.9	1.6
128	Mrvi1	-1.5	-1.4	-1.8	-1.6	1.2	1.5	1.3	1.3
129	Gata2	-1.3	-1.5	-1.9	-1.6	1.3	1.8	1.9	1.7
130	Gstt3	-1.5	-1.7	-1.5	-1.6	1.3	1.5	1.4	1.4
131	Ano1	-1.5	-1.4	-1.7	-1.6	1.6	1.5	1.4	1.5
132	Coro2a	-1.4	-1.5	-1.7	-1.6	1.5	1.8	1.6	1.7
133	Foxf1	-1.5	-1.5	-1.7	-1.6	1.2	1.3	1.6	1.4
134	Eva1c	-1.5	-1.3	-1.8	-1.5	1.7	2.2	1.5	1.8
135	Pbx1	-1.7	-1.6	-1.3	-1.5	1.5	1.7	2.2	1.8
136	Hoxa3	-1.5	-1.5	-1.6	-1.5	1.7	1.6	2.4	1.9
137	Dzank1	-1.5	-1.4	-1.7	-1.5	1.4	1.3	1.5	1.4
138	Itpka	-1.4	-1.4	-1.8	-1.5	2.1	2.1	2.3	2.2
139	Arg1	-1.6	-1.7	-1.3	-1.5	1.4	1.5	1.2	1.4
140	Ubash3b	-1.9	-1.3	-1.4	-1.5	2.3	1.6	1.8	1.9
141	BB237529	-1.6	-1.7	-1.3	-1.5	1.3	1.3	1.4	1.3
142	Pparg	-1.7	-1.5	-1.3	-1.5	1.2	1.5	1.6	1.4
143	Trim24	-1.5	-1.6	-1.4	-1.5	1.2	1.2	1.5	1.3
144	Clec7a	-1.5	-1.4	-1.7	-1.5	1.5	1.8	2.6	2.0
145	Hoxc10	-1.4	-1.4	-1.8	-1.5	1.6	1.6	1.7	1.7
146	Pde2a	-1.4	-1.5	-1.6	-1.5	1.3	1.8	1.4	1.5
147	Cfl2	-1.6	-1.6	-1.4	-1.5	1.3	1.5	1.7	1.5
148	AK149472	-1.7	-1.6	-1.3	-1.5	2.2	1.7	2.1	2.0
149	Map3k5	-1.5	-1.4	-1.6	-1.5	1.3	1.3	1.3	1.3
150	Mpzl1	-1.7	-1.4	-1.4	-1.5	1.6	1.6	1.6	1.6
151	Fmo5	-1.7	-1.5	-1.4	-1.5	1.6	1.7	2.1	1.8
152	Bace2	-1.4	-1.5	-1.6	-1.5	1.6	1.3	1.4	1.5
153	Gbp2	-1.4	-1.6	-1.5	-1.5	4.7	3.8	2.3	3.6
154	Tmem27	-1.6	-1.4	-1.5	-1.5	1.3	1.4	1.2	1.3
155	Efemp1	-1.6	-1.6	-1.3	-1.5	2.5	2.0	1.9	2.1
156	Plat	-1.3	-1.7	-1.5	-1.5	1.3	1.4	1.5	1.4
157	Ncoa4	-1.5	-1.6	-1.4	-1.5	1.5	1.6	2.0	1.7
158	Gm6557	-1.3	-1.5	-1.6	-1.5	1.4	1.7	1.6	1.5
159	Nrip1	-1.6	-1.5	-1.3	-1.5	1.3	1.6	1.9	1.6
160	Egfr	-1.4	-1.4	-1.6	-1.5	1.2	1.3	1.2	1.2
161	Clmn	-1.5	-1.3	-1.6	-1.5	1.7	1.8	2.0	1.8
162	Ephx2	-1.5	-1.6	-1.3	-1.5	1.9	1.8	2.4	2.0
163	Pitx1	-1.7	-1.3	-1.4	-1.5	1.5	1.9	2.0	1.8
164	Gpr116	-1.8	-1.2	-1.4	-1.5	1.3	1.5	1.6	1.4
165	Pou3f1	-1.4	-1.3	-1.6	-1.5	1.3	1.8	1.7	1.6
166	Tshz2	-1.3	-1.4	-1.6	-1.5	1.3	1.4	1.7	1.5
167	Spata33	-1.5	-1.4	-1.5	-1.5	1.6	1.3	1.4	1.4
168	Kbtbd11	-1.2	-1.4	-1.7	-1.5	1.5	1.8	1.5	1.6
169	1700017B05Rik	-1.4	-1.4	-1.6	-1.4	1.3	1.7	1.6	1.5
170	Add3	-1.4	-1.5	-1.5	-1.4	1.3	1.5	1.9	1.6
171	Rtn4rl1	-1.4	-1.2	-1.7	-1.4	1.2	1.6	1.6	1.5
172	Fam101a	-1.4	-1.5	-1.4	-1.4	1.6	1.2	1.4	1.4
173	Adam12	-1.4	-1.3	-1.6	-1.4	1.3	1.6	1.2	1.4
174	Larp6	-1.5	-1.3	-1.4	-1.4	1.4	1.5	1.3	1.4
175	S1pr3	-1.3	-1.4	-1.5	-1.4	1.3	1.6	1.6	1.5
176	St8sia2	-1.4	-1.3	-1.6	-1.4	1.4	1.8	1.8	1.7
177	Egfl6	-1.5	-1.3	-1.4	-1.4	1.4	1.7	1.9	1.6
178	Acsl1	-1.6	-1.4	-1.2	-1.4	1.5	1.4	1.5	1.5
179	Ptp4a3	-1.2	-1.4	-1.7	-1.4	1.4	1.7	1.3	1.5
180	Kcnj10	-1.8	-1.3	-1.2	-1.4	1.2	1.4	1.5	1.4
181	Clip4	-1.5	-1.4	-1.3	-1.4	1.8	1.3	1.4	1.5
182	Isoc1	-1.4	-1.3	-1.5	-1.4	1.3	1.4	1.3	1.4
183	Hoxa5	-1.6	-1.4	-1.2	-1.4	2.4	1.7	2.1	2.1
184	Rasl10b	-1.2	-1.3	-1.6	-1.4	1.3	1.5	1.5	1.4
185	Hey2	-1.4	-1.4	-1.5	-1.4	1.6	2.0	1.4	1.7
186	Cntnap2	-1.6	-1.3	-1.3	-1.4	1.3	1.3	1.4	1.3
187	Ccndbp1	-1.4	-1.4	-1.3	-1.4	1.4	1.3	1.4	1.3
188	Hoxa2	-1.4	-1.4	-1.4	-1.4	2.1	1.6	1.9	1.9
189	Sfxn1	-1.6	-1.3	-1.2	-1.4	1.6	1.2	1.5	1.4
190	Stard5	-1.3	-1.3	-1.6	-1.4	1.5	1.9	1.6	1.6
191	Rec8	-1.4	-1.4	-1.3	-1.4	1.2	1.6	1.4	1.4

Rank	Gene symbol	BMS493 FC 1	BMS493 FC 2	BMS493 FC 3	BMS493 Avg FC	RA FC 1	RA FC 2	RA FC 3	RA Avg FC
192	Dna2	-1.4	-1.4	-1.4	-1.4	1.2	1.3	1.2	1.2
193	Abca1	-1.4	-1.5	-1.3	-1.4	1.4	1.3	2.0	1.6
194	Clhc1	-1.4	-1.5	-1.3	-1.4	1.8	1.6	1.4	1.6
195	Syndig1	-1.4	-1.4	-1.3	-1.4	1.3	1.6	1.8	1.6
196	Cdc25b	-1.3	-1.3	-1.6	-1.4	1.4	1.7	1.5	1.6
197	Bmp7	-1.6	-1.2	-1.3	-1.4	1.3	1.5	1.5	1.4
198	Ropn1l	-1.4	-1.4	-1.3	-1.4	1.3	1.3	1.4	1.3
199	Stac	-1.3	-1.5	-1.3	-1.4	1.2	1.2	1.4	1.3
200	Kank4	-1.3	-1.3	-1.4	-1.4	1.8	2.4	2.4	2.2
201	Tm7sf2	-1.4	-1.3	-1.3	-1.3	1.5	1.5	1.3	1.4
202	Celf2	-1.5	-1.2	-1.3	-1.3	1.3	1.3	1.2	1.3
203	Flrt3	-1.6	-1.3	-1.2	-1.3	1.4	1.3	1.5	1.4
204	Olfm1	-1.5	-1.3	-1.2	-1.3	3.1	3.4	2.9	3.2
205	Xkr5	-1.4	-1.2	-1.4	-1.3	1.3	1.8	1.4	1.5
206	Neto2	-1.4	-1.2	-1.4	-1.3	1.6	1.7	1.5	1.6
207	Hoxb2	-1.4	-1.3	-1.3	-1.3	1.7	1.8	1.8	1.8
208	Zfp937	-1.5	-1.2	-1.2	-1.3	1.4	1.3	1.6	1.4
209	Gse1	-1.3	-1.2	-1.4	-1.3	1.6	1.5	1.9	1.7
210	A_55_P2024391	-1.3	-1.4	-1.3	-1.3	1.4	1.3	1.2	1.3
211	Fblim1	-1.4	-1.3	-1.3	-1.3	1.4	1.3	1.7	1.5
212	Dcaf6	-1.3	-1.3	-1.4	-1.3	1.5	1.6	1.7	1.6
213	AK140216	-1.5	-1.2	-1.2	-1.3	1.6	1.8	2.1	1.9
214	Pbk	-1.3	-1.3	-1.3	-1.3	1.3	1.3	1.2	1.3
215	Rbpms	-1.3	-1.2	-1.3	-1.3	1.2	1.6	1.5	1.4
216	Fanca	-1.2	-1.3	-1.3	-1.3	1.3	1.2	1.4	1.3
217	Fry	-1.4	-1.3	-1.2	-1.3	1.5	1.8	1.7	1.7
218	Fads2	-1.3	-1.2	-1.4	-1.3	1.3	1.5	1.5	1.4
219	Gpa33	-1.4	-1.2	-1.2	-1.3	1.5	1.7	1.3	1.5
220	Qk	-1.3	-1.3	-1.2	-1.3	1.4	1.2	1.7	1.4
221	Gmnn	-1.3	-1.2	-1.2	-1.3	1.4	1.4	1.4	1.4
222	Uaca	-1.3	-1.3	-1.2	-1.3	1.3	1.4	1.5	1.4
223	Vps13d	-1.2	-1.2	-1.4	-1.3	1.4	1.5	1.8	1.6
224	Ptpru	-1.3	-1.3	-1.2	-1.3	1.4	1.4	1.6	1.4
225	Mex3b	-1.3	-1.2	-1.2	-1.3	1.7	1.2	1.5	1.5
226	Chd1l	-1.2	-1.2	-1.3	-1.3	1.3	1.2	1.2	1.2
227	Mreg	-1.2	-1.2	-1.3	-1.2	1.5	1.9	1.6	1.7
228	Nxnl2	-1.2	-1.3	-1.2	-1.2	2.2	2.1	2.2	2.1

Supplementary Table S13. Functional enrichment analysis for transcripts that were positively regulated by RA in the early ureter. Functional enrichment analysis for 228 genes as performed with DAVID websoftware (https://david.ncifcrf.gov) using default settings. Shown are enriched terms for the annotation categories/databases GO:biological process and GO:molecular function with a p-value p<0.05.

Term	PValue	Enrichment FC	Bonferroni	Benjamini	FDR
GO:0043565~sequence-specific DNA binding	1.06E-06	3.53E+00	3.65E-04	3.65E-04	1.45E-03
GO:0007275~multicellular organism development	1.18E-06	2.82E+00	1.53E-03	1.53E-03	1.92E-03
GO:0009952~anterior/posterior pattern specification	1.63E-05	8.03E+00	2.09E-02	1.05E-02	2.65E-02
GO:0050965~detection of temperature stimulus involved in	4.04E-04	2.66E+01	4.09E-01	1.61E-01	6.58E-01
GO:0051965~positive regulation of synapse assembly	6.63E-04	8.56E+00	5.78E-01	1.94E-01	1.08E+00
GO:0048706~embryonic skeletal system development	9.45E-04	1.14E+01	7.08E-01	2.18E-01	1.53E+00
GO:0051216~cartilage development	1.36E-03	7.31E+00	8.30E-01	2.55E-01	2.20E+00
GO:0030324~lung development	1.52E-03	5.64E+00	8.61E-01	2.46E-01	2.45E+00
GO:0048704~embryonic skeletal system morphogenesis	2.18E-03	9.08E+00	9.42E-01	2.99E-01	3.50E+00
GO:0045944~positive regulation of transcription from RNA	2.20E-03	2.11E+00	9.43E-01	2.72E-01	3.53E+00
GO:0003700~transcription factor activity, sequence-specific DNA	2.63E-03	2.18E+00	5.96E-01	3.65E-01	3.53E+00
GO:0007389~pattern specification process	2.65E-03	8.61E+00	9.68E-01	2.92E-01	4.24E+00
GO:0019934~cGMP-mediated signaling	2.65E-03	3.75E+01	9.69E-01	2.70E-01	4.25E+00
GO:0031175~neuron projection development	2.70E-03	5.03E+00	9.70E-01	2.54E-01	4.32E+00
GO:0060426~lung vasculature development	3.39E-03	3.33E+01	9.88E-01	2.88E-01	5.39E+00
GO:0050729~positive regulation of inflammatory response	3.58E-03	7.93E+00	9.91E-01	2.84E-01	5.69E+00
GO:0016787~hydrolase activity	4.08E-03	1.79E+00	7.55E-01	3.75E-01	5.43E+00
GO:0033993~response to lipid	4.21E-03	3.00E+01	9.96E-01	3.07E-01	6.65E+00
GO:0071392~cellular response to estradiol stimulus	4.25E-03	1.21E+01	9.96E-01	2.93E-01	6.72E+00
GO:0001501~skeletal system development	4.34E-03	5.60E+00	9.97E-01	2.83E-01	6.86E+00
GO:0016337~single organismal cell-cell adhesion	4.51E-03	5.55E+00	9.97E-01	2.79E-01	7.12E+00
GO:0048306~calcium-dependent protein binding	4.70E-03	7.35E+00	8.02E-01	3.33E-01	6.22E+00
GO:0042803~protein homodimerization activity	5.30E-03	2.16E+00	8.39E-01	3.06E-01	6.99E+00
GO:0043065~positive regulation of apoptotic process	6.48E-03	2.98E+00	1.00E+00	3.60E-01	1.01E+01
GO:0035264~multicellular organism growth	7.02E-03	5.00E+00	1.00E+00	3.68E-01	1.09E+01
GO:0060411~cardiac septum morphogenesis	7.15E-03	2.31E+01	1.00E+00	3.59E-01	1.11E+01
GO:0051930~regulation of sensory perception of pain	7.84E-03	9.75E+00	1.00E+00	3.72E-01	1.21E+01
GO:0021756~striatum development	8.29E-03	2.14E+01	1.00E+00	3.76E-01	1.27E+01
GO:0007155~cell adhesion	9.44E-03	2.47E+00	1.00E+00	4.02E-01	1.43E+01
GO:0045779~negative regulation of bone resorption	1.08E-02	1.87E+01	1.00E+00	4.32E-01	1.62E+01
GO:0050872~white fat cell differentiation	1.08E-02	1.87E+01	1.00E+00	4.32E-01	1.62E+01
GO:0048286~lung alveolus development	1.21E-02	8.33E+00	1.00E+00	4.56E-01	1.80E+01
GO:0010862~positive regulation of pathway-restricted SMAD	1.21E-02	8.33E+00	1.00E+00	4.56E-01	1.80E+01
GO:0048468~cell development	1.28E-02	8.16E+00	1.00E+00	4.62E-01	1.89E+01
GO:0032331~negative regulation of chondrocyte differentiation	1.36E-02	1.67E+01	1.00E+00	4.70E-01	2.00E+01
GO:0048566~embryonic digestive tract development	1.36E-02	1.67E+01	1.00E+00	4.70E-01	2.00E+01
GO:0008083~growth factor activity	1.42E-02	4.20E+00	9.93E-01	5.58E-01	1.77E+01
GO:0006469~negative regulation of protein kinase activity	1.50E-02	5.26E+00	1.00E+00	4.92E-01	2.19E+01
GO:0033189~response to vitamin A	1.51E-02	1.58E+01	1.00E+00	4.83E-01	2.20E+01
GO:0030818~negative regulation of cAMP biosynthetic process	1.51E-02	1.58E+01	1.00E+00	4.83E-01	2.20E+01
GO:0004725~protein tyrosine phosphatase activity	1.53E-02	5.23E+00	9.95E-01	5.30E-01	1.89E+01
GO:0043401~steroid hormone mediated signaling pathway	1.58E-02	7.54E+00	1.00E+00	4.87E-01	2.29E+01
GO:0007507~heart development	1.58E-02	3.06E+00	1.00E+00	4.77E-01	2.29E+01
GO:0005178~integrin binding	1.69E-02	5.07E+00	9.97E-01	5.19E-01	2.07E+01
GO:0046965~retinoid X receptor binding	1.78E-02	1.45E+01	9.98E-01	4.96E-01	2.17E+01
GO:0032355~response to estradiol	1.84E-02	4.95E+00	1.00E+00	5.19E-01	2.61E+01
GO:0010628~positive regulation of gene expression	1.87E-02	2.50E+00	1.00E+00	5.15E-01	2.65E+01
GO:0009887~organ morphogenesis	2.36E-02	4.58E+00	1.00E+00	5.88E-01	3.22E+01
GO:0042572~retinol metabolic process	2.36E-02	1.25E+01	1.00E+00	5.78E-01	3.23E+01
GO:0008217~regulation of blood pressure	2.39E-02	6.45E+00	1.00E+00	5.73E-01	3.26E+01
GO:0030326~embryonic limb morphogenesis	2.49E-02	6.34E+00	1.00E+00	5.79E-01	3.38E+01
GO:0050905~neuromuscular process	2.74E-02	1.15E+01	1.00E+00	6.04E-01	3.65E+01
GO:0035529~NADH pyrophosphatase activity	2.91E-02	6.76E+01	1.00E+00	6.38E-01	3.32E+01
GO:0005006~epidermal growth factor-activated receptor activity	2.91E-02	6.76E+01	1.00E+00	6.38E-01	3.32E+01
GO:0090102~cochlea development	3.36E-02	1.03E+01	1.00E+00	6.71E-01	4.27E+01
GO:0006351~transcription, DNA-templated	3.37E-02	1.48E+00	1.00E+00	6.64E-01	4.29E+01
GO:0007399~nervous system development	3.53E-02	2.38E+00	1.00E+00	6.72E-01	4.44E+01

Term	PValue	Enrichment FC	Bonferroni	Benjamini	FDR
GO:0045746~negative regulation of Notch signaling pathway	3.79E-02	9.67E+00	1.00E+00	6.90E-01	4.68E+01
GO:0004551~nucleotide diphosphatase activity	3.86E-02	5.07E+01	1.00E+00	7.08E-01	4.16E+01
GO:0003708~retinoic acid receptor activity	3.86E-02	5.07E+01	1.00E+00	7.08E-01	4.16E+01
GO:0004528~phosphodiesterase I activity	3.86E-02	5.07E+01	1.00E+00	7.08E-01	4.16E+01
GO:0055098~response to low-density lipoprotein particle	3.92E-02	5.00E+01	1.00E+00	6.94E-01	4.80E+01
GO:0021569~rhombomere 3 development	3.92E-02	5.00E+01	1.00E+00	6.94E-01	4.80E+01
GO:0007218~neuropeptide signaling pathway	4.02E-02	5.26E+00	1.00E+00	6.95E-01	4.88E+01
GO:0050679~positive regulation of epithelial cell proliferation	4.02E-02	5.26E+00	1.00E+00	6.95E-01	4.88E+01
GO:0048678~response to axon injury	4.02E-02	9.37E+00	1.00E+00	6.87E-01	4.88E+01
GO:0019233~sensory perception of pain	4.15E-02	5.19E+00	1.00E+00	6.91E-01	5.00E+01
GO:0050873~brown fat cell differentiation	4.49E-02	8.81E+00	1.00E+00	7.12E-01	5.28E+01
GO:0007631~feeding behavior	4.49E-02	8.81E+00	1.00E+00	7.12E-01	5.28E+01
GO:0008284~positive regulation of cell proliferation	4.50E-02	2.03E+00	1.00E+00	7.06E-01	5.29E+01
GO:0048661~positive regulation of smooth muscle cell proliferation	4.57E-02	5.00E+00	1.00E+00	7.04E-01	5.34E+01
GO:0005515~protein binding	4.68E-02	1.26E+00	1.00E+00	7.47E-01	4.80E+01
GO:0004721~phosphoprotein phosphatase activity	4.80E-02	3.65E+00	1.00E+00	7.28E-01	4.89E+01
GO:0047429~nucleoside-triphosphate diphosphatase activity	4.81E-02	4.06E+01	1.00E+00	7.02E-01	4.89E+01
GO:0060438~trachea development	4.88E-02	4.00E+01	1.00E+00	7.21E-01	5.58E+01
GO:0061156~pulmonary artery morphogenesis	4.88E-02	4.00E+01	1.00E+00	7.21E-01	5.58E+01
